# Substrate-Controlled Divergent Reactivity of 2‑Alkynylindoles: [2 + 2] Cycloaddition–Retroelectrocyclization versus Tricyanovinylation for the Synthesis of NLOphores

**DOI:** 10.1021/acs.joc.6c00004

**Published:** 2026-06-11

**Authors:** Hazal Kayas, Yagmur Unal, Kubra Erden, Alberto Barsella, Onur Şahin, Cagatay Dengiz

**Affiliations:** † Department of Chemistry, 52984 Middle East Technical University, 06800 Ankara, Turkey; ‡ Département d’Optique Ultra-Rapide et Nanophotonique, 129756 IPCMS-CNRS, 23 Rue du Loess, BP 43, 67034 Strasbourg, Cedex 2, France; § Department of Occupational Health & Safety, Faculty of Health Sciences, 157915 Sinop University, 57000 Sinop, Turkey

## Abstract

This study provides insight into the donor characteristics of the indole framework and allows a comparison between its C-2 and C-3 positions. Ten electron-rich alkynes incorporating an indole core were synthesized via the Sonogashira cross-coupling reaction. Structural variations within these substrates led to two distinct reaction pathways for the formation of cyano-containing conjugated systems, yielding eight tricyanovinylation and two [2 + 2] cycloaddition–retroelectrocyclization (CA–RE) products. The tricyanovinylation products formed with tetracyanoethylene (TCNE) were obtained in 40–82% yields, whereas the chromophores produced through the [2 + 2] CA–RE pathway arising from alkyne activation were isolated in 30–63% yields. They display pronounced intramolecular charge transfer (ICT), with λ_max_ values ranging from 494 to 504 nm for the tricyanovinylation products, while the two [2 + 2] CA–RE chromophores absorb at 471 and 495 nm. The observed ICT bands are supported by UV/vis studies by positive solvatochromism and protonation experiments. To clarify the relationship between NLO response and ICT properties, the dipole moment, band gap, electronegativity, average global hardness–softness, average polarizability, and first hyperpolarizability parameters were evaluated using computational methods. In addition to theoretical DFT calculations, the EFISHG technique was employed to investigate the NLO properties. The experimental μβ values of the selected molecules range from 150 to 560 × 10^–48^ esu.

## Graphical abstract

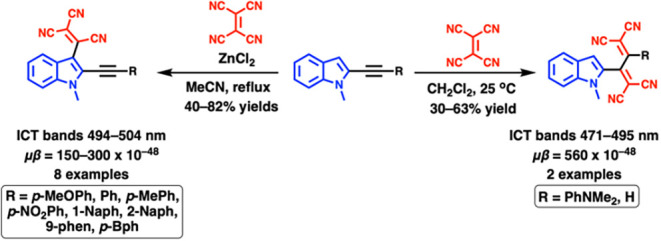

## Introduction

Owing to the rapidly expanding range of applications and the significant insights gained in recent years into their structure–property relationships, D–A type π-conjugated chromophores are currently attracting unprecedented attention.^1,2^ These systems, formed by the combination of donor, acceptor, and π-bridge units, play a key role in a wide range of important applications, including organic light-emitting diodes (OLEDs),^3^ dye-sensitized solar cells (DSSCs),^4^ organic field-effect transistors (OFETs),^5^ biosensors,^6^ photothermal/photoacoustic materials,^7,8^ and nonlinear optics (NLO).^9^ A careful examination of the literature reveals that, while numerous studies have focused on varying donor and acceptor strengths,^10^ the majority of systematic investigations have primarily centered on modifying the π-bridge length.^11,12^ However, a recent trend has emerged toward the exploration of cyano-rich systems, where cyano groups serve as effective acceptor units.^13^ This growing interest mainly arises from their strong electron-withdrawing capability,^14^ ability to improve the planarity of the π-conjugated backbone,^15^ effective lowering of the LUMO energy level without notably affecting the HOMO,^16^ and their contribution to chemical and thermal stabilities.^17^ In addition to these properties, the incorporation of cyano-containing acceptor units into conjugated systems is further facilitated by well-established synthetic protocols, such as Michael-type additions^18^ and Knoevenagel condensations,^19^ which has significantly increased their appeal in research.^20^ While Knoevenagel condensation with malononitrile generates dicyanovinyl frameworks,^21^ Michael-type additions between tetracyanoethylene (TCNE) and nucleophiles afford tricyanovinyl derivatives.^22^ Beyond dicyanovinyl and tricyanovinyl systems, the incorporation of additional cyano groups can be efficiently achieved via click-type [2 + 2] cycloaddition–retroelectrocyclization (CA–RE) reactions.^23–29^ The resulting tetracyanobutadiene (TCBD) frameworks have been extensively investigated due to their outstanding nonlinear optical properties.^9,30–32^ Owing to their high efficiency, click-type [2 + 2] CA–RE reactions have also been utilized as versatile postpolymerization modification methods, enabling the introduction of TCBD units into polymer backbones.^33–35^ Furthermore, the strong intramolecular charge-transfer (ICT) nature of TCBD-based chromophores has enabled their use in a wide range of applications, including Aviram–Ratner-type molecular dyads,^36^ ion sensors,^37^ DSSCs,^38,39^ and fluorescent materials.^40,41^ Despite their broad range of applications, the [2 + 2] CA–RE reactions leading to TCBD frameworks generally require substrates bearing strongly electron-donating groups, which inherently restricts the substrate scope.^42^ To address this limitation, several research groups, including ours, have recently made significant progress by developing new donor-functionalized alkyne systems such as hydrazone-,^9^ azulene-,^43^ urea-,^44^ triazene,^45^ and ynamide-based derivatives.^46^ In our previous study on 3-alkynylindoles,^31^ these donor-substituted substrates were shown to undergo [2 + 2] CA–RE with remarkably high yields, affording push–pull-type dyes that exhibited excellent NLO responses.

Herein, we extend this approach to the synthesis of new donor-substituted substrates in which the alkyne moiety is positioned at the 2-position of the indole ring, a site known to be more challenging to functionalize compared with the 3-position (Scheme 1). The reactivity of these 2-alkynylindoles toward TCNE in [2 + 2] CA–RE reactions was systematically investigated. In particular, we examined whether the unoccupied 3-position of the indole ring interferes with the desired [2 + 2] CA–RE process and evaluated how different donor groups attached to the alkyne units influence this potential competitive reactivity. The NLO behavior of selected chromophores was subsequently explored both experimentally, using the electric-field-induced second-harmonic-generation (EFISHG) technique, and theoretically through computational studies.

**Scheme 1:**
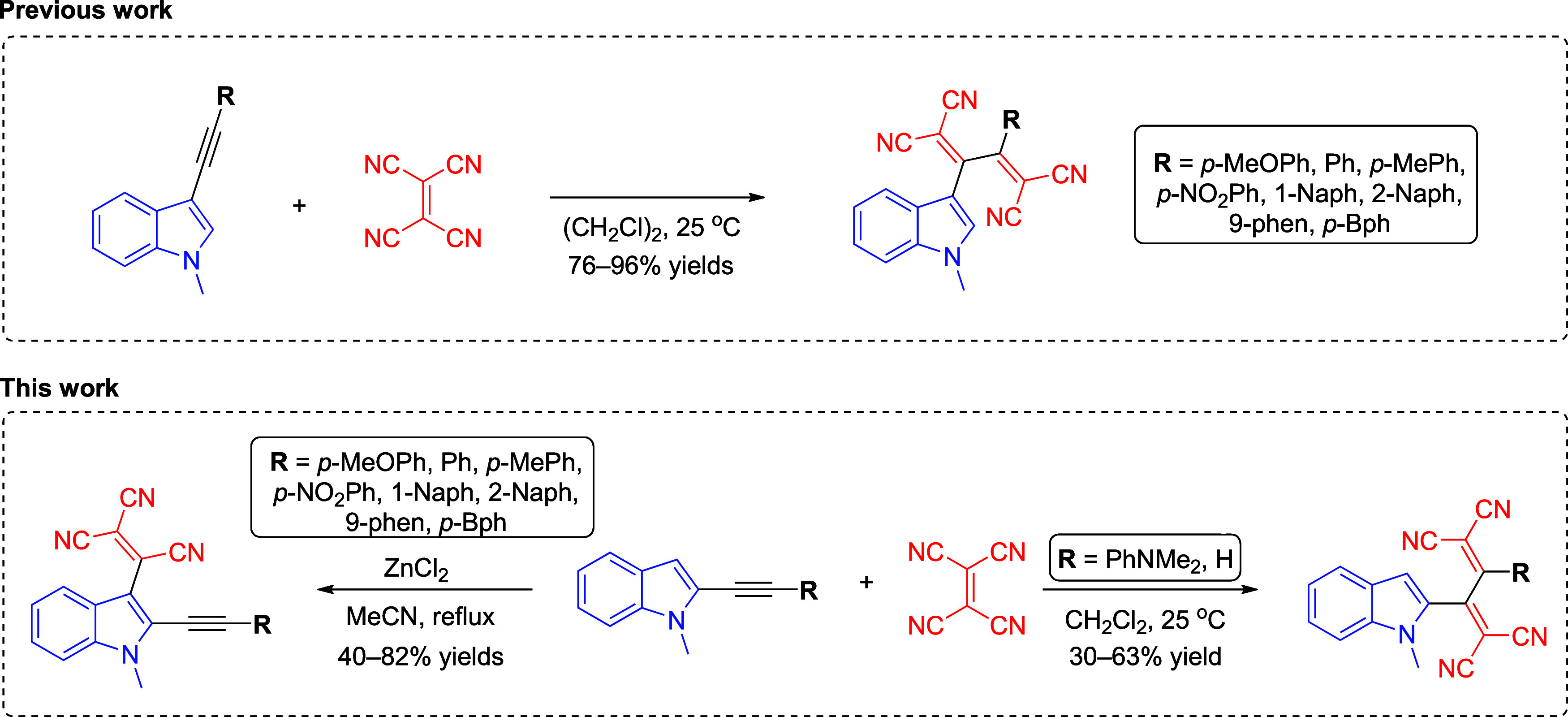
Comparative Overview of Earlier and Present Studies on the Reactions of Alkynylindoles with TCNE, Emphasizing the Side-Chain-Dependent Changes in Reaction Paths

## Results and Discussion

### Synthesis and Characterizations

In the first stage of this study, we focused on the synthesis of terminal alkyne **6**, which serves as the key intermediate for the preparation of the substrates to be used in subsequent reactions with TCNE. Previous reports have demonstrated that when the terminal alkyne is positioned at C-3, the resulting structures suffer from significant stability issues, highlighting the challenges associated with accessing such derivatives.^31,47^ Commercially available indole (**1**) was first methylated in DMF using KOH and MeI to afford *N*-methylindole (**2**) (Scheme 2).^48^ Subsequently, the corresponding iodoindole **3**, required for the following Sonogashira cross-coupling reactions, was obtained by treating compound **2** with *n*-BuLi followed by I_2_.^49^ The Sonogashira cross-coupling reaction between compound **3** and TMS-acetylene **4**, followed by subsequent TMS deprotection, afforded the target terminal alkyne **6** in 91% yield, without any observed stability issues. With substrate **6** in hand, the reactions performed with the electron-rich aryl iodides **7c** and **7d** in NEt_3_ at room temperature smoothly afforded the corresponding substituted alkynylindoles **8c** and **8d** in 74% and 66% yields, respectively. In contrast, the aryl iodides bearing NO_2_ and Me substituents required elevated temperatures, and the reactions carried out in toluene/DIPA at 60 °C delivered the desired products **8a** and **8b** in 73% and 70% yields, respectively.

**Scheme 2:**
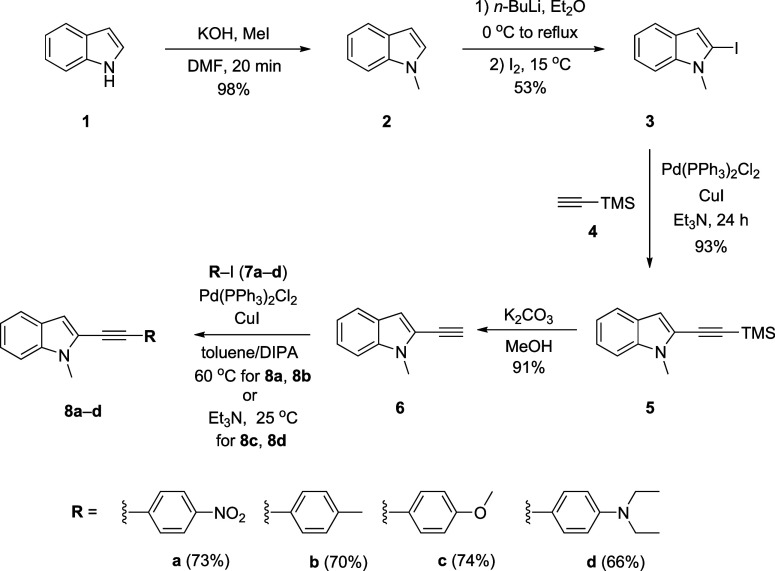
Synthesis of 2-Alkynylindoles **8a**–**d**

For the synthesis of the second part of 2-alkynylindoles **8e**–**i** containing various aromatic units (phenyl, 2-naphthyl, 1-naphthyl, phenanthrene, and biphenyl), Sonogashira cross-coupling reactions were performed using the corresponding alkyne moieties **9e**–**i** and compound **3**. The target compounds **8e**–**i** were obtained in 41–78% yields (Scheme 3). This method was preferred because the aryl bromides bearing these groups are commercially available, whereas these compounds do not undergo efficient cross-coupling to the same extent as iodoarenes. All the obtained alkynes were found to be stable under ambient conditions. The well-known stability issue, which is considered the main limitation for the use of alkynes in [2 + 2] CA–RE reactions according to the literature,^31^ does not pose any problem for this series of alkynes.

**Scheme 3:**
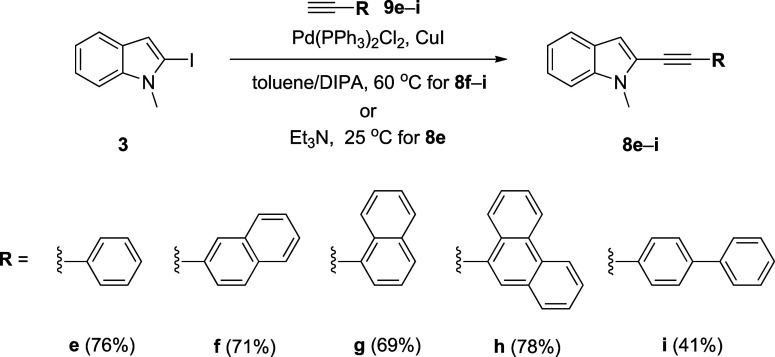
Sonogashira Cross-Coupling Reactions of Iodoindole **3** with Alkynes **9e**–**i**

After the preparation of all substrates, the phenyl-capped alkyne **8e** was selected as a representative to examine whether the alkyne moiety at the C-2 position of the substituted indole could be activated for [2 + 2] CA–RE reactions. When the reaction with TCNE was performed at room temperature in CH_2_Cl_2_, a large portion of the starting material remained unreacted, and the desired TCBD product **11** was not observed (Scheme 4). A red spot appeared during TLC (SiO_2_) analysis although no red-colored product was observed in solution. This observation suggested that the reaction might have occurred in the presence of SiO_2_. Upon the addition of approximately 10 g of SiO_2_ (excess) to the reaction mixture (starting material **8e**: 40 mg, 0.17 mmol), the solution color rapidly turned red, and the starting material was significantly consumed. The tricyanovinylation product was formed, albeit in a low yield of ∼16%.

**Scheme 4:**
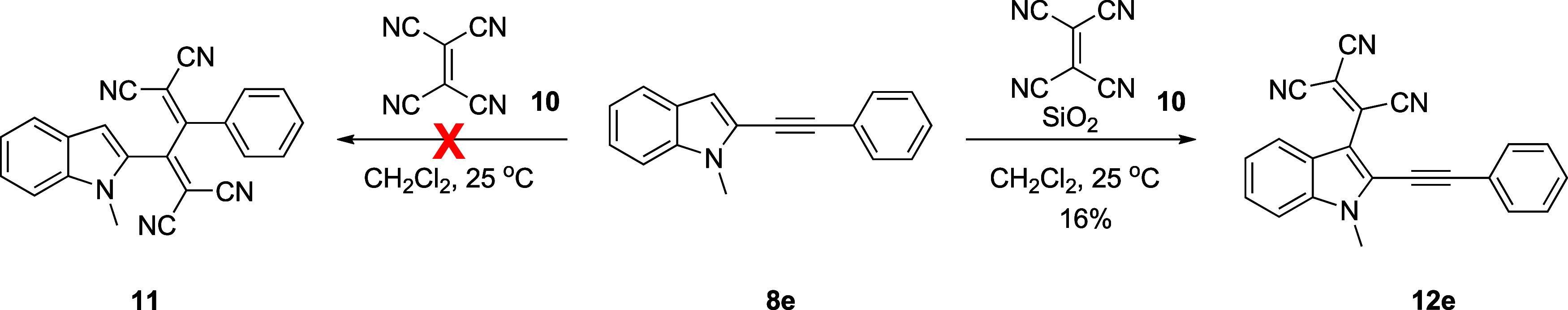
Preliminary Attempts for the [2 + 2] CA–RE between Alkyne **8e** and TCNE **10**

In our earlier work, we demonstrated that alkyne-containing substrates unreactive toward TCNE under ambient conditions could be efficiently driven into [2 + 2] CA–RE reactions in the presence of a Lewis acid catalyst.^50^ Consistent with our findings, the literature also reports the use of Lewis acids to promote cycloaddition processes involving TCNE.^51,52^ For this purpose, trials conducted in MeCN under reflux in the presence of Lewis acids LiClO_4_ and ZnCl_2_ did not activate the desired [2 + 2] CA–RE pathway; however, the corresponding tricyanovinylation products were obtained in 34% and 61% yields, respectively (see also Scheme S1 for the proposed mechanisms). As no improved results were achieved in additional experiments performed in various solvents and at different temperatures, the derivatization step was carried out by reacting the 2-substituted indoles with TCNE in MeCN under reflux in the presence of ZnCl_2_ (Scheme 5).

**Scheme 5:**
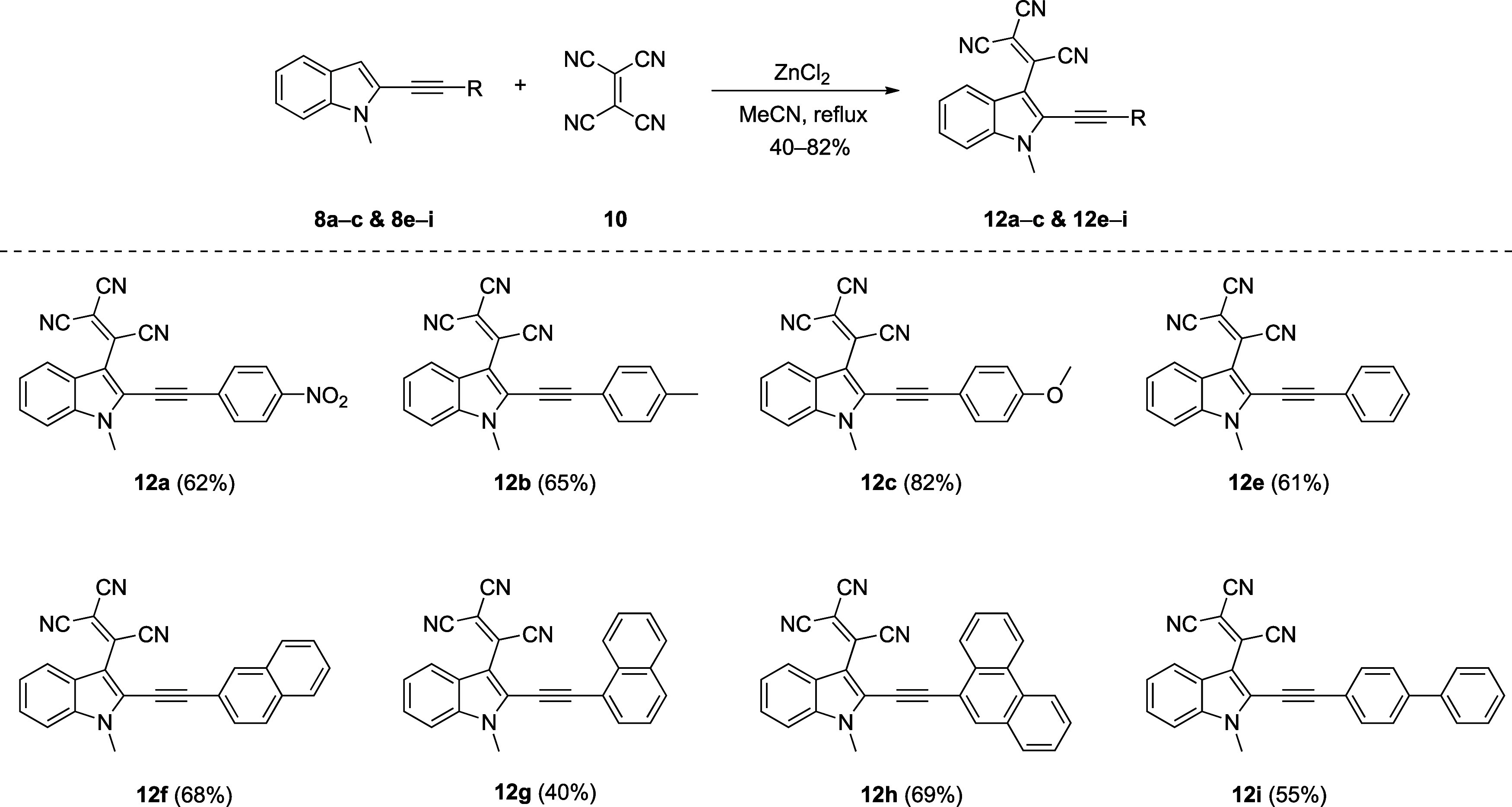
Tricyanovinylation Reactions of **8a**–**c** and **8e**–**i** with TCNE

Evaluation of the results indicates that, apart from the donor methoxy group (82% yield), various substituents did not exert a significant influence on the tricyanovinylation yields. The enhanced yield observed for the methoxy-substituted derivative can be attributed to the mesomeric electron-donating effect of the methoxy group, which increases the electron density on the indole ring. The relatively lower yield obtained for the 1-naphthyl derivative (40% yield) is likely due to solubility issues encountered with this substrate. The fact that the reactions between TCNE and 2-substituted alkynylindoles proceed through the tricyanovinylation pathway is evidenced by the disappearance of the singlet signal at the C-3 position after the reaction. For derivative **12i** (CCDC 2516047), structural characterization was further confirmed by X-ray crystallography, which clearly verified the proposed structure (Figure 1). Single crystals of **12i** suitable for X-ray analysis were obtained through slow evaporation from a solution of DCM/*n*-hexane at 25 °C.

**Figure 1:**
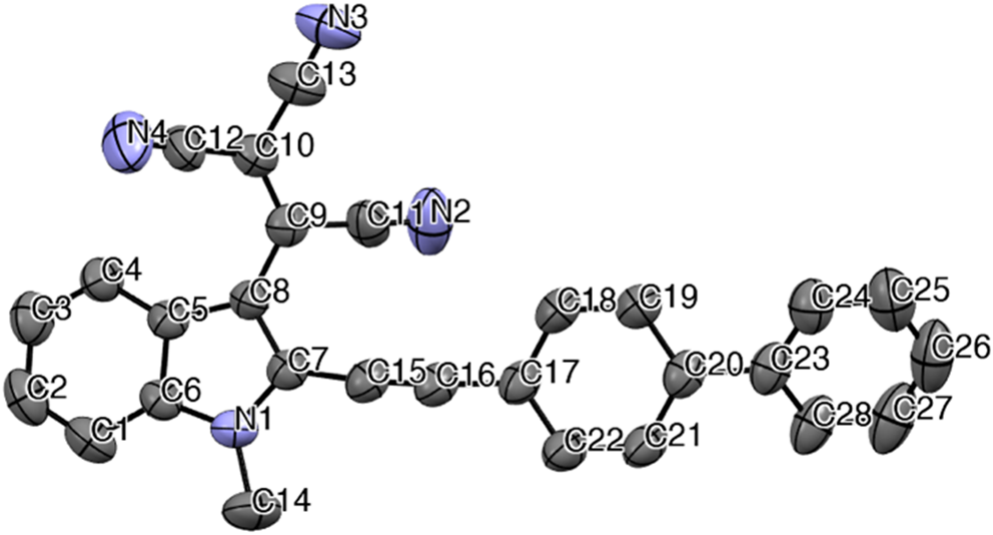
ORTEP representation of **12i** with vibrational ellipsoids shown at the 50% probability level, arbitrary numbering. H atoms and solvent molecules are omitted for clarity.

The initial results indicate that the synthesized substrates contain two reactive units and that tricyanovinylation at the C-3 position is the preferred pathway for the substrates employed. At this stage, it remained an open question whether access to the targeted TCBD derivatives, the [2 + 2] CA–RE products, would be feasible. In this context, we examined the reactions of alkynes bearing the strongly electron-donating *N*,*N*-diethylaniline group with TCNE. As anticipated, in this case, the [2 + 2] CA–RE pathway became accessible, yielding the desired TCBD product in 63% yield (Scheme 6). Likewise, the reaction of the unsubstituted terminal alkyne **6** with TCNE also afforded the [2 + 2] CA–RE product. These findings demonstrate that steric effects imposed by substituents divert the reaction pathway toward tricyanovinylation. The tricyanovinylation attempts on compounds **6** and 1**4**, carried out with TCNE in MeCN under reflux conditions in the presence of ZnCl_2_, did not yield any positive results. This outcome is attributed to the presence of the TCBD unit, which likely sterically blocks the 3-position of the indole while also reducing its reactivity by drawing the electron density away from the indole core.

**Scheme 6:**
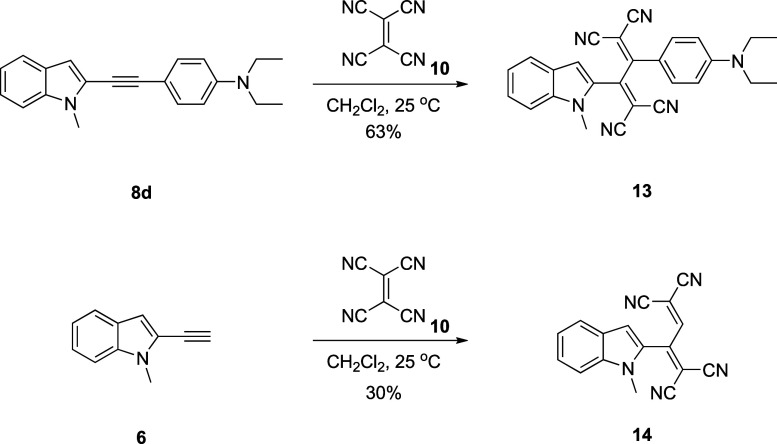
[2 + 2] CA–RE Reactions of **8d** and **6** with TCNE **10**

### UV/Vis Spectroscopy

In this stage of the study, spectral data were collected using a UV/vis spectrophotometer to determine the molar absorptivities (*ε*) and electronic transition energies of the synthesized molecules in CH_2_Cl_2_ solutions. All synthesized compounds exhibit colored solutions under ambient conditions (Figure S83). This, together with the donor properties of the indole units and the strongly electron-accepting character of the cyano-rich segments, suggests the presence of ICT, which was further examined through UV/vis spectroscopy. While the UV/vis spectra of all synthesized compounds are provided in Figure S79, here we present the spectra of five representative compounds: **12a**, **12c**, **12e**, **13**, and **14** (Figure 2). For the tricyanovinylation products **12a**, **12c**, and **12e**, *ε* values range from 1.41 × 10^–4^ to 1.85 × 10^–4^ M^–1^cm^–1^, while the λ_max_ values fall between 492 and 504 nm. As expected, the presence of an electron-withdrawing nitrobenzene group leads to the lowest λ_max_ around 492 nm (ε *=* 1.41 × 10^–4^ M^–1^ cm^–1^ for **12a**), whereas the unsubstituted phenyl group shows a λ_max_ of 496 nm (ε *=* 1.79 × 10^–4^ M^–1^ cm^–1^ for **12e**). In contrast, the electron-rich methoxy-substituted phenyl unit induces a bathochromic shift, increasing the λ_max_ up to 506 nm (ε *=* 1.85 × 10^–4^ M^–1^ cm^–1^ for **12c**). These data clearly support the presence of ICT and show that the λ_max_ values respond to the electronic nature of the substituents.

**Figure 2:**
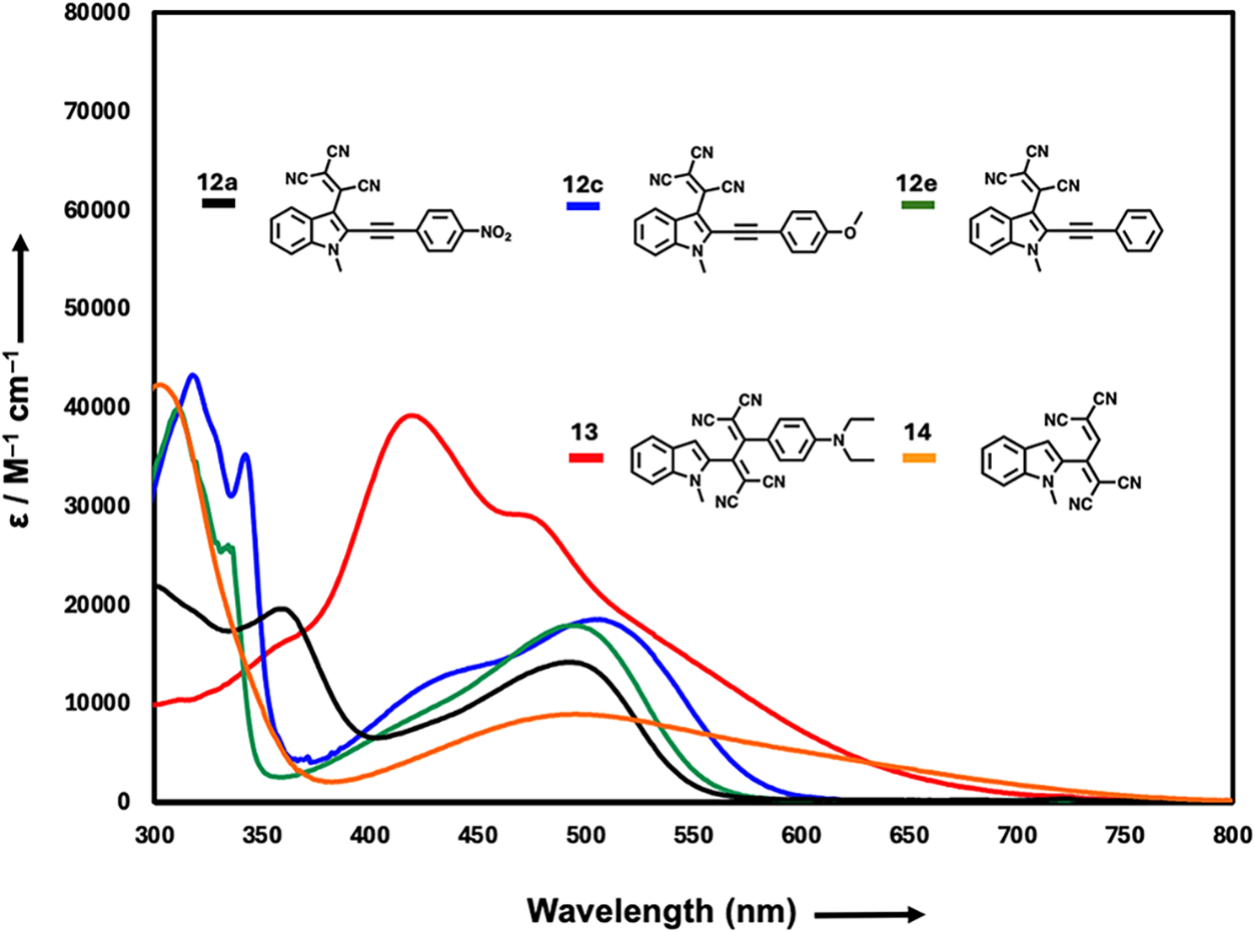
UV/vis spectra of chromophores **12a**, **12c**, **12e**, **13**, and **14** in CH_2_Cl_2_ at 25 °C.

Focusing on the TCBD derivatives **13** and **14** reveals distinct absorption behaviors. Chromophore **13** exhibits two separate absorption bands, the first at 419 nm (ε = 3.91 × 10^–4^ M^–1^ cm^–1^) and the second at 471 nm (ε = 2.91 × 10^–4^ M^–1^ cm^–1^). These two bands are attributed to independent charge-transfer processes: one originating from the electron-rich diethylaniline unit and the other from the indole donor, both transferring electron density to the TCBD acceptor moiety. In contrast, chromophore **14**, which contains only the indole donor group, displays a single ICT band at 495 nm (ε = 0.89 × 10^–4^ M^–1^ cm^–1^), supporting this interpretation. To gain further insight into the observed ICT bands, solvatochromism experiments were performed. For these measurements, the tricyanovinylation product **12e** and the [2 + 2] CA–RE product **13** were selected (Figure 3). In both compounds, solutions were prepared in varying ratios of CH_2_Cl_2_ to *n*-hexane, and the resulting measurements showed clear bathochromic shifts, demonstrating positive solvatochromism characteristic of ICT systems (Figure 3).^53^ The observed positive solvatochromism arises because the excited states are selectively stabilized by polar solvents relative to the ground states, leading to a bathochromic shift in the absorption maxima.^54^ A slight yet discernible bathochromic shift was observed in trials using four solvents with different polarities (Figures S80 and S81).

**Figure 3:**
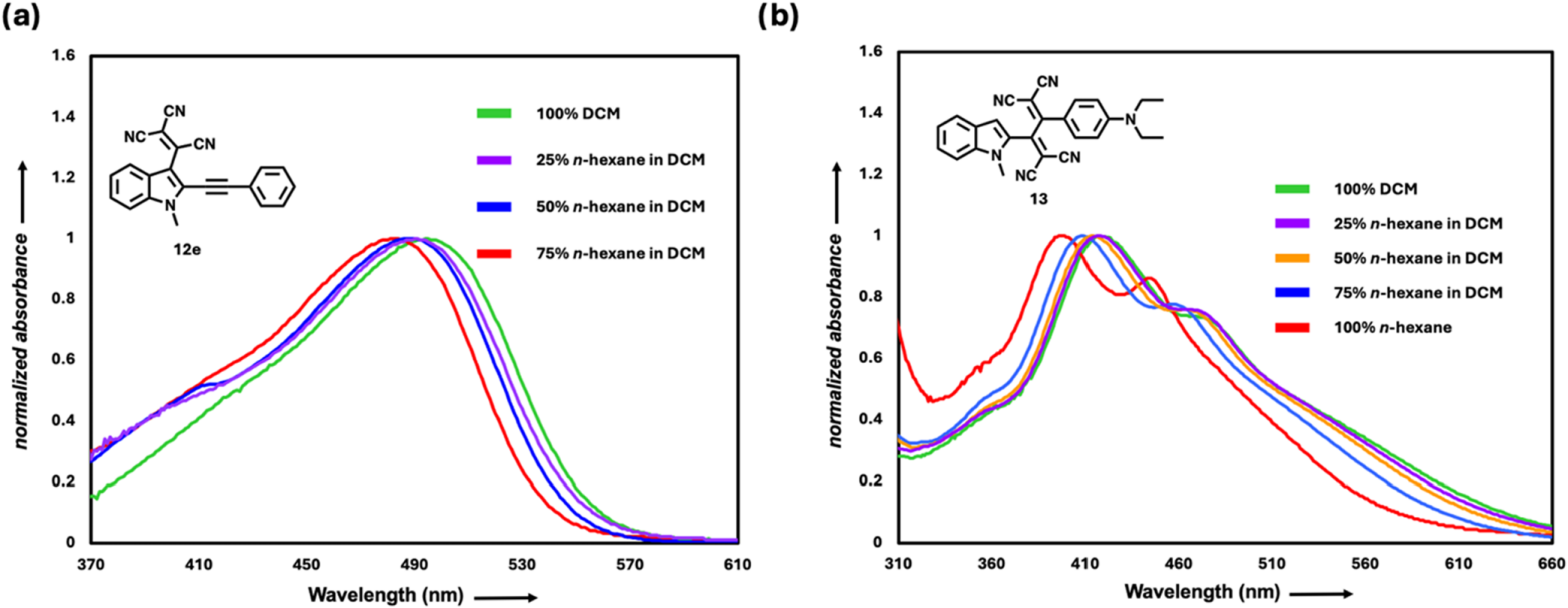
UV/vis spectra of (a) **12e** and (b) **13** in *n*-hexane/CH_2_Cl_2_ mixtures at 25 °C.

Additionally, protonation experiments commonly used to confirm ICT in the literature were performed (Figure 4). Initially, trifluoroacetic acid (TFA) was added to CH_2_Cl_2_ solutions of **12e** and **13** to examine any changes in the ICT absorption bands. While **12e** showed no response upon TFA addition, the absorption band of **13** was significantly affected because of quaternization of the diethylaniline group. The lack of response in **12e** is attributed to the lone pair on the indole nitrogen being nonreactive toward quaternization with TFA due to aromatic stabilization. Following the addition of TFA, neutralization with NEt_3_ partially restored the diminished ICT band in **13**. Although these results do not provide complete information about ICT between the indole and cyano groups, they clearly indicate a strong ICT interaction between the diethylaniline and the TCBD unit.

**Figure 4:**
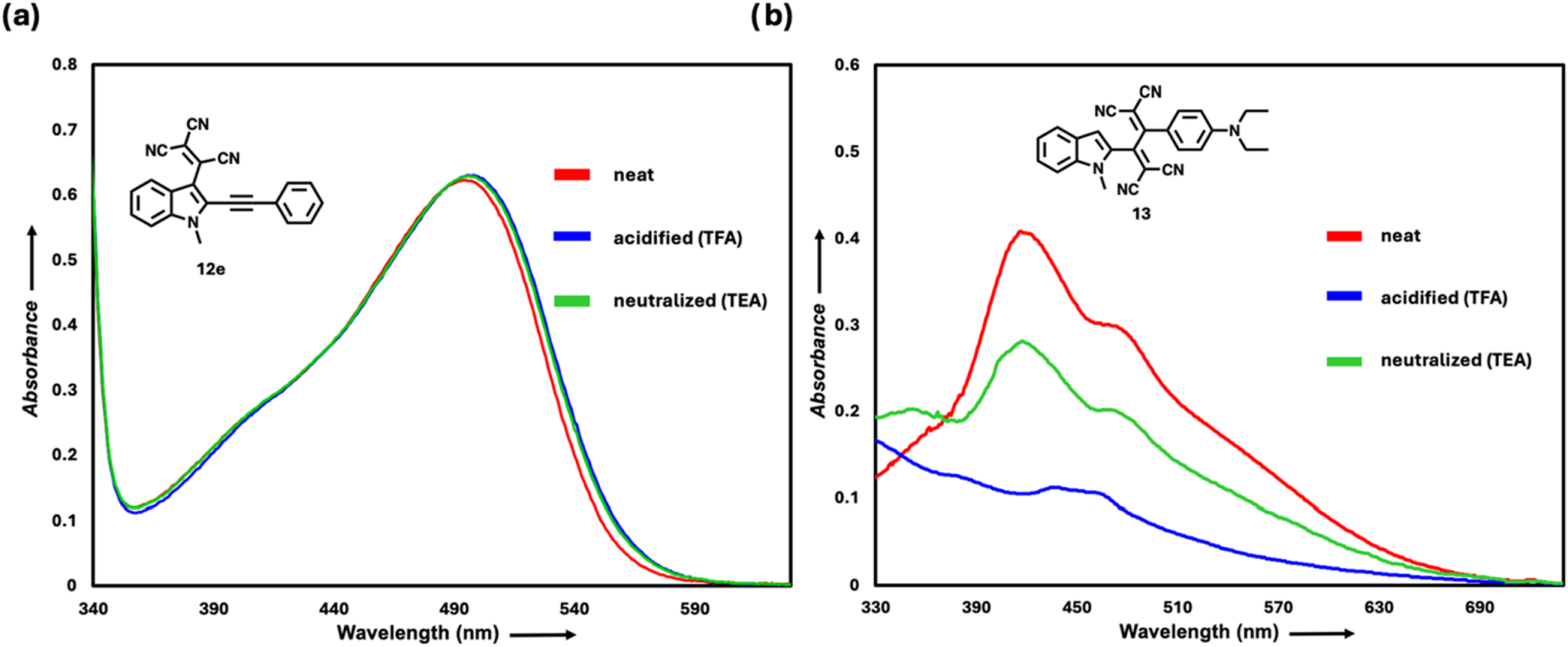
Quaternization–neutralization experiments of (a) **12e** and (b) **13**.

### Thermal Gravimetric Analysis (TGA)

Since NLO materials typically operate within the 100–200 °C temperature range, thermogravimetric analysis (TGA) was performed to evaluate the thermal stability of the designed chromophores prior to NLO measurements.^55,56^ For this purpose, two representative compounds, **12e** and **13**, were selected for TGA experiments (Figure 5, see also Figure S82 for the TGA curves of the remaining compounds). Compound **13** exhibits an early onset of mass loss, beginning around 115 °C, and continues to decompose almost linearly up to 930 °C, indicating limited thermal robustness. In contrast, **12e** remains remarkably stable up to approximately 200 °C, showing no detectable degradation in this temperature region, and subsequently undergoes mass loss at a significantly slower rate compared with compound **13**. To provide a clearer quantitative comparison of their thermal stabilities, the temperatures corresponding to 50% mass loss for both chromophores were compared. Compound **13** reaches 50% mass loss at 498 °C, whereas **12e** does not reach this point until 830 °C, demonstrating a substantial stability advantage for the latter. This pronounced difference is consistent with the optical band gaps of the two structures and aligns well with the frontier molecular orbital energy differences calculated in the following sections. Compound **13**, possessing a smaller HOMO–LUMO energy gap, is inherently less thermally stable, whereas the tricyanovinyl-based chromophore **12e** benefits from a more stabilized electronic structure. Overall, these results clearly support the superior thermal robustness of tricyanovinyl derivatives and reinforce their potential as promising NLOphore candidates for future optical material applications.

**Figure 5:**
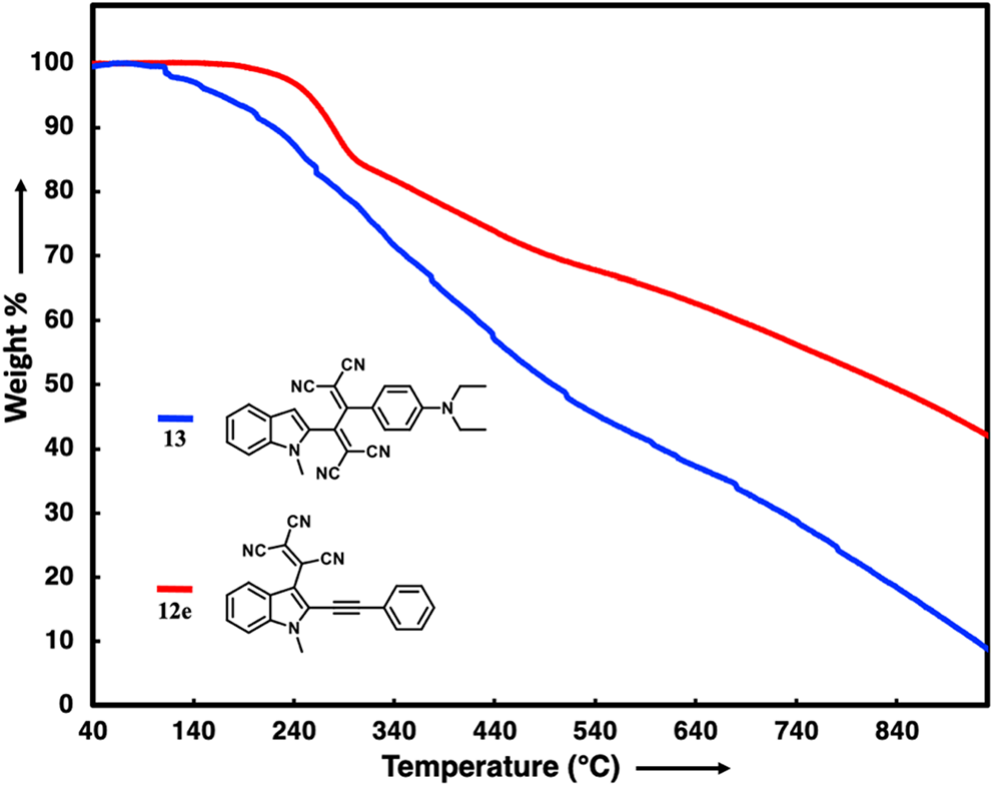
Thermogravimetric analysis curves of **12e** (red line) and **13** (blue line).

### Computational Studies

While the previous sections describe the experimental measurements regarding the optical properties of the synthesized compounds, this part further elaborates on these findings with the support of computational studies. The computational studies presented in this section focus on frontier molecular orbital (FMO) depictions, ICT characteristics, molecular stability, and NLO properties. Following a manual conformational search, conformers located within a 3 kcal mol^–1^ energy window relative to the global minimum were chosen for DFT computations. DFT calculations were carried out using Gaussian 16 Rev. C.01 ^57^ at the CAM-B3LYP/6–31++G­(d,p) level of theory with CPCM solvation in CH_2_Cl_2_. First, to gain a qualitative understanding of the ICT, we utilized HOMO–LUMO orbital visualizations and electrostatic potential (ESP) analyses (Figure 6).^58^ For chromophore **12e**, the HOMO is generally delocalized over the entire molecule, while the LUMO is predominantly localized on the tricyanovinyl moiety. In chromophore **13**, the HOMO is localized on the strong donor diethylaniline group, whereas the LUMO, similar to **12e**, is concentrated on the TCBD unit. This is consistent with the expected behavior of ICT systems. Similarly, analysis of the ESP maps shows that the red regions are concentrated on the cyano-rich areas, while the blue regions, corresponding to low electron density, are associated with the donor groups.

**Figure 6:**
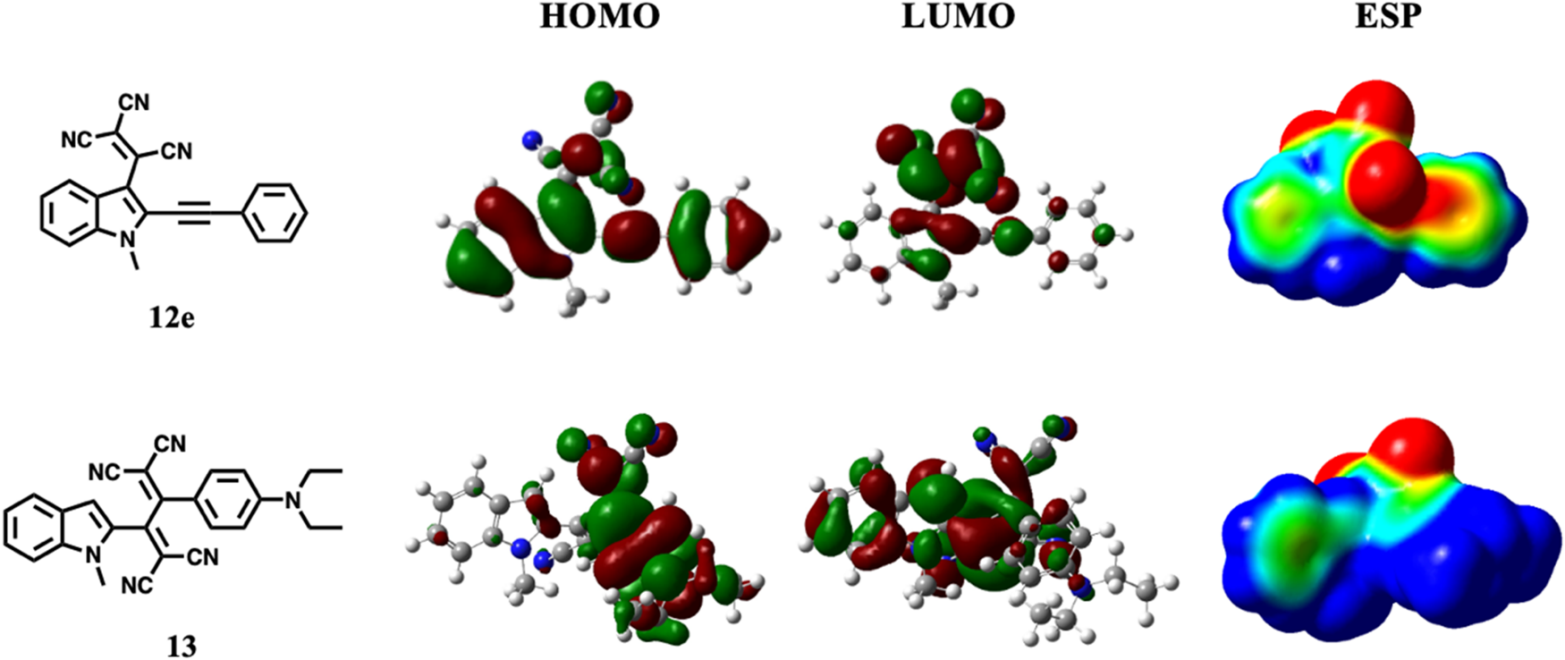
HOMO, LUMO, and electrostatic potential (ESP) surfaces of **12e** and **13**.

Representative chromophores **12e** and **13** and their frontier orbital energy gaps are summarized in Figure 7. The HOMO–LUMO energy gaps were determined using two different approaches: (1) the direct energy difference calculated from the optimized ground state (Δ*E* ^direct^) and (2) the vertical excitation energy corresponding to the lowest singlet excited state (Δ*E* ^TD^). Since the second approach shows better agreement with the experimental data, the values presented in Figure 7 were obtained using this method. Based on these findings, chromophore **12e** displays a HOMO–LUMO energy gap of 2.65 eV, while the corresponding gap for **13** rises to 2.97 eV. The good agreement with UV/vis measurements confirms that **12e** exhibits a stronger ICT character relative to **13**.

**Figure 7:**
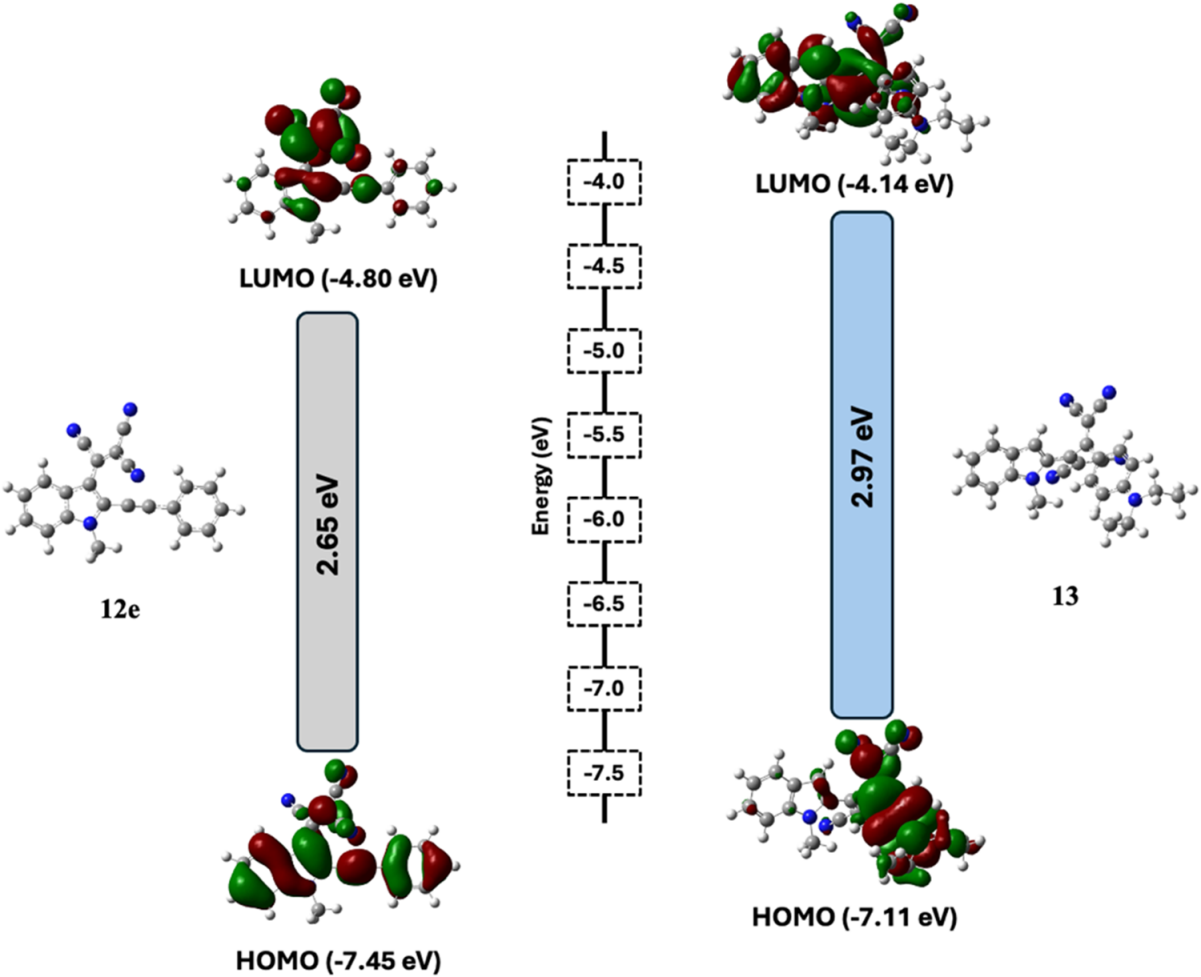
Energy-level representation of the frontier orbitals calculated for chromophores **12e** and **13**.

In this section of the study, prior to discussing the experimental NLO measurements, the electric dipole moments (μ) (eq 1), HOMO–LUMO gap (Δ*E* ^TD^), and electronegativity (χ) (eq 2) values were calculated (Table 1). Additionally, the predicted stabilities of the target chromophores were evaluated through the calculation of their global hardness (η) and softness (σ) parameters, providing additional support to the thermal stability trends observed in the TGA analyses (eqs 3 and 4). Additionally, key electronic and optical descriptors, average polarizability [α_(tot)_], and first hyperpolarizability [β_(tot)_] were computed at the CAM-B3LYP/6–31G++(d,p) level of theory in CH_2_Cl_2_ using the CPCM solvation model (eqs 5, and 6). These theoretical analyses were carried out to establish the structure–property relationships that govern the nonlinear optical responses of chromophores **12a**, **12c**,**12e**, and **13** selected for experimental measurements

1 $$\mu ={\left[{\left(\mu_{x}\right)}^{2}+{\left(\mu_{y}\right)}^{2}+{\left(\mu_{z}\right)}^{2}\right]}^{1/2}$$

2 $$\chi =-1/2\left(E_{\mathrm{HOMO}}+E_{\mathrm{LUMO}}\right)$$

3 $$\eta =-1/2\left(E_{\mathrm{HOMO}}-E_{\mathrm{LUMO}}\right)$$

4 σ=1/η

5 $$\alpha =1/3\left(\alpha_{\mathrm{xx}}+\alpha_{\mathrm{yy}}+\alpha_{\mathrm{zz}}\right)$$

6 $$\beta ={\left[{\left(\beta_{\mathrm{xxx}}+\beta_{\mathrm{xyy}}+\beta_{\mathrm{xzz}}\right)}^{2}+{\left(\beta_{\mathrm{yyy}}+\beta_{\mathrm{xxy}}+\beta_{\mathrm{yzz}}\right)}^{2}+{\left(\beta_{\mathrm{zzz}}+\beta_{\mathrm{xxz}}+\beta_{\mathrm{yyz}}\right)}^{2}\right]}^{1/2}$$

**Table 1:** Electric Dipole Moment (μ), HOMO–LUMO gap (Δ*E* ^TD^), Electronegativity (χ), Global Chemical Hardness (η)–Softness (σ), Average Polarizability [*α*_(tot)_], and First Hyperpolarizability [β_(tot)_], Calculated at the CAM-B3LYP/6-31G++(d,p) Theory in CH_2_Cl_2_ (CPCM)

	*μ* (D)	*E*_HOMO_ (eV)	*E*_LUMO_ (eV)	Δ*E* ^TD^ (eV)	χ (eV)	η (eV)	σ (eV^–^)	α_(tot)_ (×10^–24^ esu)	*β*_((tot)_ (×10^–30^ esu)
**12a**	13.2385	–7.63	–4.94	2.69	6.28	1.35	0.74	69.294	134.879
**12b**	11.9304	–7.37	–4.75	2.62	6.06	1.31	0.76	67.594	78.393
**12c**	10.4343	–7.23	–4.67	2.57	5.95	1.28	0.78	69.424	103.351
**12e**	11.7266	–7.45	–4.80	2.66	6.13	1.33	0.75	64.307	71.995
**12f**	11.4570	–7.32	–4.71	2.61	6.01	1.30	0.77	76.621	71.848
**12g**	11.5570	–7.28	–4.70	2.58	5.99	1.29	0.78	75.011	67.406
**12h**	11.3298	–7.24	–4.66	2.58	5.95	1.29	0.77	86.700	73.457
**12i**	11.7844	–7.34	–4.72	2.62	6.03	1.31	0.76	81.192	85.660
**13**	18.4497	–7.11	–4.14	2.97	5.63	1.48	0.67	84.340	181.105

To account for the thermal stability observed in the TGA data, the global hardness (η) and softness (σ) indices were derived from the FMO energies. As a general trend, compounds with a larger HOMO–LUMO energy gap give higher hardness values, exhibiting greater kinetic stability. Consistent with the experimental results, the chemical hardness values within the **12a**–**i** series range from 1.28 to 1.35, whereas compound **13**, which possesses a higher Δ*E* ^TD^ value, exhibits an η value of up to 1.48. In contrast, an opposite trend is observed for the softness parameters (σ = 0.74–0.78 for **12a**–**i** and 0.67 for **13**). The dipole moment analysis further reveals that compound **13** possesses a significantly higher dipole moment (μ = 18.4497 D) compared to the **12a**–**i** series (μ = 10.4343–13.2385 D), a feature that is expected to play an important role in its enhanced NLO response. A close examination of the **12a**–**i** series reveals that the presence of the electron-deficient NO_2_ group (μ = 13.2385 D for **12a**) leads to the highest dipole moment within the series, whereas incorporation of the electron-rich OMe group results in the lowest dipole moment (μ = 10.4343 D for **12c**). Similarly, examination of the **12a**–**i** series and compound **13** reveals a clear correlation between electronegativity and dipole moment. Compound **12a**, bearing the electron-withdrawing NO_2_ group, exhibits the highest electronegativity (χ = 6.28 eV) and correspondingly the largest dipole moment within the series. In contrast, compound **13**, substituted with the electron-donating NMe_2_ group, shows the lowest electronegativity (χ = 5.63 eV). The remaining compounds (**12b**–**i**) display intermediate values of χ and dipole moments, reflecting the relative electron-withdrawing or electron-donating character of their substituents. These results indicate that the electronic nature of the substituents strongly influences the charge distribution and polarity of the chromophores, which is consistent with their predicted ICT behavior.

Similarly, the nonlinear optical properties of compounds **12a**–**i** and **13** are closely related to their electronic structures. Compound **13** with strong donor–acceptor interactions exhibits the highest dipole moment (μ = 18.4497), resulting in enhanced average polarizability [α_(tot)_ = 84.340 × 10^–24^ esu] and first hyperpolarizability [β_(tot)_ = 181.105 × 10^–30^ esu]. In contrast, compound **12a** bearing strong electron-withdrawing groups shows moderate μ, leading to lower β_(tot)_ [= 134.879 × 10^–30^ esu] despite its higher electronegativity (χ = 6.28 eV). These results indicate that the combination of high dipole moment, suitable HOMO–LUMO gap, and electron-rich substituents promotes efficient ICT, enhancing the NLO response of the chromophores.

### NLO Studies

Based on the general structural features of the cyano-rich chromophores and the first hyperpolarizability values obtained from calculations, experimental NLO measurements were carried out for four selected chromophores **12a**, **12c**, **12e**, and **13**. Experimental NLO measurements were performed using the electric-field-induced second-harmonic-generation (EFISHG) technique. This method allows the evaluation of the molecular quadratic response in solution under the influence of an applied electric field. In EFISHG experiments, the measurable quantity is the scalar μβ product, where μ represents the permanent dipole moment of the chromophore and β corresponds to the vector component of the first hyperpolarizability tensor. Using this approach, the NLO properties of the selected cyano-rich chromophores were quantitatively assessed and compared with the corresponding theoretical predictions (Table 2).^9,59–61^ EFISHG measurements were carried out in chloroform using an incident beam at 1907 nm under nonresonant conditions. Sample solutions were prepared at concentrations between 10^–2^ and 10^–3^ M, and the NLO responses were obtained using a Raman-shifted Nd:YAG laser (λ = 1907 nm).

**Table 2:** Calculated and Measured μβ Values of Chromophores **12a**, **12c**, **12e**, and **13**

compound	μ^a^ (Debye)	β^a^ (10^–30^ esu)	μβ (10^–48^ esu^2^·cm)^b^	μβ (10^–48^ esu)^c^
**12a**	12.8237	117	1500	300
**12c**	10.1168	91	921	260
**12e**	11.3467	60	681	150
**13**	17.7664	153	2718	560

a Calculated at the DFT CAM-B3LYP/6–31G++(d,p) level in CHCl_3_.

b 1D = 1 × 10^–18^ esu·cm.

c μβ (2ω) at 1907 nm in CHCl_3_, molecular concentrations used for the measurements were in the range of 10^–3^ to 10^–2^ M, μβ ± 10%.

The calculated NLO responses generally overestimate the experimentally obtained values. Although the computed hyperpolarizabilities are approximately 4–5 times larger than the EFISHG results, the overall trend is consistently preserved across all chromophores. Among the studied compounds, chromophore **13** exhibits the highest experimental μβ value, measured as 560 × 10^–48^ esu, which is in good agreement with literature-reported data for TCBD-based systems evaluated by the same technique. While this enhancement cannot be rationalized by the Δ*E* ^TD^ values when compared to chromophores **12a**, **12c**, and **12e**, the observation is consistent with a permanent dipole moment of **13** (μ = 17.7664 D) within the series. Within the series of tricyanovinylation products **12a**, **12c**, and **12e**, the differences in the experimental μβ values are relatively small, and therefore no clear trend can be distinguished. Nevertheless, the calculated μβ values increase systematically with the dipole moment, indicating that the efficiency of intramolecular charge separation plays a predominant role in governing the NLO response. Taken together, these results demonstrate that the enhanced NLO activity observed for chromophores **12a**, **12c**, **12e**, and **13** arises primarily from their pronounced donor–acceptor polarization and large dipole moments rather than from variations in their electronic excitation energies. The good agreement between the calculated μβ values and the experimental μβ results further confirms that the magnitude of β itself plays a significant role in determining the overall NLO response. The obtained results are consistent with the benchmark EFISHG chromophore Disperse Red 1 (500 × 10^–48^ esu).^30^ Recent studies on hydrazone-substituted TCBD systems have reported experimental μβ values in the range of 520–1000 × 10^–48^ esu,^9^ whereas carbamate-containing TCBD derivatives exhibited μβ values of 470–670 × 10^–48^ esu.^50^ Based on these comparisons, chromophore **13** demonstrates NLO properties in good agreement with the literature, while the tricyanovinylation products **12a**, **12c**, and **12e** show comparatively weaker NLO responses even relative to Disperse Red 1.

## Conclusion

Within the scope of this study, ten distinct push–pull chromophores were synthesized through two different synthetic routes arising from the competitive reactivity of the C-2 and C-3 positions of the indole core. To enable the preparation of these chromophores, ten alkyne derivatives were designed and successfully obtained via Sonogashira cross-coupling reactions, providing isolated yields ranging from 41% to 91%. Reactions of the alkyne-containing substrates with TCNE **10** revealed that the high electron density at the C-3 position in the presence of ZnCl_2_ favors tricyanovinylation, whereas activation of the alkynes at the C-2 position by donor substituents promotes the [2 + 2] CA–RE reaction. Using this approach, eight different tricyanovinyl-substituted chromophores were successfully obtained in 40–82% yields, along with two [2 + 2] CA–RE products isolated in 30–63% yields. All synthesized chromophores absorb light in the visible region and display ICT bands with λ_max_ values ranging from 471 to 504 nm and molar absorptivities of ε *=* 14150–29100 M^–1^ cm^–1^. The ICT bands of the chromophores were experimentally confirmed through both solvatochromism studies and protonation experiments. Both the characteristic features of the ICT bands of the chromophores and the NLO properties of the synthesized systems were comprehensively investigated with the aid of computational chemistry. FMO analyses and ESP surface mappings provided detailed insight into the electron distributions, revealing that the LUMO is predominantly localized on the tricyanovinyl moiety, whereas in chromophore **13** it is mainly concentrated on the TCBD unit. Consistently, the calculated average polarizability and first hyperpolarizability values indicate that chromophore **13** is expected to exhibit the highest NLO performance among the synthesized compounds. In addition to theoretical studies, EFISHG measurements were conducted on four chromophores to evaluate their NLO properties. The general trend observed in both computational and experimental studies is in agreement, and the highest experimental μβ value (560 × 10^–48^ esu) corresponds to product **13**, demonstrating its strong potential for NLO applications.

## Experimental Section

### General

Commercially available chemicals were purchased and no further purification steps were undertaken. Compounds **2**,^62^ **3**,^63^ **5**,^64^ **6**,^64^ **8c**,^65^ and **8e** ^66^ were synthesized following slightly modified procedures reported in the literature. Solvents (such as dichloromethane, hexanes, and ethyl acetate) utilized for extraction or column chromatography were distilled prior to use. Sonogashira cross-coupling reactions were conducted in a nitrogen atmosphere using dry glassware. Column chromatography (CC) using SiO_2_-60 mesh was employed to purify the target compounds. Analytical thin-layer chromatography (TLC) was performed on aluminum sheets coated with 0.2 mm silica gel 60 F254, and visualization was achieved using a UV lamp (254 or 366 nm). The solvents were evaporated under vacuum at temperatures ranging from 25 to 60 °C and pressures between 900 and 10 mbar. ^1^H and ^13^C­{^1^H} nuclear magnetic resonance (NMR) spectra were acquired at frequencies of 400 MHz for ^1^H and 100 MHz for ^13^C­{^1^H}, respectively. Chemical shifts (δ) are expressed in parts per million (ppm) relative to tetramethylsilane (TMS), utilizing the residual deuterated solvent signal as an internal reference (CDCl_3_: δ_H_ = 7.26 ppm, δ_C_ = 77.0 ppm). In ^1^H NMR spectroscopy, resonance multiplicity is denoted as s (singlet), d (doublet), t (triplet), q (quartet), quint (quintet), sext (sextet), sept (septet), m (multiplet), and br. (broad). Coupling constants (*J*) are provided in hertz (Hz). Additionally, all spectra were acquired at room temperature. High-resolution mass spectrometry (HRMS) analysis was conducted by the mass spectrometry service at the Central Laboratory of Middle East Technical University, Turkey. Masses are presented in units of mass to-charge ratio (*m*/*z*) as the molecular ion, represented as [M + H]^+^. UV/vis spectra were obtained using a T80+ UV/vis spectrophotometer. Measurements were taken in a 1 cm quartz cuvette at 298 K. The absorption maxima (λ_max_) are given in nm, with the extinction coefficient (ε) in M^–1^ cm^–1^ provided in parentheses. A representative sample from each of the two different chromophore groups was dissolved in CH_2_Cl_2_ at concentrations ranging from 1 × 10^–5^ to 3.2 × 10^–5^ M to verify compliance with the Beer–Lambert law. After confirmation, UV/vis measurements for all chromophores were conducted in CH_2_Cl_2_ at a concentration of 2 × 10^–5^ M.

#### Synthesis of Compound **3**

Compound **2** (607 mg, 4.63 mmol, 1 equiv) was dissolved in Et_2_O (15 mL), cooled to 0 °C, and stirred for 30 min under an inert nitrogen atmosphere. Upon addition of *n*-BuLi (2.78 mL, 6.94 mmol, 1.5 equiv), the mixture was stirred for an additional 30 min and then heated to reflux in an oil bath for 3 h. Subsequently, the reaction was cooled to 15 °C and iodine (1.29 g, 5.09 mmol, 1.10 equiv) was added. After 1 h, the mixture was quenched with water containing 20% Na_2_SO_3_, and the aqueous phase was extracted with EtOAc (3 × 30 mL), drying over MgSO_4_, and filtration. Removal of the solvent under reduced pressure yielded product **3**, which was purified by column chromatography (CC) (SiO_2_; 9:1 hexanes/EtOAc). Yield: 635 mg; yellow oil; 53%; *R*_f_ = 0.61 (SiO_2_; 9:1 hexanes/EtOAc); ^1^H NMR (400 MHz, CDCl_3_, 298 K); δ = 7.53 (dd, *J* = 7.9, 0.8 Hz, 1H), 7.31 (d, *J* = 8.2 Hz, 1H), 7.19–7.14 (m, 1H), 7.11–7.05 (m, 1H), 6.81 (s, 1H), 3.77 ppm (s, 3H); ^13^C {^1^H} NMR (100 MHz, CDCl_3_, 298 K); δ = 138.1, 129.7, 121.9, 119.9, 119.6, 111.9, 109.8, 84.0, 34.1 ppm; Spectral data is consistent with the literature.^57^

#### Synthesis of Compound **5**

Compound **3** (470 mg, 1.83 mmol, 1 equiv) was dissolved in 10 mL of triethylamine in a flask sealed with a rubber septum and the atmosphere was purged with N_2_ for 30 min. Then, [Pd­(PPh_3_)_2_Cl_2_] (39 mg, 0.05 mmol, 0.03 equiv) and CuI (11 mg, 0.05 mmol, 0.03 equiv) were added to the solution and stirred for an additional 30 min. Subsequently, trimethylsilylacetylene (0.28 mL, 2.01 mmol, 1.1 equiv) was added, and the mixture was stirred overnight at room temperature. The solvent was removed under reduced pressure, and the resulting solid was extracted with CH_2_Cl_2_ (3 × 30 mL). The organic phase was dried over MgSO_4_, and the solvent was evaporated. Purification by CC (SiO_2_; 9:1 hexanes/EtOAc) yielded the desired product **5**. Yield: 388 mg; yellow solid; 93%; R_*f*_ = 0.68 (SiO_2_; 9:1 hexanes/EtOAc); ^1^H NMR (400 MHz, CDCl_3_, 298 K); δ = 7.57 (d, *J* = 7.9 Hz, 1H), 7.28–7.23 (m, 2H), 7.13–7.08 (m, 1H), 6.77 (s, 1H), 3.79 (s, 3H), 0.30 ppm (s, 9H); ^13^C {^1^H} NMR (100 MHz, CDCl_3_, 298 K); δ = 137.3, 127.2, 123.3, 122.1, 121.2, 120.2, 109.6, 108.0, 101.3, 96.5, 30.6, 0.1 ppm; Spectral data is consistent with the literature.^64^

#### Synthesis of Compound **6**

Compound **5** (702 mg, 3.09 mmol, 1 equiv) was dissolved in 15 mL of methanol, followed by the addition of K_2_CO_3_ (1.41 g, 10.19 mmol, 3.3 equiv) to the solution. Following filtration with 9:1 hexanes/EtOAc, evaporation, and CC (SiO_2_; 9:1 hexanes/EtOAc), alkyne **6** was isolated. Yield: 438 mg; yellow oil; 91%; *R*_f_ = 0.57 (SiO_2_; 9:1 hexanes/EtOAc); ^1^H NMR (400 MHz, CDCl_3_, 298 K); δ = 7.60 (d, *J* = 8.0 Hz, 1H), 7.32–7.27 (m, 2H), 7.16–7.11 (m, 1H), 6.84 (s, 1H), 3.83 (s, 3H), 3.49 ppm (s, 1H); ^13^C {^1^H} NMR (100 MHz, CDCl_3_, 298 K); δ = 137.3, 127.0, 123.4, 121.3, 121.0, 120.3, 109.6, 108.4, 83.5, 75.8, 30.7 ppm; Spectral data is consistent with the literature.^64^

#### Synthesis of Compound **8a**

The aryl iodide **7a** (160 mg, 0.64 mmol, 1 equiv) was dissolved in 3 mL of diisopropylamine (DIPA) and 6 mL of toluene and the atmosphere was purged with N_2_ for 30 min. Addition of [Pd­(PPh_3_)_2_Cl_2_] (41 mg, 0.058 mmol, 0.09 equiv) and CuI (11 mg, 0.058 mmol, 0.09 equiv) was followed by a further 30 min N_2_ purge. Alkyne **6** (120 mg, 0.77 mmol, 1.2 equiv) was then added to the reaction medium. The reaction was heated to 60 °C in an oil bath and stirred overnight. The mixture was extracted with CH_2_Cl_2_ (3 × 30 mL) and purified by CC (SiO_2_; 9:1 hexanes/EtOAc). Yield: 130 mg; yellow liquid, 73%; R_*f*_ = 0.37 (SiO_2_; 9:1 hexanes/EtOAc); m.p. = 201–203 °C; ^1^H NMR (400 MHz, CDCl_3_, 298 K); δ = 8.24 (quasi d, *J* = 8.6 Hz, 2H), 7.69 (quasi d, *J* = 8.6 Hz, 2H), 7.63 (d, *J* = 8.0 Hz, 1H), 7.34–7.30 (m, 2H), 7.18–7.13 (m, 1H), 6.94 (s, 1H), 3.89 ppm (s, 3H); ^13^C {^1^H} NMR (100 MHz, CDCl_3_, 298 K); δ = 147.2, 137.9, 132.0, 129.8, 127.3, 124.0, 123.9, 121.5, 120.9, 120.6, 109.7, 109.4, 93.8, 86.8, 30.9 ppm; IR (ATR): ν̃ = 2209 (m), 1592 (s), 1516 (s), 1285 (s) cm^–1^; HRMS (ESI-TOF) m/*z*: calcd. for C_17_H_13_N_2_O_2_^+^: 277.0977; found: 277.0980 [M + H]^+^.

#### General Procedure for the Synthesis of Compounds **8b**–**d**

The aryl iodide **7b** (1 equiv) was dissolved in 3 mL of DIPA and 6 mL of toluene, whereas **7c** and **7d** (1 equiv) were dissolved in 6 mL of triethylamine and stirred for 30 min under an inert N_2_ atmosphere. Subsequently, [Pd­(PPh_3_)_2_Cl_2_] (0.03 equiv) and CuI (0.03 equiv) were added to the solution, and the reaction mixture was degassed with N_2_ for an additional 30 min. Upon addition of the alkyne **6** (1.2 equiv), **8b** was heated to 60 °C in an oil bath, whereas **8c** and **8d** were stirred at room temperature overnight. The mixture was extracted with CH_2_Cl_2_ (3 × 30 mL). The combined organic phase was dried over MgSO_4_, and the solvent was evaporated. Purification by CC (SiO_2_; 9:1 hexanes/EtOAc) yielded the coupling products **8b**–**d** in 66–74% yields.

#### Compound **8b**

(Starting material **6**: 200 mg, 1.29 mmol); yield: 185 mg; orange-yellow solid, 70%; *R*_f_ = 0.50 (SiO_2_; 9:1 hexanes/EtOAc); m.p. = 138–140 °C; ^1^H NMR (400 MHz, CDCl_3_, 298 K); δ = 7.58 (dd, *J* = 8.0, 0.8 Hz, 1H), 7.45 (quasi d, *J* = 7.7 Hz, 2H), 7.27–7.21 (m, 2H), 7.16 (quasi d, *J* = 7.7 Hz, 2H), 7.13–7.08 (m, 1H), 6.80 (s, 1H), 3.84 (s, 3H), 2.36 ppm (s, 3H); ^13^C {^1^H} NMR (100 MHz, CDCl_3_, 298 K); δ = 139.0, 137.4, 131.5, 129.4, 127.5, 123.0, 122.4, 121.0, 120.2, 119.8, 109.5, 107.3, 95.5, 80.6, 30.8, 21.7 ppm; IR (ATR): ν̃ = 3055 (w), 1500 (w), 1458 (m), 1343 (s) cm^–1^; HRMS (ESI-TOF) m/*z*: calcd. for C_18_H_16_N^+^: 246.1283; found: 246.1285 [M + H]^+^.

#### Compound **8c**

(Starting material **6**: 200 mg, 1.29 mmol); yield: 208 mg; red-orange liquid, 74%; *R*_f_ = 0.58 (SiO_2_; 9:1 hexanes/EtOAc); m.p. = 134–136 °C; ^1^H NMR (400 MHz, CDCl_3_, 298 K); δ = 7.60 (d, *J* = 7.9 Hz, 1H), 7.52 (quasi d, *J* = 8.8 Hz, 2H), 7.32–7.27 (m, 2H), 7.15–7.10 (m, 1H), 6.91 (quasi d, *J* = 8.8 Hz, 2H), 6.80 (s, 1H), 3.87 (s, 3H), 3.85 ppm (s, 3H); ^13^C {^1^H} NMR (100 MHz, CDCl_3_, 298 K); δ = 160.0, 137.4, 133.1, 127.5, 122.9, 122.6, 121.0, 120.1, 114.9, 114.3, 109.5, 107.0, 95.3, 79.9, 55.5, 30.8 ppm. Spectral data consistent with the literature.^65^

#### Compound **8d**

(Starting material **6**: 200 mg, 1.29 mmol); yield: 215 mg; yellow solid, 66%; *R*_f_ = 0.53 (SiO_2_; 9:1 hexanes/EtOAc); m.p. = 166–168 °C; ^1^H NMR (400 MHz, CDCl_3_, 298 K); δ = 7.87–7.82 (m, 1H), 7.71 (quasi d, *J* = 8.5 Hz, 2H), 7.59–7.50 (m, 2H), 7.42–7.37 (m, 1H), 7.05 (s, 1H), 6.92 (quasi d, *J* = 8.5 Hz, 2H), 4.14 (s, 3H), 3.67 (q, *J* = 7.0 Hz, 4H), 1.47 ppm (t, *J* = 7.0 Hz, 6H); ^13^C {^1^H} NMR (100 MHz, CDCl_3_, 298 K); δ = 147.9, 137.3, 133.1, 127.6, 123.4, 122.5, 120.8, 119.9, 111.3, 109.4, 108.2, 106.2, 96.7, 78.7, 44.5, 30.7, 12.7 ppm; IR (ATR): ν̃ = 2971 (w), 2194 (w), 1602 (m), 1164 (s), 814 (s) cm^–1^; HRMS (ESI-TOF) m/*z*: calcd. for C_21_H_23_N_2_^+^: 303.1861; found: 303.1861 [M + H]^+^.

#### General Procedure for the Synthesis of Compounds **8e**–**i**

Iodoindole **3** (1 equiv) was dissolved in a mixture of 3 mL of DIPA and 6 mL of toluene (for compounds **8f**–**i**) or in 6 mL of triethylamine (for compound **8e**), and the mixture was purged with nitrogen for 30 min. [Pd­(PPh_3_)_2_Cl_2_] (0.09 equiv) and CuI (0.09 equiv) were added to the medium and purged for an additional 30 min. Terminal alkynes **9e**–**i** (2 equiv) were added to the reaction mixture. Compound **8e** was stirred overnight at room temperature in the presence of triethylamine, while compounds **8f**–**i** were heated to 60 °C in a toluene/DIPA mixtures in an oil bath. The mixture was extracted with CH_2_Cl_2_ (3 × 30 mL), the combined organic layer was dried over MgSO_4_, and the solvent was evaporated. Column chromatography was carried out to purify and isolate the coupling products **8e**–**i** (41–78%).

#### Compound **8e**

(Starting material **3**: 250 mg, 0.97 mmol); yield: 171 mg; orange-yellow solid, 76%; CC: (SiO_2_; 10:1 hexanes/EtOAc); *R*_f_ = 0.52 (SiO_2_; 9:1 hexanes/EtOAc); ^1^H NMR (400 MHz, CDCl_3_, 298 K); δ = 7.63–7.57 (m, 3H), 7.41–7.36 (m, 3H), 7.32–7.26 (m, 2H), 7.14 (t, *J* = 7.2 Hz, 1H), 6.85 (s, 1H), 3.88 ppm (s, 3H); ^13^C {^1^H} NMR (100 MHz, CDCl_3_, 298 K); δ = 137.4, 131.6, 128.7, 128.6, 127.4, 123.1, 122.8, 122.2, 121.1, 120.2, 109.5, 107.5, 95.3, 81.2, 30.8 ppm. Spectral data was consistent with the literature.^66^

#### Compound **8f**

(Starting material **3**: 350 mg, 1.36 mmol); yield: 273 mg; yellow solid, 71%; CC: (SiO_2_; 9:1 hexanes/CH_2_Cl_2_); *R*_f_ = 0.41 (SiO_2_; 9:1 hexanes/EtOAc); m.p. = 200–202 °C; ^1^H NMR (400 MHz, CDCl_3_, 298 K); δ = 8.10 (s, 1H), 7.87–7.83 (m, 3H), 7.65–7.61 (m, 2H), 7.55–7.51 (m, 2H), 7.34–7.27 (m, 2H), 7.15 (t, *J* = 7.2 Hz, 1H), 6.89 (s, 1H), 3.92 ppm (s, 3H); ^13^C {^1^H} NMR (100 MHz, CDCl_3_, 298 K); δ = 137.5, 133.2, 133.1, 131.5, 128.3, 128.2, 127.98, 127.95, 127.5, 127.0, 126.9, 123.2, 122.2, 121.1, 120.24, 120.16, 109.6, 107.7, 95.8, 81.6, 30.9 ppm; IR (ATR): ν̃ = 2200 (m), 1597 (s), 1519 (s), 1188 (m), 813 (s) cm^–1^; HRMS (ESI-TOF) m/*z*: calcd. for C_21_H_16_N^+^: 282.1283; found: 282.1285 [M + H]^+^.

#### Compound **8g**

(Starting material **3**: 340 mg, 1.32 mmol); yield: 257 mg; yellow solid, 69%; CC: (SiO_2_; 9:1 hexanes/CH_2_Cl_2_); *R*_f_ = 0.48 (SiO_2_; 9:1 hexanes/EtOAc); m.p. = 111–113 °C; ^1^H NMR (400 MHz, CDCl_3_, 298 K); δ = 8.45 (d, *J* = 8.3 Hz, 1H), 7.93–7.86 (m, 2H), 7.85–7.79 (m, 1H), 7.68–7.61 (m, 2H), 7.60–7.54 (m, 1H), 7.53–7.46 (m, 1H), 7.38–7.28 (m, 2H), 7.20–7.12 (m, 1H), 6.97 (s, 1H), 3.99 ppm (s, 3H); ^13^C {^1^H} NMR (100 MHz, CDCl_3_, 298 K); δ = 137.6, 133.4, 133.2, 130.6, 129.2, 128.6, 127.5, 127.1, 126.7, 126.2, 125.5, 123.3, 122.3, 121.2, 120.6, 120.3, 109.6, 107.9, 93.6, 86.1, 31.0 ppm; IR (ATR): ν̃ = 1462 (w), 1342 (m), 800 (s) cm^–1^; HRMS (ESI-TOF) m/*z*: calcd. for C_21_H_16_N^+^: 282.1283; found: 282.1283 [M + H]^+^.

#### Compound **8h**

(Starting material **3**: 120 mg, 0.47 mmol); yield: 120 mg; yellow solid, 78%; CC: (SiO_2_; 9:1 hexanes/CH_2_Cl_2_); *R*_f_ = 0.54 (SiO_2_; 9:1 hexanes/EtOAc); m.p. = 176–178 °C; ^1^H NMR (400 MHz, CDCl_3_, 298 K); δ = 8.76–8.72 (m, 1H), 8.69 (d, *J* = 8.5 Hz, 1H), 8.56–8.52 (m, 1H), 8.14 (s, 1H), 7.90 (d, *J* = 8.1 Hz, 1H), 7.77–7.70 (m, 3H), 7.69–7.61 (m, 2H), 7.38–7.29 (m, 2H), 7.16 (t, *J* = 7.3 Hz, 1H), 6.98 (s, 1H), 4.01 ppm (s, 3H); ^13^C {^1^H} NMR (100 MHz, CDCl_3_, 298 K); δ = 137.3, 131.9, 131.1, 130.7, 130.3, 130.1, 128.5, 127.6, 127.2, 127.12, 127.11, 127.0, 126.7, 123.1, 122.8, 122.6, 122.0, 120.9, 120.1, 119.0, 109.4, 107.7, 93.5, 85.4, 30.8 ppm; IR (ATR): ν̃ = 1342 (w), 749 (s), 733 (s) cm^–1^; HRMS (ESI-TOF) m/*z*: calcd. for C_25_H_18_N^+^: 332.1439; found: 332.1437 [M + H]^+^.

#### Compound **8i**

(Starting material **3**: 216 mg, 0.84 mmol); yield: 107 mg; yellow solid, 41%; CC: (SiO_2_; 9:1 hexanes/CH_2_Cl_2_); *R*_f_ = 0.48 (SiO_2_; 9:1 hexanes/EtOAc); m.p. = 204–206 °C; ^1^H NMR (400 MHz, CDCl_3_, 298 K); δ = 7.66–7.59 (m, 7H), 7.47 (t, *J* = 7.5 Hz, 2H), 7.41–7.35 (m, 1H), 7.33–7.27 (m, 2H), 7.16–7.11 (m, 1H), 6.86 (s, 1H), 3.90 ppm (s, 3H); ^13^C {^1^H} NMR (100 MHz, CDCl_3_, 298 K); δ = 141.5, 140.4, 137.6, 132.0, 129.1, 127.9, 127.5, 127.3, 127.2, 123.2, 122.3, 121.8, 121.1, 120.3, 109.6, 107.6, 95.3, 82.0, 30.8 ppm; IR (ATR): ν̃ = 1456 (w), 1379 (w), 1344 (m), 837 (s) cm^–1^; HRMS (ESI-TOF) m/*z*: calcd. for C_23_H_18_N^+^: 308.1439; found: 308.1432 [M + H]^+^.

#### Synthesis of Compound **12e** with SiO_2_

SiO_2_ (10 g) was added to a solution of indole-substituted-alkyne **8e** (50 mg, 0.22 mmol, 1 equiv) and TCNE (33 mg, 0.26 mmol, 1.2 equiv) in CH_2_Cl_2_ (20 mL) and the mixture was stirred at 25 °C. After 24 h, the crude mixture was filtered and washed with EtOAc. Evaporation and CC (SiO_2_; CH_2_Cl_2_) gave the target product **12e**. Yield: 11 mg; brick red solid, 16%.

#### General Procedure for the Synthesis of Compounds **12a**–**c** and **12e**–**i**

Coupling products **8a**–**c** and **8e**–**i** (1 equiv) were dissolved in acetonitrile, followed by the addition of TCNE (1.2 equiv). After stirring at room temperature for 10 min, ZnCl_2_ (2 equiv) was added, and the mixture was refluxed overnight. Evaporation and CC (SiO_2_; CH_2_Cl_2_) gave the target products **12a**–**c** and **12e**–**i** in 40–82% yields.

#### Compound **12a**

(Starting material **8a**: 50 mg, 0.18 mmol); yield: 42 mg; brick red solid; 62%; CC: (SiO_2_; CH_2_Cl_2_); *R*_f_ = 0.65 (SiO_2_; CH_2_Cl_2_); m.p. = 222–224 °C; ^1^H NMR (400 MHz, CDCl_3_, 298 K); δ = 8.32 (d, *J* = 8.4 Hz, 2H), 7.90 (d, *J* = 8.9 Hz, 3H), 7.57–7.52 (m, 1H), 7.51–7.46 (m, 2H), 4.05 ppm (s, 3H); ^13^C {^1^H} NMR (100 MHz, CDCl_3_, 298 K); δ = 148.6, 138.0, 133.3, 133.1, 127.3, 127.09, 127.07, 124.8, 124.2, 124.0, 121.5, 113.7, 112.6, 112.2, 111.3, 105.4, 87.4, 82.3, 32.5 ppm (19 out of 20 signals expected); UV/vis (CH_2_Cl_2_): λ_max_(ε) = 359 (1.95 × 10^–4^), 492 nm (1.41 × 10^–4^ M^–1^cm^–1^); IR (ATR): ν̃ = 3104 (w), 2231 (s), 2220 (s), 1593 (m), 1289 (s) cm^–1^; HRMS (ESI-TOF) m/*z*: calcd. for C_22_H_12_N_5_O_2_^+^: 378.0991; found: 378.0991 [M + H]^+^.

#### Compound **12b**

(Starting material **8b**: 50 mg, 0.20 mmol); yield: 46 mg; brick red solid; 65%; CC: (SiO_2_; CH_2_Cl_2_); *R*_f_ = 0.64 (SiO_2_; CH_2_Cl_2_). m.p. = 252–254 °C; ^1^H NMR (400 MHz, CDCl_3_, 298 K); δ = 7.89 (d, *J* = 7.7 Hz, 1H), 7.62 (quasi d, *J* = 8.1 Hz, 2H), 7.50–7.41 (m, 3H), 7.25 (quasi d, *J* = 8.1 Hz, 2H), 4.01 (s, 3H), 2.43 ppm (s, 3H); ^13^C {^1^H} NMR (100 MHz, CDCl_3_, 298 K); δ = 141.7, 137.9, 133.1, 132.3, 129.8, 129.6, 126.5, 124.4, 124.1, 121.3, 117.5, 113.9, 113.0, 112.8, 111.5, 111.1, 109.7, 85.8, 78.4, 32.3, 21.9 ppm; UV/vis (CH_2_Cl_2_): λ_max_(ε) = 314 (3.62 × 10^–4^), 337 (2.65 × 10^–4^), 498 nm (1.56 × 10^–4^ M^–1^cm^–1^); IR (ATR): ν̃ = 2916 (s), 2850 (m), 2206 (s), 1729 (m), 1442 (s) cm^–1^; HRMS (ESI-TOF) m/*z*: calcd. for C_23_H_15_N_4_^+^: 347.1297; found: 347.1297 [M + H]^+^.

#### Compound **12c**

(Starting material **8c**: 50 mg, 0.19 mmol); yield: 57 mg; brick red solid; 82%; CC: (SiO_2_; CH_2_Cl_2_); *R*_f_ = 0.64 (SiO_2_; CH_2_Cl_2_); m.p. = 219–221 °C; ^1^H NMR (400 MHz, CDCl_3_, 298 K); δ = 7.88 (d, *J* = 8.3 Hz, 1H), 7.67 (quasi d, *J* = 8.9 Hz, 2H), 7.52–7.39 (m, 3H), 6.96 (quasi d, *J* = 8.9 Hz, 2H), 4.00 (s, 3H), 3.88 ppm (s, 3H); ^13^C {^1^H} NMR (100 MHz, CDCl_3_, 298 K); δ = 161.8, 137.9, 134.2, 133.0, 130.0, 126.4, 124.3, 124.0, 121.3, 114.7, 113.9, 113.1, 112.9, 112.4, 111.3, 111.1, 110.1, 85.1, 78.2, 55.7, 32.3 ppm; UV/vis (CH_2_Cl_2_): λ_max_(ε) = 317 (4.32 × 10^–4^), 342 (3.52 × 10^–4^), 504 nm (1.85 × 10^–4^ M^–1^cm^–1^); IR (ATR): ν̃ = 2217 (m), 2199 (s), 1602 (m), 1521 (s), 823 (s) cm^–1^; HRMS (ESI-TOF) m/*z*: calcd. for C_23_H_15_N_4_O^+^: 363.1246; found: 363.1249 [M + H]^+^.

#### Compound **12e**

(Starting material **8e**: 40 mg, 0.17 mmol); yield: 35 mg; brick red solid; 61%; CC: (SiO_2_; CH_2_Cl_2_); *R*_f_ = 0.67 (SiO_2_; CH_2_Cl_2_); m.p. = 230–232 °C; ^1^H NMR (400 MHz, CDCl_3_, 298 K); δ = 7.89 (d, *J* = 8.1 Hz, 1H), 7.73 (d, *J* = 7.5 Hz, 2H), 7.52–7.47 (m, 3H), 7.46–7.43 (m, 3H), 4.02 ppm (s, 3H); ^13^C {^1^H} NMR (100 MHz, CDCl_3_, 298 K); δ = 137.9, 133.2, 132.4, 130.9, 129.2, 129.0, 126.5, 124.4, 124.0, 121.3, 120.5, 113.8, 112.9, 112.8, 111.5, 111.1, 109.0, 86.0, 78.6, 32.3 ppm; UV/vis (CH_2_Cl_2_): λ_max_(ε) = 311 (4.00 × 10^–4^), 334 (2.60 × 10^–4^), 494 nm (1.79 × 10^–4^ M^–1^cm^–1^); IR (ATR): ν̃ = 2205 (s), 1500 (s), 1442 (s), 748 (s) cm^–1^; HRMS (ESI-TOF) m/*z*: calcd. for C_22_H_13_N_4_^+^: 333.1140; found: 333.1140 [M + H]^+^.

#### Compound **12f**

(Starting material **8f**: 29 mg, 0.10 mmol); yield: 27 mg; brick red solid; 68%; CC: (SiO_2_; CH_2_Cl_2_); *R*_f_ = 0.65 (SiO_2_; CH_2_Cl_2_); m.p. = 213–215 °C; ^1^H NMR (400 MHz, CDCl_3_, 298 K): δ = 8.28 (s, 1H), 7.93–7.86 (m, 4H), 7.72 (d, *J* = 7.9 Hz, 1H), 7.61–7.57 (m, 2H), 7.52–7.44 (m, 3H), 4.05 ppm (s, 3H); ^13^C {^1^H} NMR (100 MHz, CDCl_3_, 298 K); δ = 137.9, 134.1, 133.4, 133.2, 132.9, 129.3, 128.9, 128.5, 128.2, 128.1, 127.8, 127.3, 126.6, 124.4, 124.0, 121.3, 117.7, 113.9, 113.0, 112.8, 111.6, 111.2, 109.6, 85.9, 79.0, 32.4 ppm; UV/vis (CH_2_Cl_2_): λ_max_(ε) = 331 (3.43 × 10^–4^), 346 (3.39 × 10^–4^), 501 nm (1.68 × 10^–4^ M^–1^cm^–1^); IR (ATR): ν̃ = 3045 (w), 2218 (m), 2187 (s), 1624 (w), 1454 (s) cm^–1^; HRMS (ESI-TOF) m/*z*: calcd. for C_26_H_15_N_4_^+^: 383.1297; found: 383.1320 [M + H]^+^.

#### Compound **12g**

(Starting material **8g**: 50 mg, 0.18 mmol); yield: 27 mg; brick red solid; 40%; CC: (SiO_2_; CH_2_Cl_2_); *R*_f_ = 0.69 (SiO_2_; CH_2_Cl_2_); m.p. = 196–198 °C; ^1^H NMR (400 MHz, CDCl_3_, 298 K); δ = 8.34 (d, *J* = 8.3 Hz, 1H), 8.06 (d, *J* = 7.1 Hz, 1H), 8.01 (d, *J* = 8.3 Hz, 1H), 7.96–7.89 (m, 2H), 7.69 (t, *J* = 7.7 Hz, 1H), 7.63–7.54 (m, 2H), 7.53–7.45 (m, 3H), 4.13 ppm (s, 3H); ^13^C {^1^H} NMR (100 MHz, CDCl_3_, 298 K); δ = 138.0, 133.3, 133.2, 133.0, 132.9, 131.6, 129.3, 129.0, 128.0, 127.2, 126.6, 125.7, 125.5, 124.5, 124.1, 121.3, 118.1, 113.9, 112.8, 111.6, 111.2, 107.4, 86.3, 83.1, 32.5 ppm (25 out of 26 signals expected); UV/vis (CH_2_Cl_2_): λ_max_(ε) = 330 (2.84 × 10^–4^), 358 (2.47 × 10^–4^), 501 nm (1.62 × 10^–4^ M^–1^cm^–1^); IR (ATR): ν̃ = 3058 (w), 2214 (m), 2195 (s), 1378 (s) cm^–1^; HRMS (ESI-TOF) m/*z*: calcd. for C_26_H_15_N_4_^+^: 383.1297; found: 383.1294 [M + H]^+^.

#### Compound **12h**

(Starting material **8h**: 75 mg, 0.23 mmol); yield: 67 mg; brick red solid; 69%; CC: (SiO_2_; CH_2_Cl_2_); *R*_f_ = 0.77 (SiO_2_; CH_2_Cl_2_); m.p. = 254–256 °C; ^1^H NMR (400 MHz, CDCl_3_, 298 K); δ = 8.78–8.74 (m, 1H), 8.70 (d, *J* = 8.2 Hz, 1H), 8.44–8.40 (m, 1H), 8.38 (s, 1H), 7.99 (d, *J* = 7.7 Hz, 1H), 7.92 (d, *J* = 7.2 Hz, 1H), 7.79–7.74 (m, 3H), 7.71–7.65 (m, 1H), 7.54–7.46 (m, 3H), 4.15 ppm (s, 3H); ^13^C {^1^H} NMR (100 MHz, CDCl_3_, 298 K); δ = 138.0, 135.2, 133.3, 131.4, 131.0, 130.4, 130.3, 129.6, 129.3, 129.1, 127.83, 127.77, 127.6, 126.6, 126.4, 124.5, 124.1, 123.4, 122.9, 121.3, 117.09, 117.07, 113.9, 112.9, 111.7, 111.2, 107.6, 82.7, 79.8, 32.6 ppm; UV/vis (CH_2_Cl_2_): λ_max_(ε) = 338 (3.64 × 10^–4^), 359 (3.42 × 10^–4^), 504 nm (1.72 × 10^–4^ M^–1^cm^–1^); IR (ATR): ν̃ = 2211 (m), 2192 (m), 1480 (s) cm^–1^; HRMS (ESI-TOF) m/*z*: calcd. for C_30_H_17_N_4_^+^: 433.1453; found: 433.1456 [M + H]^+^.

#### Compound **12i**

(Starting material **8i**: 40 mg, 0.13 mmol); yield: 29 mg; red solid; 55%; (SiO_2_; CH_2_Cl_2_); R_*f*_ = 0.19 (SiO_2_; CH_2_Cl_2_); m.p. = 183–185 °C; ^1^H NMR (400 MHz, CDCl_3_, 298 K); δ = 7.91–7.88 (m, 1H), 7.80 (quasi d, *J* = 8.6 Hz, 2H), 7.69 (quasi d, *J* = 8.6 Hz, 2H), 7.65–7.61 (m, 2H), 7.51–7.47 (m, 3H), 7.46–7.40 (m, 3H), 4.03 ppm (s, 3H); ^13^C {^1^H} NMR (100 MHz, CDCl_3_, 298 K); δ = 142.9, 139.1, 137.1, 132.3, 132.0, 128.5, 128.3, 127.5, 126.8, 126.5, 125.7, 123.6, 123.2, 120.5, 118.4, 113.0, 112.1, 112.0, 110.7, 110.3, 108.3, 85.1, 78.5, 31.5 ppm; UV/vis (CH_2_Cl_2_): λ_max_(*ε*) = 346 (3.01 × 10^–4^), 500 nm (1.36 × 10^–4^ M^–1^ cm^–1^); IR (ATR): ν̃ = 2208 (m), 1280 (s), 1253 (s) cm^–1^; HRMS (ESI-TOF) m/*z*: calcd. for C_28_H_17_N_4_^+^: 409.1453; found: 409.1456 [M + H]^+^.

#### General Procedure for the Synthesis of Compounds **13** and **14**

**6** and **8d** (1 equiv) were dissolved in 5 mL of CH_2_Cl_2_, and TCNE (1.2 equiv) was added to the solution. The reaction mixture was stirred overnight at room temperature. Following solvent evaporation and CC (SiO_2_; CH_2_Cl_2_), target chromophores **13** and **14** were obtained.

#### Compound **13**

(Starting material **8d**: 50 mg, 0.17 mmol); yield: 45 mg; brown solid; 63%; CC: (SiO_2_; CH_2_Cl_2_); *R*_f_ = 0.67 (SiO_2_; CH_2_Cl_2_); m.p. = 116–118 °C; ^1^H NMR (400 MHz, CDCl_3_, 298 K); δ = 7.84 (quasi d, *J* = 9.1 Hz, 2H), 7.64 (d, *J* = 8.1 Hz, 1H), 7.51–7.42 (m, 2H), 7.24–7.20 (m, 1H), 6.99 (s, 1H), 6.76 (quasi d, *J* = 9.1 Hz, 2H), 3.92 (s, 3H), 3.51 (q, *J* = 7.0 Hz, 4H), 1.28 ppm (t, *J* = 7.0 Hz, 6H); ^13^C {^1^H} NMR (100 MHz, CDCl_3_, 298 K); δ = 163.1, 158.5, 152.9, 143.5, 134.3, 133.3, 128.3, 127.6, 123.2, 122.6, 118.7, 115.8, 114.8, 114.2, 113.3, 112.3, 112.0, 111.7, 83.3, 74.7, 45.4, 34.8, 12.7 ppm; UV/vis (CH_2_Cl_2_): λ_max_ (ε) = 419 (3.91 × 10^–4^), 471 nm (2.91 × 10^–4^ M^–1^ cm^–1^); IR (ATR): ν̃ = 2918 (w), 2212 (s), 1599 (s), 1416 (s) cm^–1^; HRMS (ESI-TOF) m/*z*: calcd. for C_27_H_23_N_6_^+^: 431.1984; found: 431.1982 [M + H]^+^.

#### Compound **14**

(Starting material **6**: 56 mg, 0.36 mmol); yield: 31 mg; brown solid; 30%; CC: (SiO_2_; CH_2_Cl_2_); *R*_f_ = 0.43 (SiO_2_; CH_2_Cl_2_); m.p. = 161–163 °C; ^1^H NMR (400 MHz, CDCl_3_, 298 K); δ = 8.15 (s, 1H), 7.72­(d, *J* = 8.1 Hz, 1H), 7.52–7.44 (m, 1H), 7.43–7.38 (m, 1H), 7.30–7.22 (m, 1H), 7.11 (s, 1H), 3.69 ppm (s, 3H); ^13^C {^1^H} NMR (100 MHz, CDCl_3_, 298 K); δ = 153.2, 151.0, 141.7, 130.3, 128.0, 127.6, 123.4, 122.7, 114.0, 112.2, 111.7, 111.4, 111.0, 108.4, 98.6, 88.6, 33.0 ppm; UV/vis (CH_2_Cl_2_): λ_max_ (ε) = 495 nm (0.89 × 10^–4^ M^–1^ cm^–1^); IR (ATR): ν̃ = 2226 (s), 1161 (w), 1527 (s), 1348 (s) cm^–1^; HRMS (ESI-TOF) m/*z*: calcd. for C_17_H_8_N_5_^–^: 282.0780; found: 282.0780 [M – H]^−^.

## Supplementary Material



## Data Availability

The data underlying this study are available in the published article and its Supporting Information.

## Notes

Theoretical calculations, HRMS data, copies of UV/vis, ^1^H NMR and ^13^C­{^1^H} NMR spectra, IR spectra, X-ray data, TGA curves (PDF)
