# Supplementary material for: Substrate-Controlled Divergent Reactivity of 2‑Alkynylindoles: [2 + 2] Cycloaddition–Retroelectrocyclization versus Tricyanovinylation for the Synthesis of NLOphores
Source: J Org Chem. 2026 Jun 11;91(25):8497–510. doi: 10.1021/acs.joc.6c00004 (PMC13316991; doi:10.1021/acs.joc.6c00004)
Supplement: Supplementary file 1 [file jo6c00004_si_001.pdf]

Supporting Information

The Journal of Organic Chemistry

Substrate-Controlled Divergent Reactivity of 2-Alkynylindoles: [2+2] Cycloaddition-Retroelectrocyclization versus Tricyanovinylation for the Synthesis of NLOphores

Hazal Kayas,[a] Yagmur Unal,[a] Kubra Erden,[a] Alberto Barsella,[b] Onur Şahin,[c] Cagatay Dengiz[a,*]

[a] *Department of Chemistry, Middle East Technical University, 06800 Ankara, Turkey*

[b] *Département d'Optique Ultra-Rapide et Nanophotonique, IPCMS-CNRS, 23 Rue du Loess, BP 43, 67034, Strasbourg, Cedex 2, France*

[c] *Department of Occupational Health & Safety, Faculty of Health Sciences, Sinop University, Sinop 57000, Turkey*

Table of Contents

1. ^{1}H and ^{13}C NMR spectra S3
2. High-Resolution Mass Spectrometry (HR-MS) Data S25
3. IR Spectra S34
4. Theoretical Calculations S43
5. X-ray Diffraction Analysis S62
6. UV/Vis Studies S63
7. Thermal Gravimetric Analysis (TGA) Studies S65
8. Images S66
9. Proposed Mechanism S67
10. References S68

1. ^{1}H and ^{13}C NMR spectra

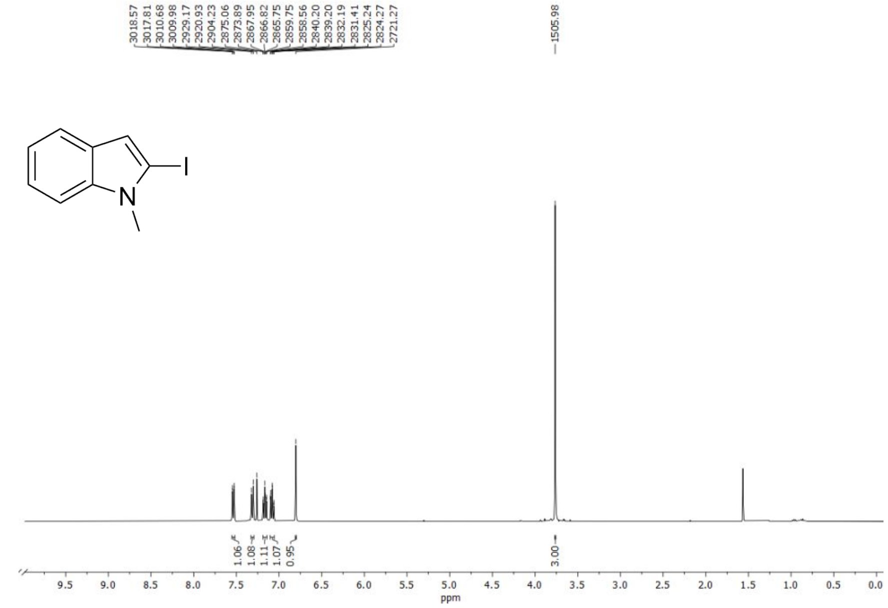

Figure S1. ^{1}H NMR spectrum of **3** in $CDCl_3$ solution (400 MHz).

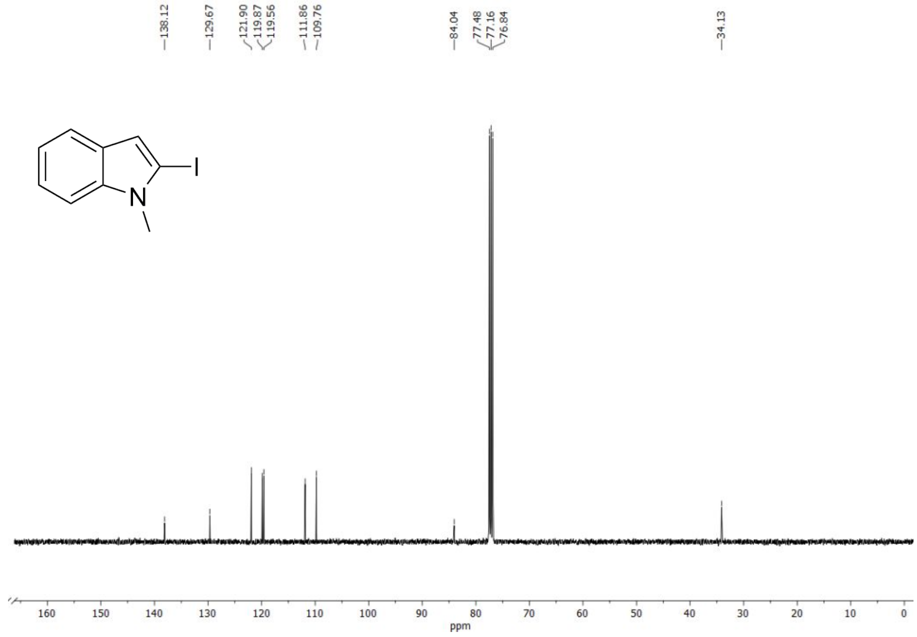

Figure S2. $^{13}C\{^{1}H\}$NMR spectrum of **3** in $CDCl_3$ solution (100 MHz).

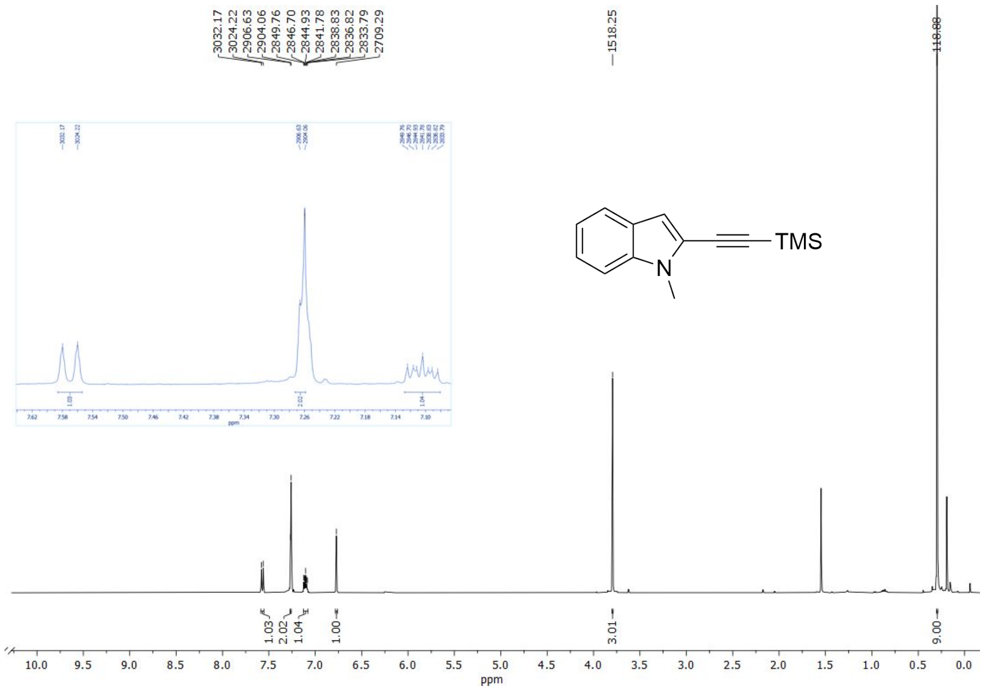

Figure S3. ^{1}H NMR spectrum of **5** in $CDCl_3$ solution (400 MHz).

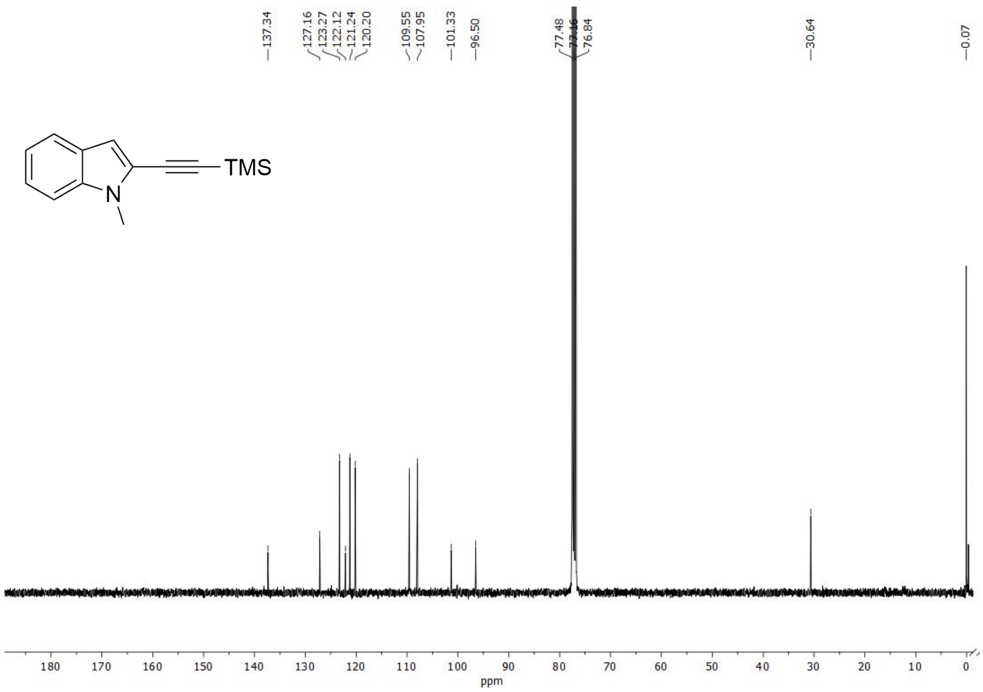

Figure S4. $^{13}C\{^{1}H\}$ NMR spectrum of **5** in $CDCl_3$ solution (100 MHz).

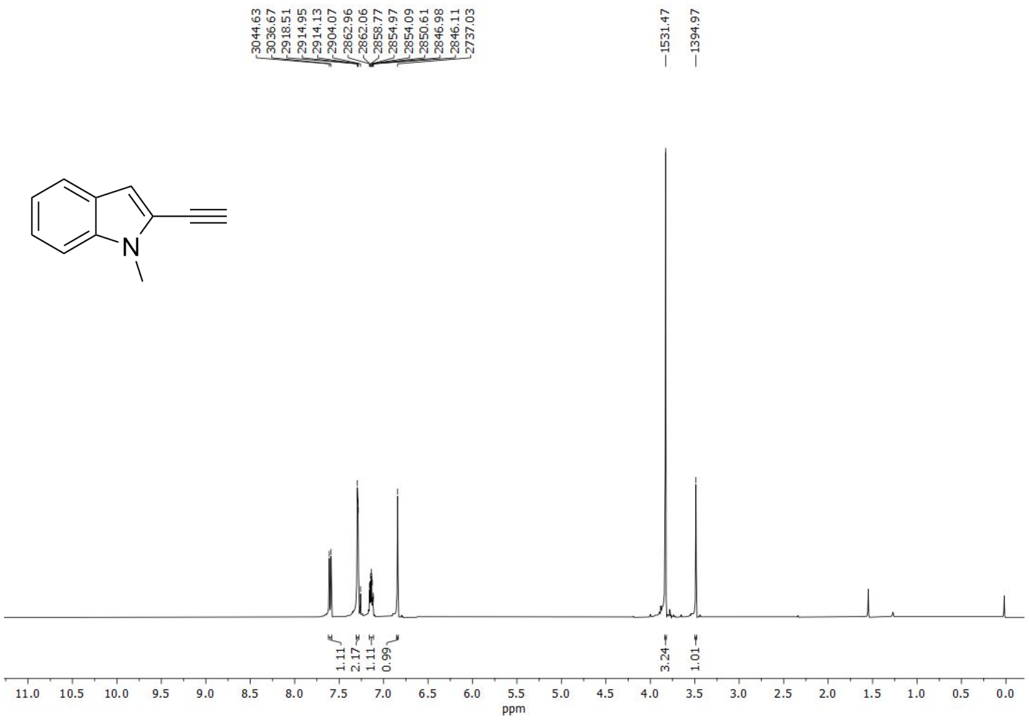

Figure S5. ^{1}H NMR spectrum of **6** in $CDCl_3$ solution (400 MHz).

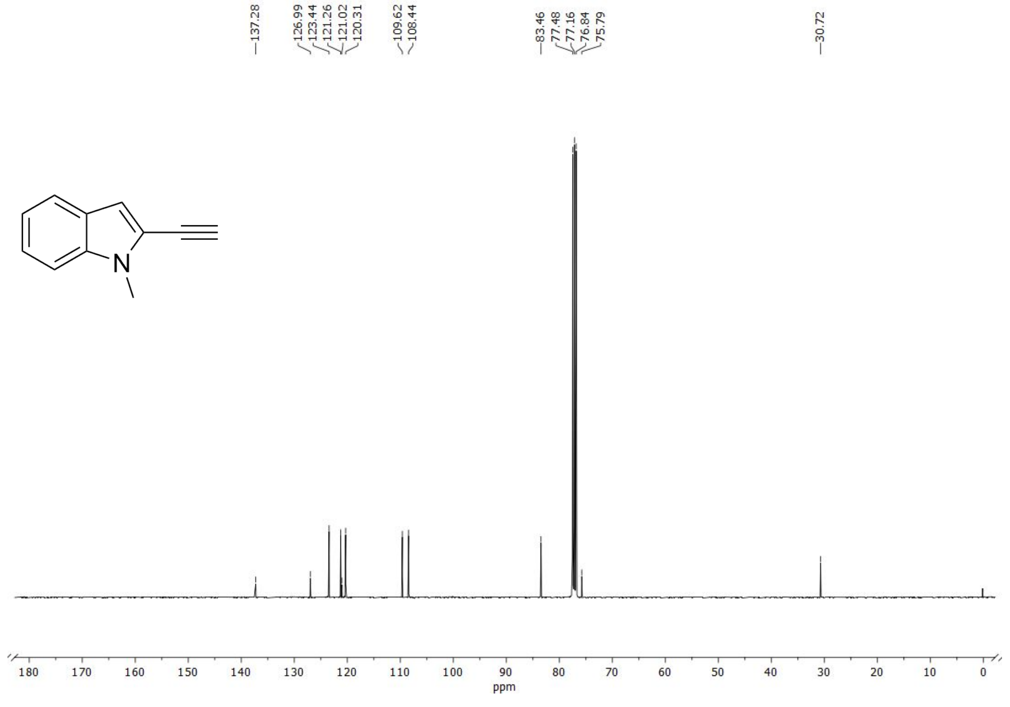

Figure S6. $^{13}C\{^{1}H\}$ NMR spectrum of **6** in $CDCl_3$ solution (100 MHz).

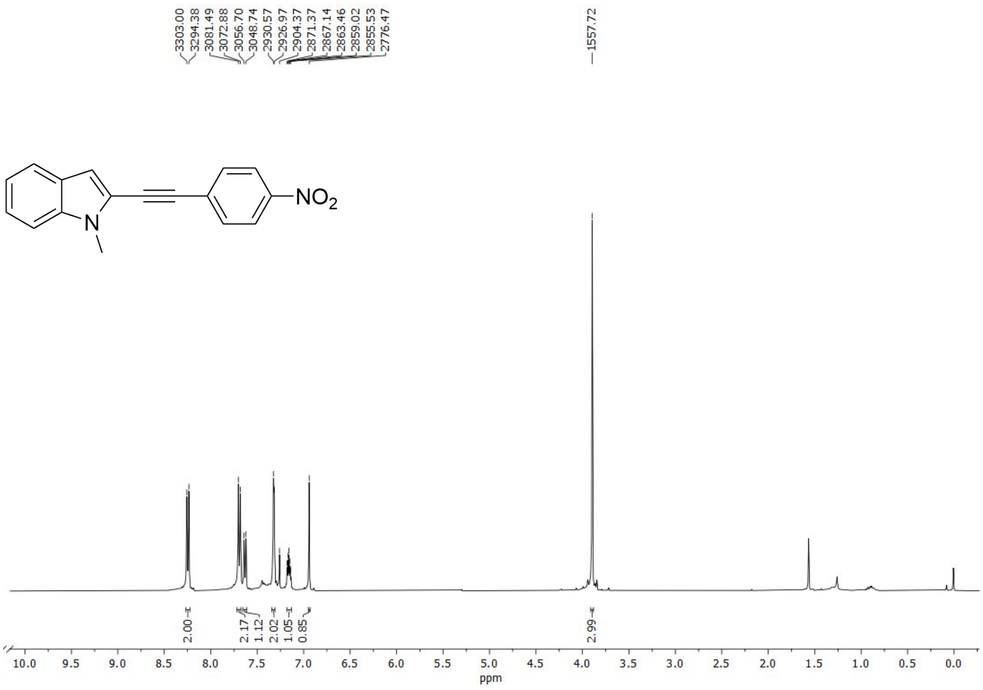

Figure S7. ^{1}H NMR spectrum of **8a** in $CDCl_3$ solution (400 MHz).

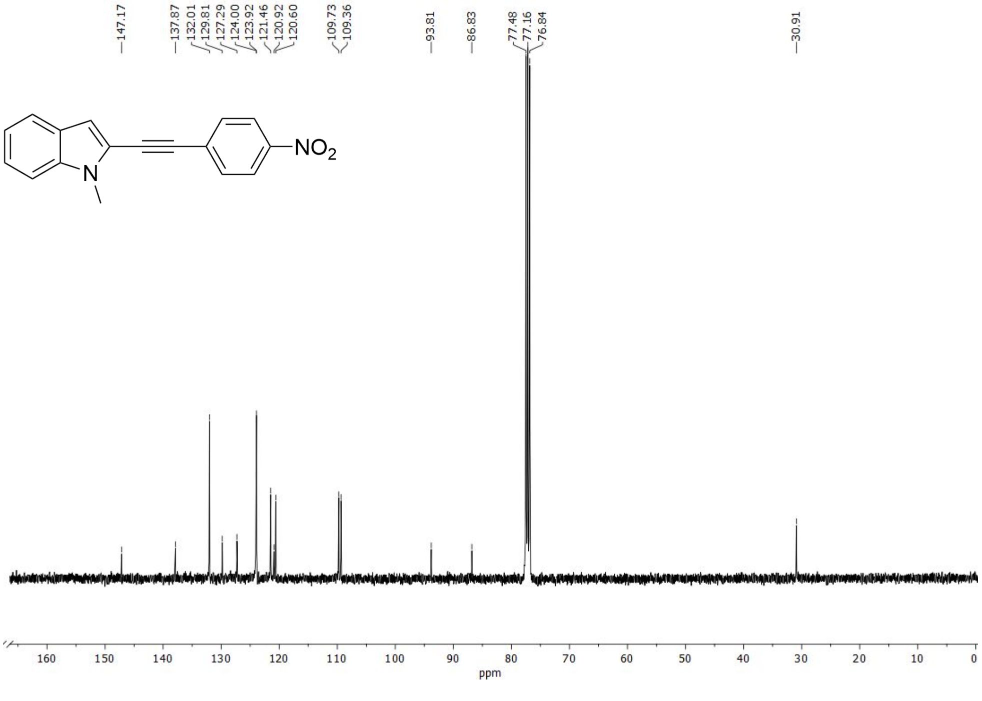

Figure S8. $^{13}C\{^{1}H\}$ NMR spectrum of **8a** in $CDCl_3$ solution (100 MHz).

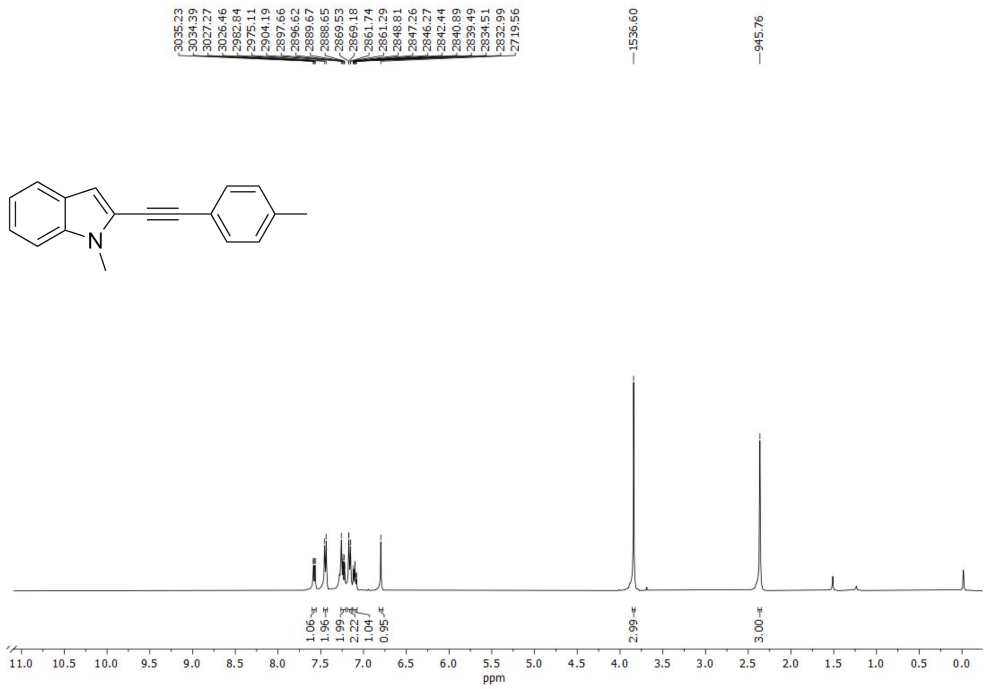

Figure S9. ^{1}H NMR spectrum of **8b** in $CDCl_3$ solution (400 MHz).

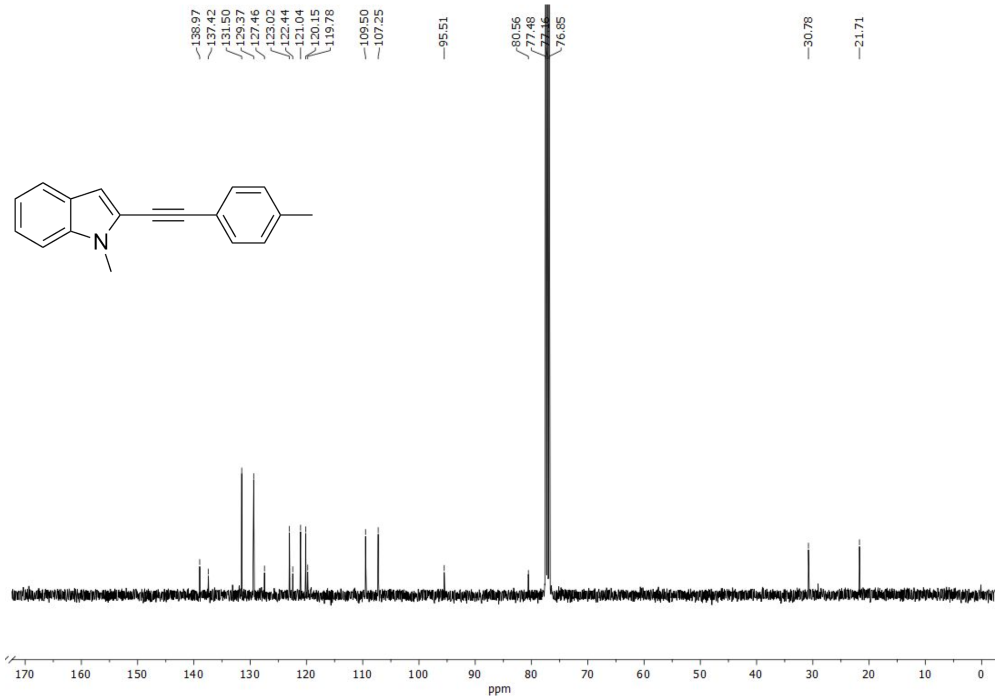

Figure S10. $^{13}C\{^{1}H\}$ NMR spectrum of **8b** in $CDCl_3$ solution (100 MHz).

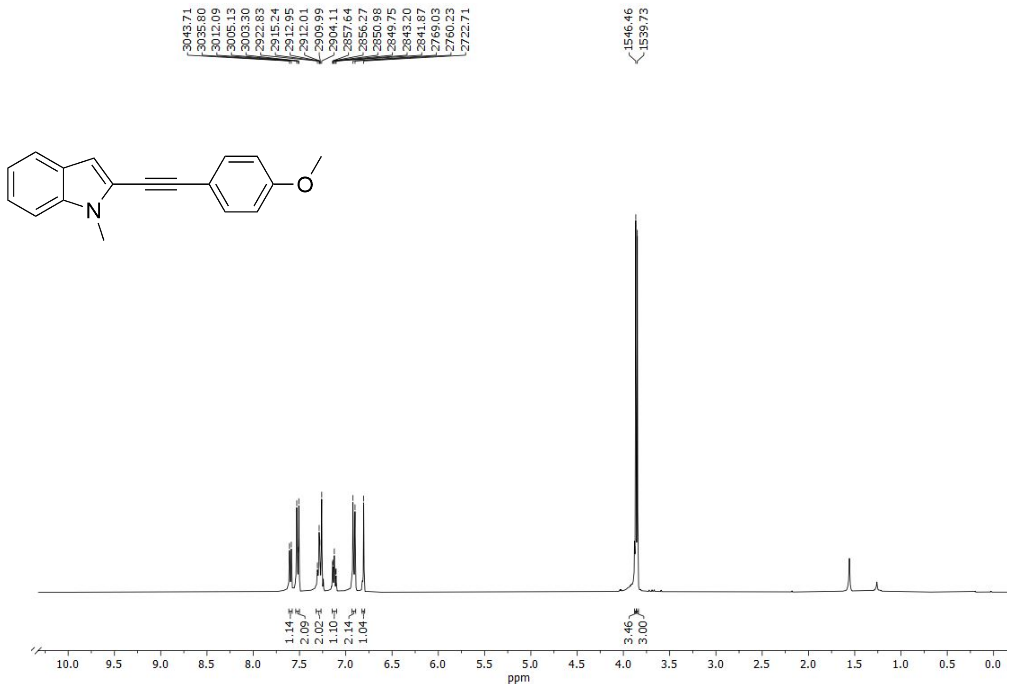

Figure S11. ^{1}H NMR spectrum of **8c** in $CDCl_3$ solution (400 MHz).

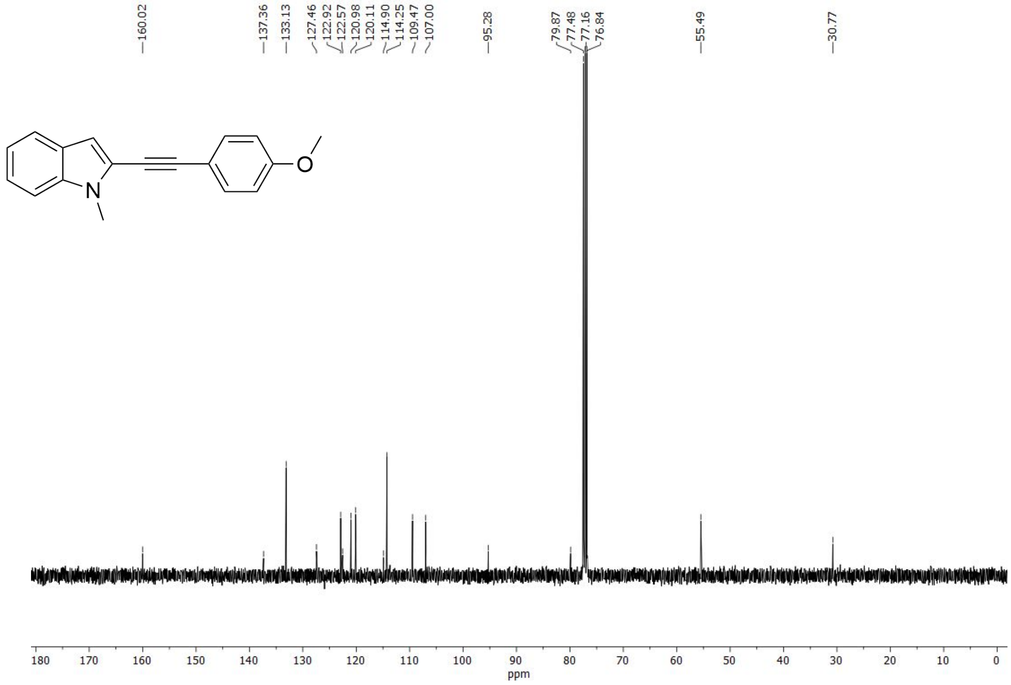

Figure S12. $^{13}C\{^{1}H\}$ NMR spectrum of **8c** in $CDCl_3$ solution (100 MHz).

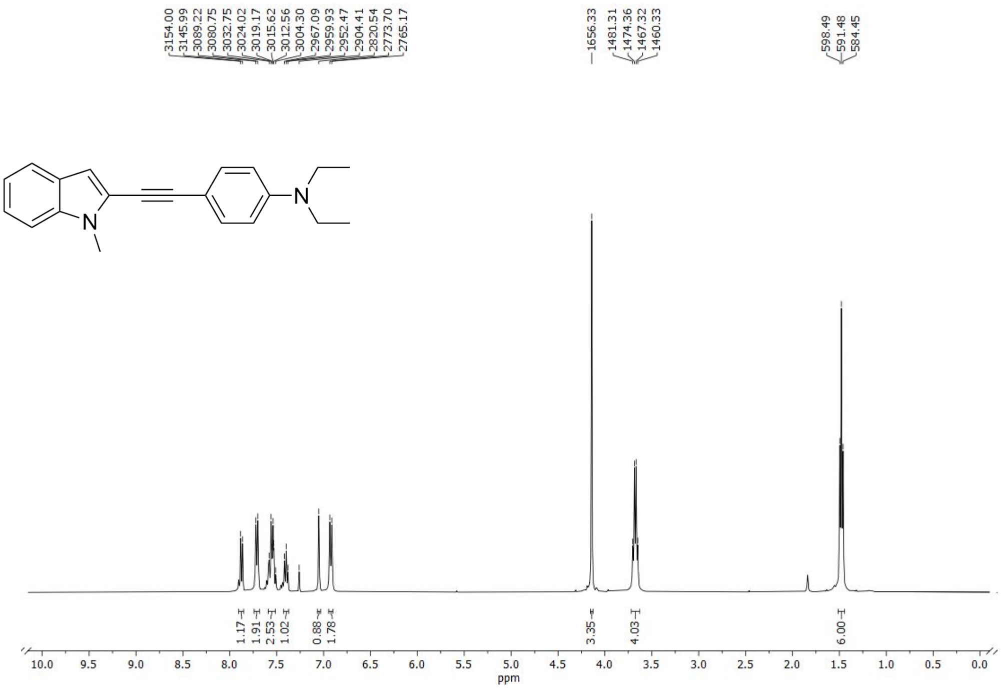

Figure S13. ^{1}H NMR spectrum of **8d** in $CDCl_3$ solution (400 MHz).

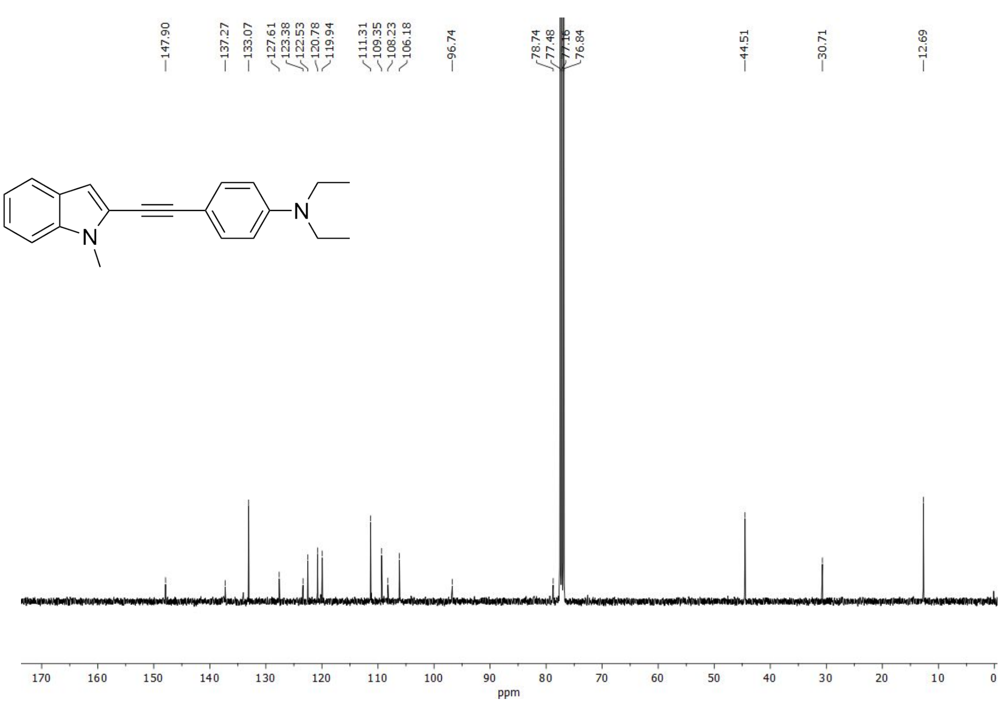

Figure S14. $^{13}C\{^{1}H\}$ NMR spectrum of **8d** in $CDCl_3$ solution (100 MHz).

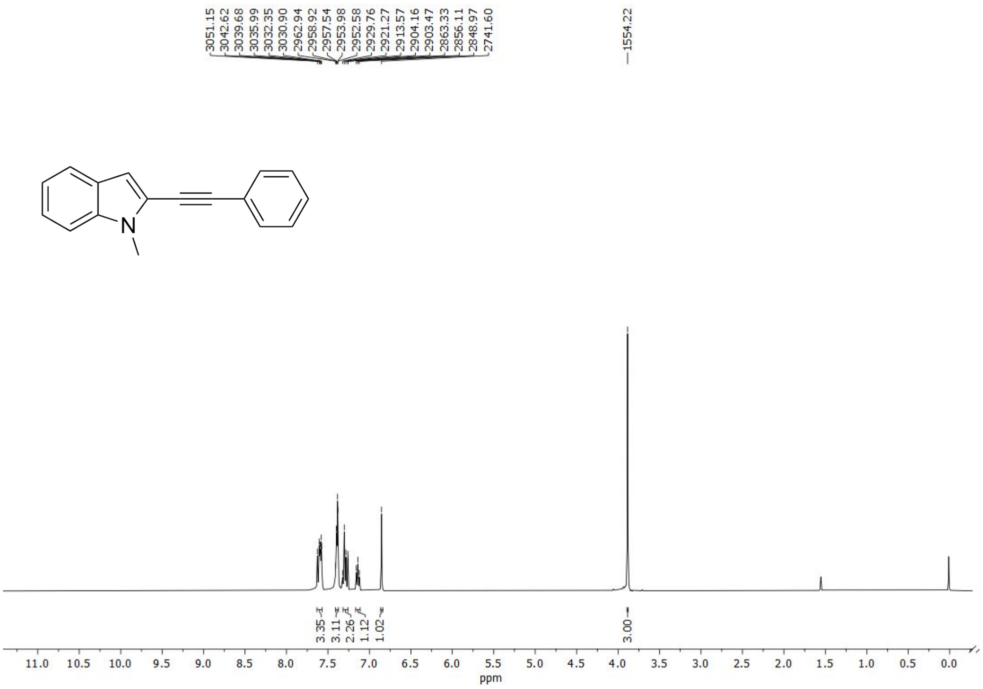

Figure S15. ^{1}H NMR spectrum of **8e** in $CDCl_3$ solution (400 MHz).

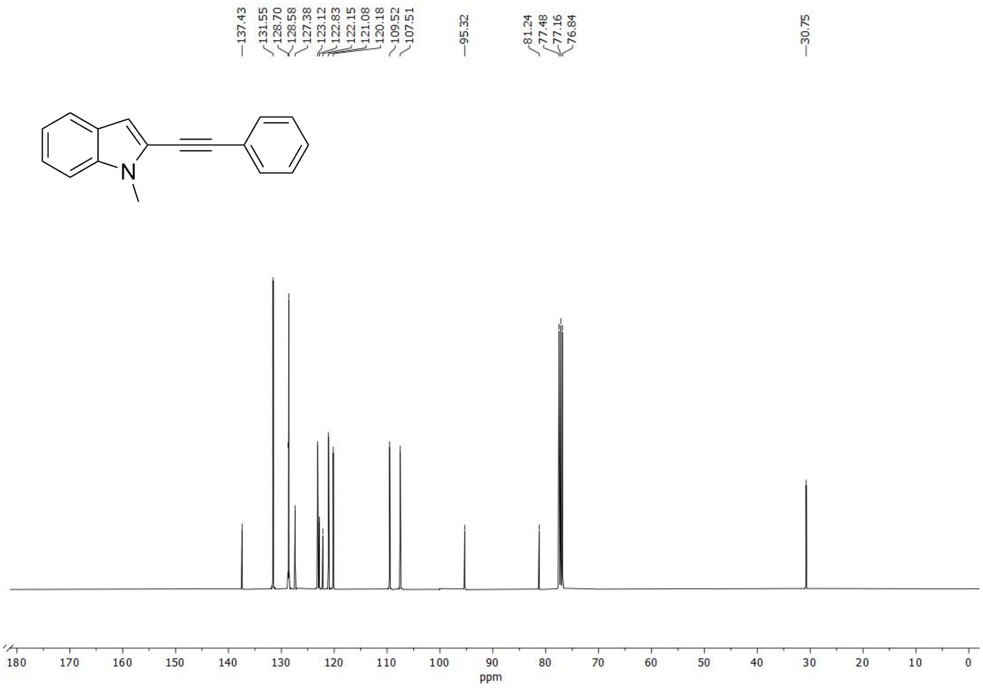

Figure S16. $^{13}C\{^{1}H\}$ NMR spectrum of **8e** in $CDCl_3$ solution (100 MHz).

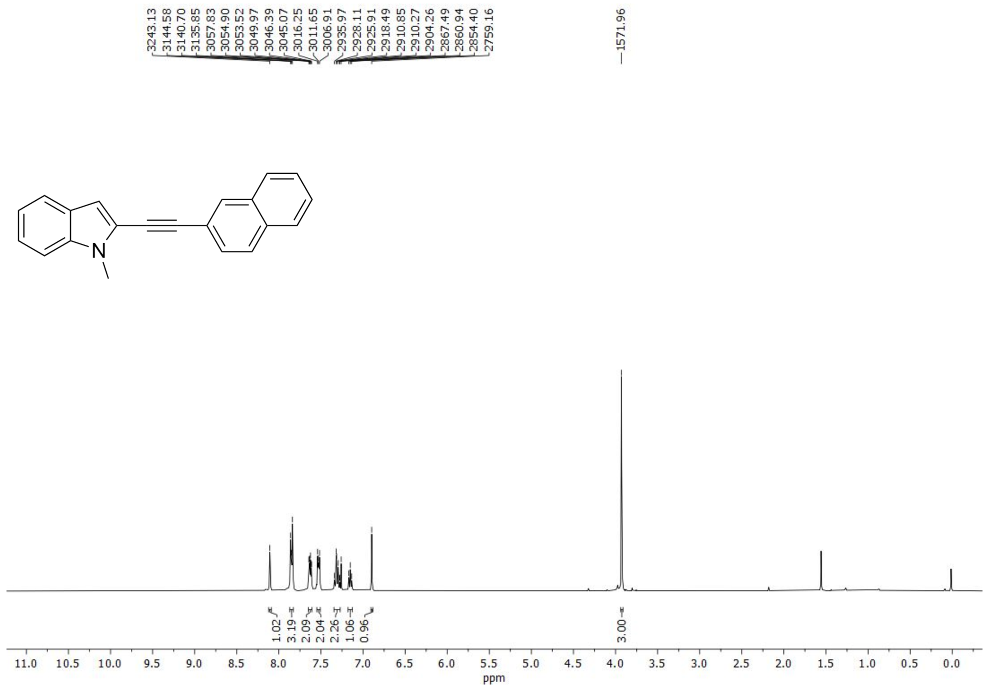

Figure S17. ^{1}H NMR spectrum of **8f** in $CDCl_3$ solution (400 MHz).

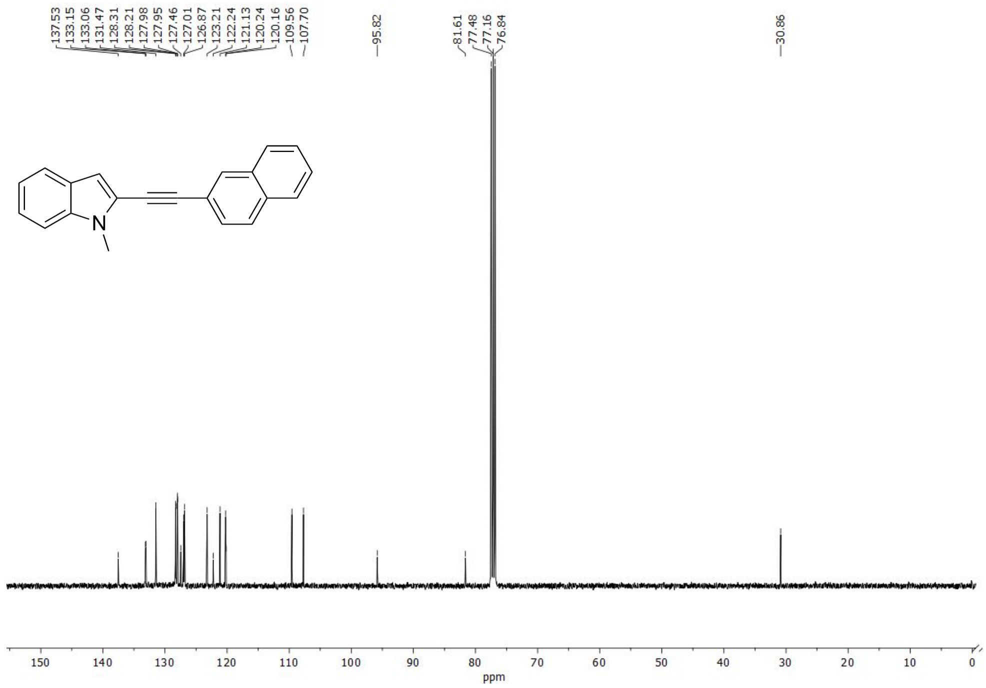

Figure S18. $^{13}C\{^1H\}$ NMR spectrum of **8f** in $CDCl_3$ solution (100 MHz).

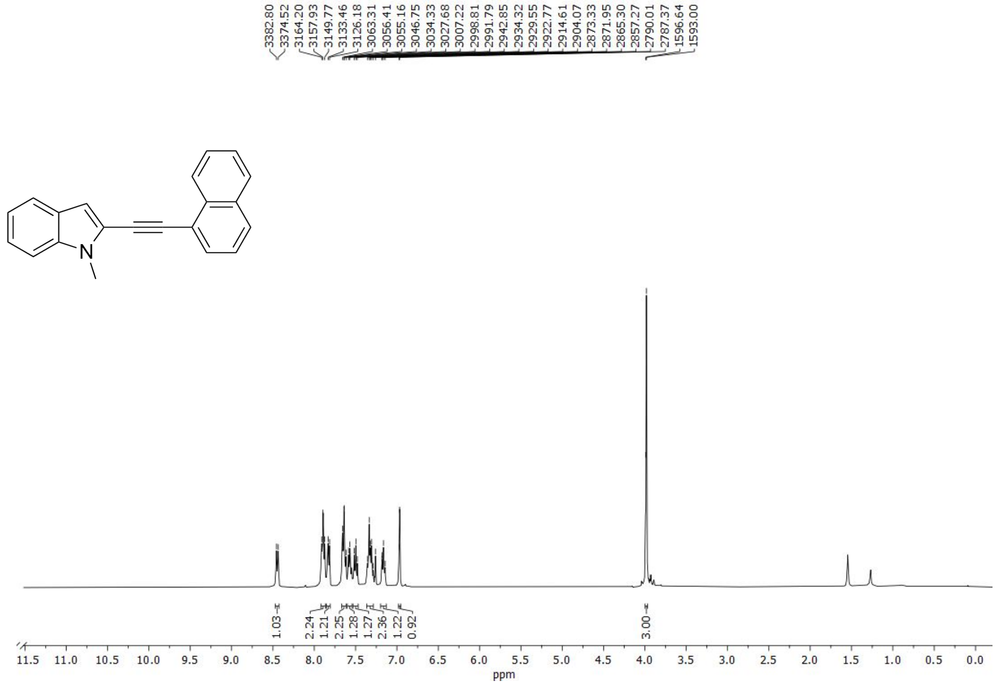

Figure S19. ^{1}H NMR spectrum of **8g** in $CDCl_3$ solution (400 MHz).

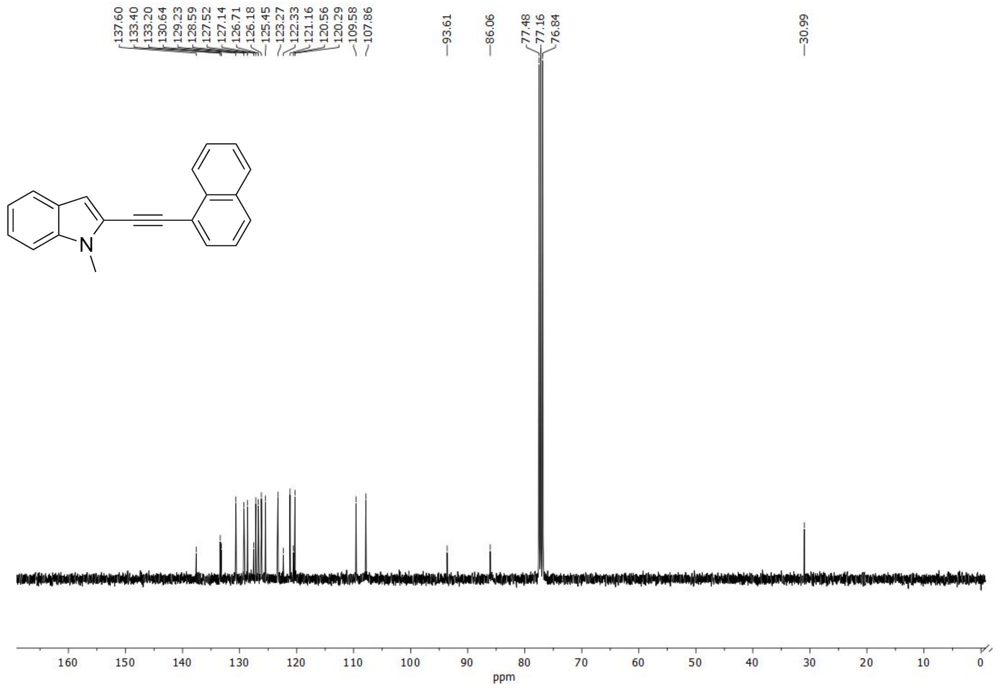

Figure S20. $^{13}C\{^{1}H\}$ NMR spectrum of **8g** in $CDCl_3$ solution (100 MHz).

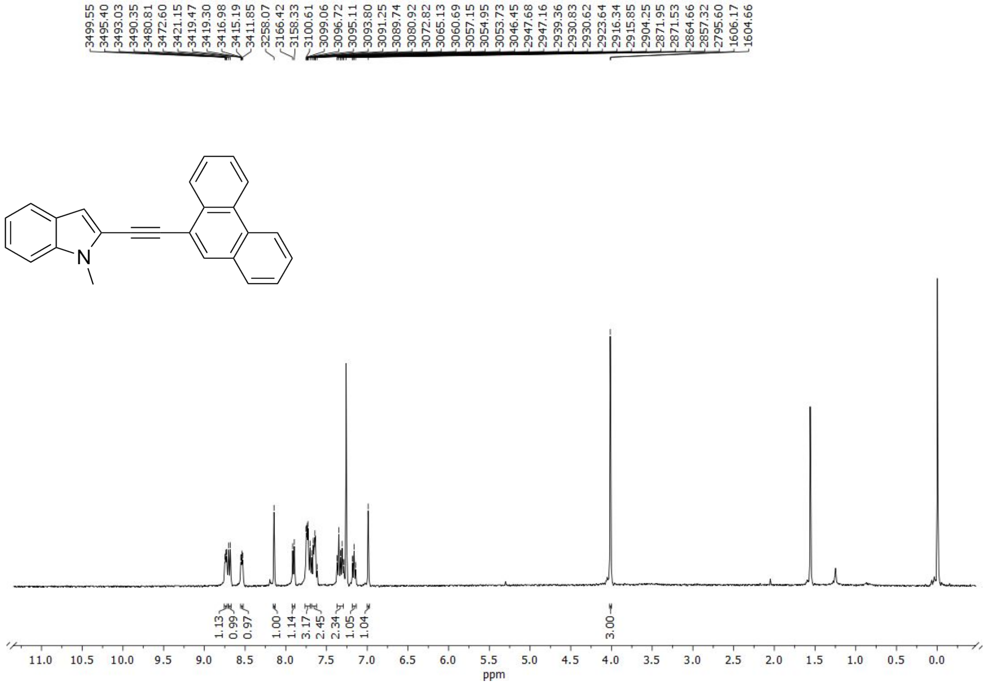

Figure S21. ^{1}H NMR spectrum of **8h** in $CDCl_3$ solution (400 MHz).

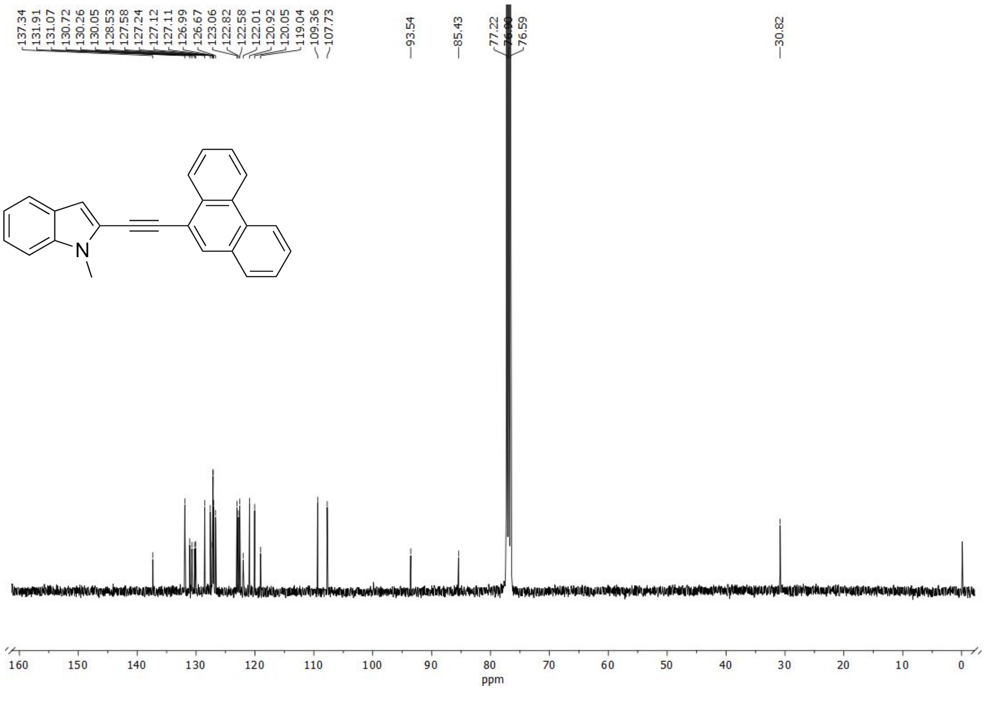

Figure S22. $^{13}C\{^1H\}$ NMR spectrum of **8h** in $CDCl_3$ solution (100 MHz).

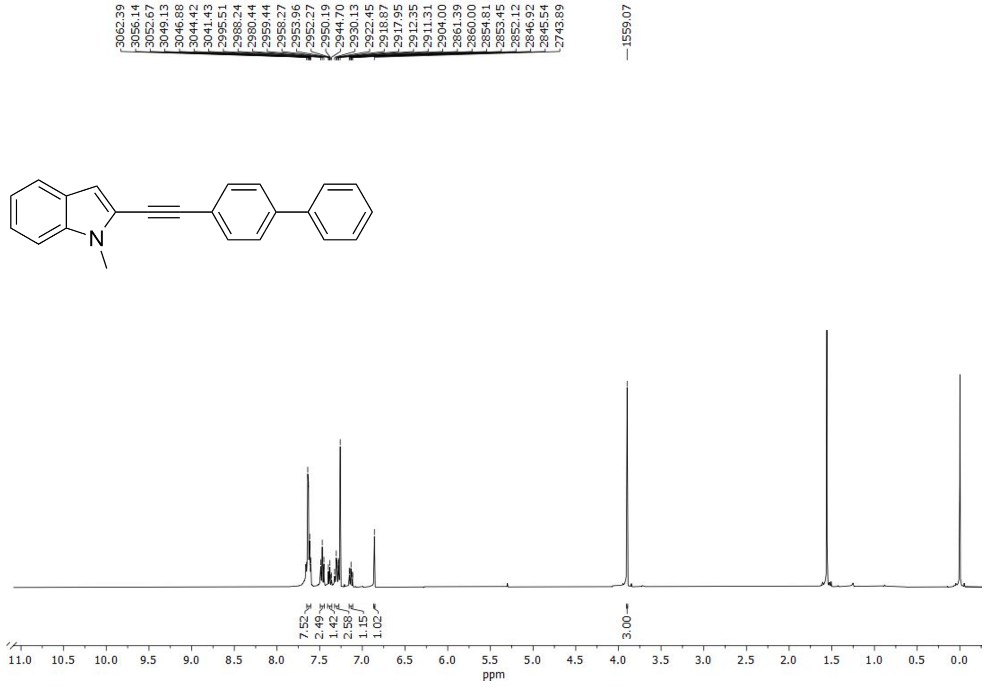

Figure S23. ^{1}H NMR spectrum of **8i** in $CDCl_3$ solution (400 MHz).

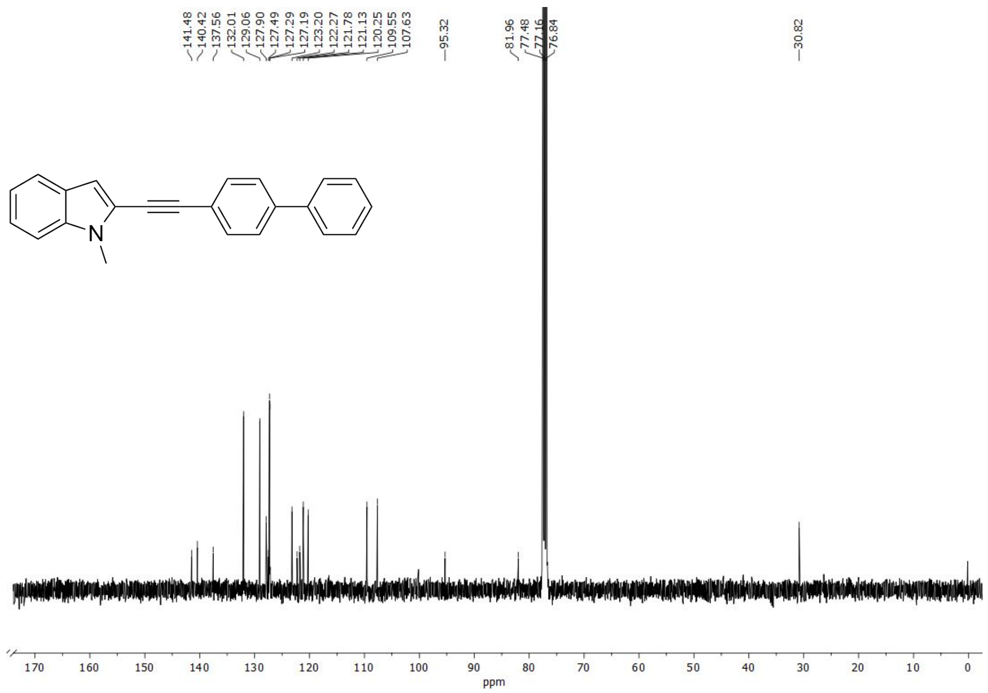

Figure S24. $^{13}C\{^{1}H\}$ NMR spectrum of **8i** in $CDCl_3$ solution (100 MHz).

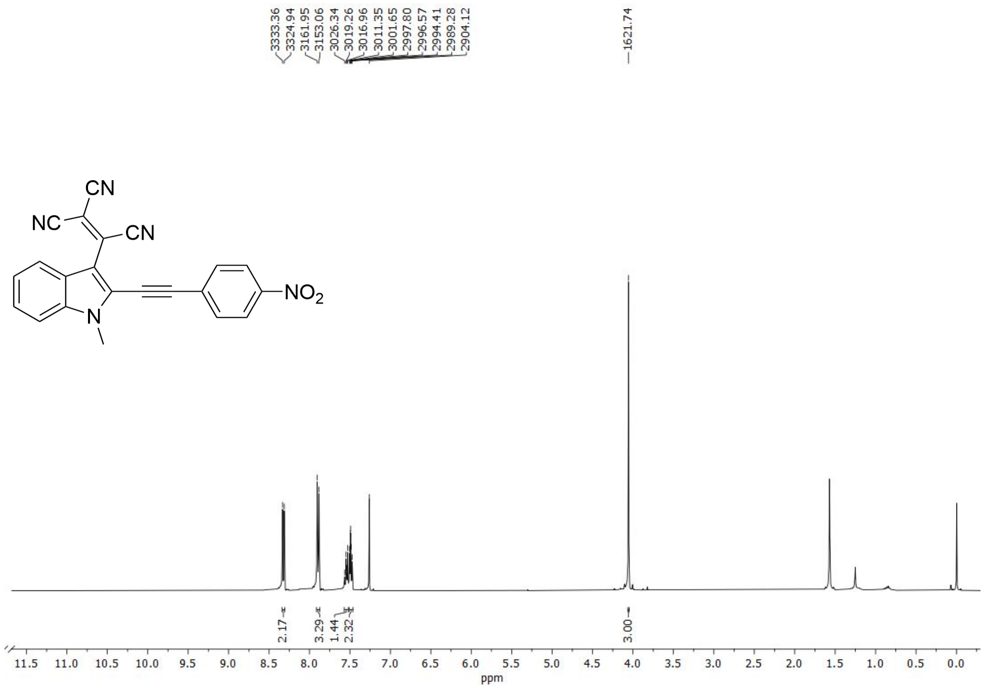

Figure S25. ^{1}H NMR spectrum of **12a** in $CDCl_3$ solution (400 MHz).

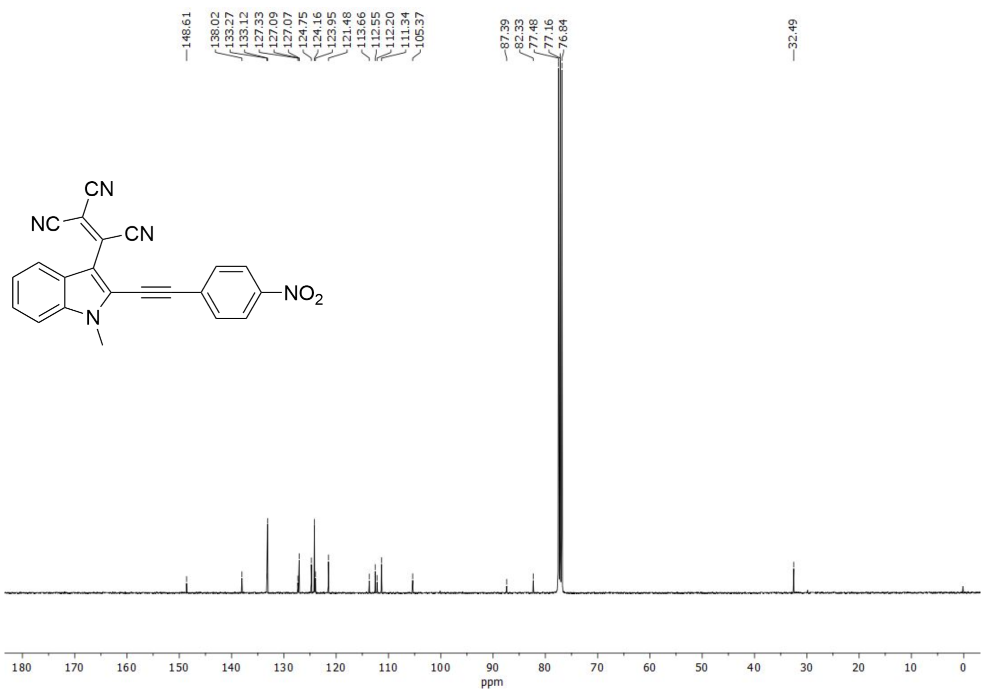

Figure S26. $^{13}C\{^{1}H\}$ NMR spectrum of **12a** in $CDCl_3$ solution (100 MHz).

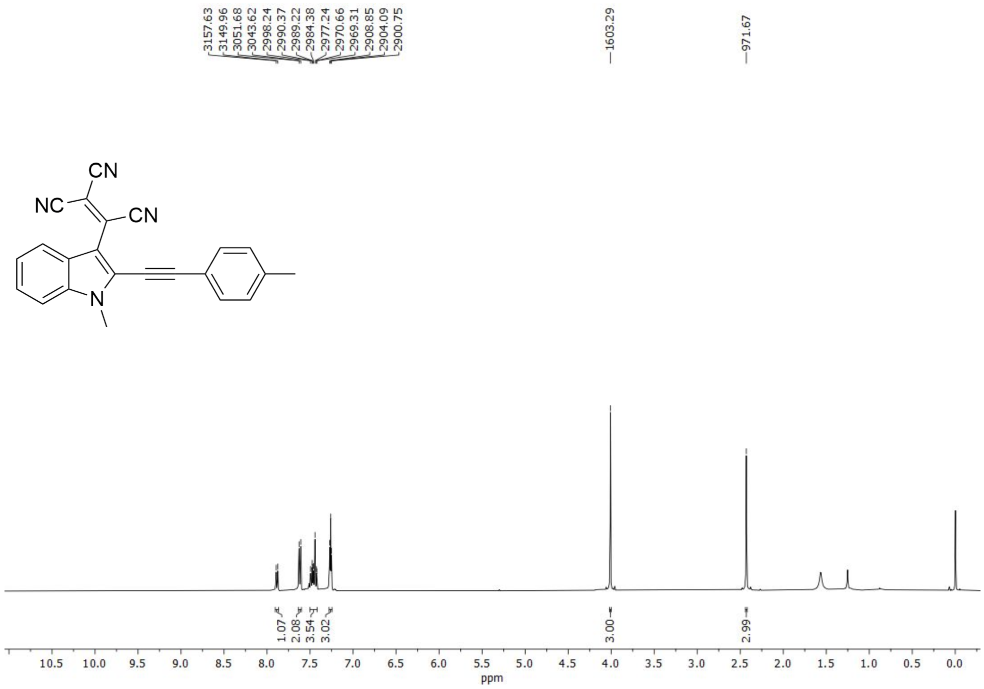

Figure S27. ^{1}H NMR spectrum of **12b** in $CDCl_3$ solution (400 MHz).

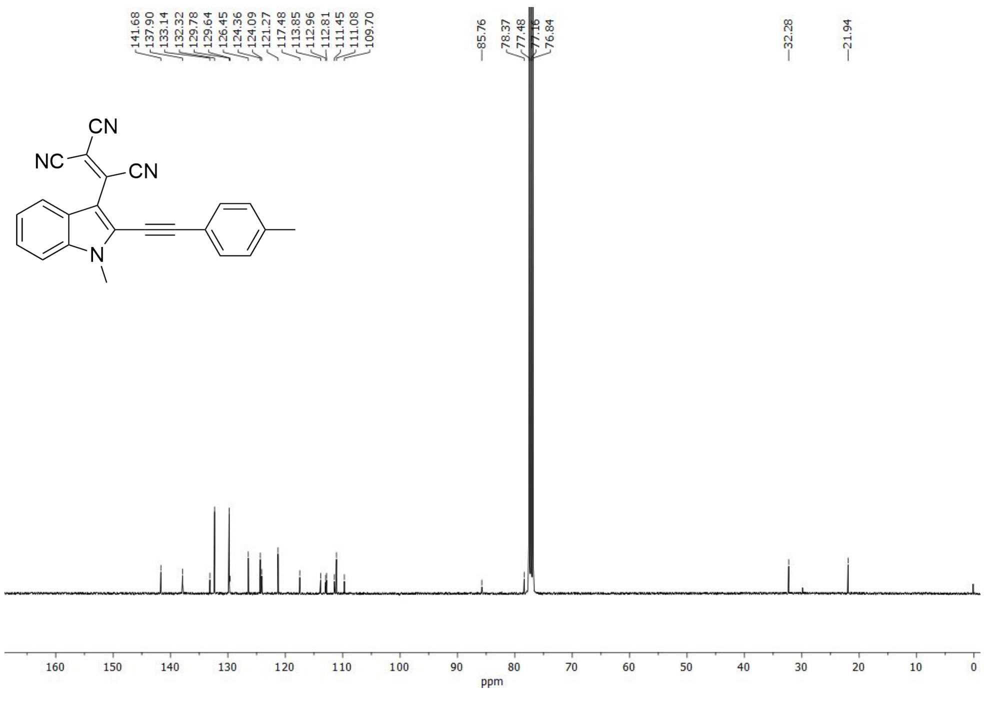

Figure S28. $^{13}C\{^{1}H\}$ NMR spectrum of **12b** in $CDCl_3$ solution (100 MHz).

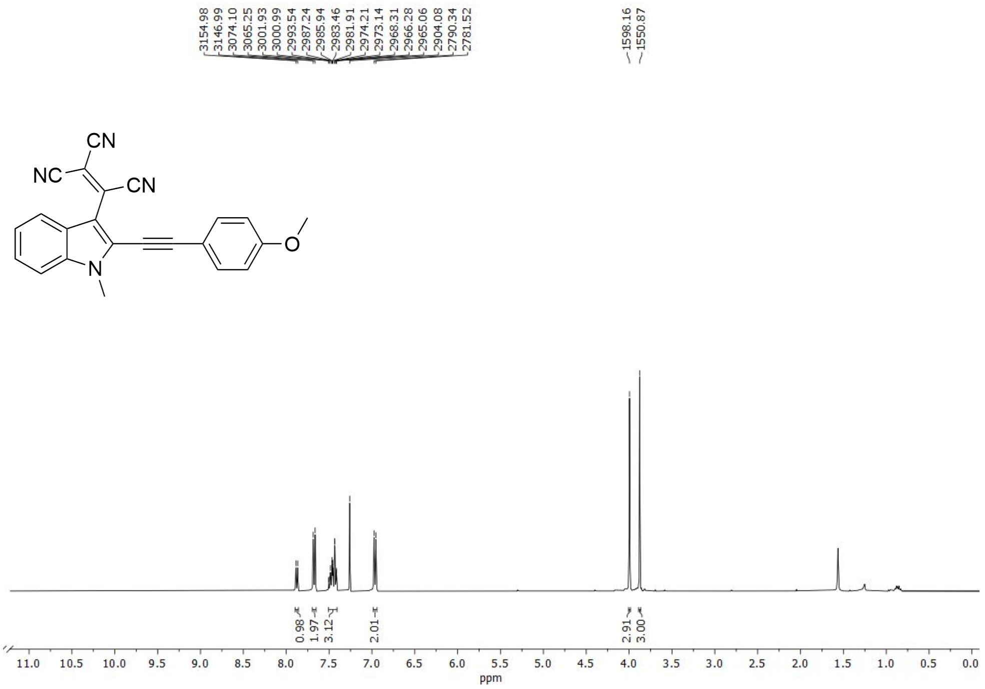

Figure S29. ^{1}H NMR spectrum of **12c** in $CDCl_3$ solution (400 MHz).

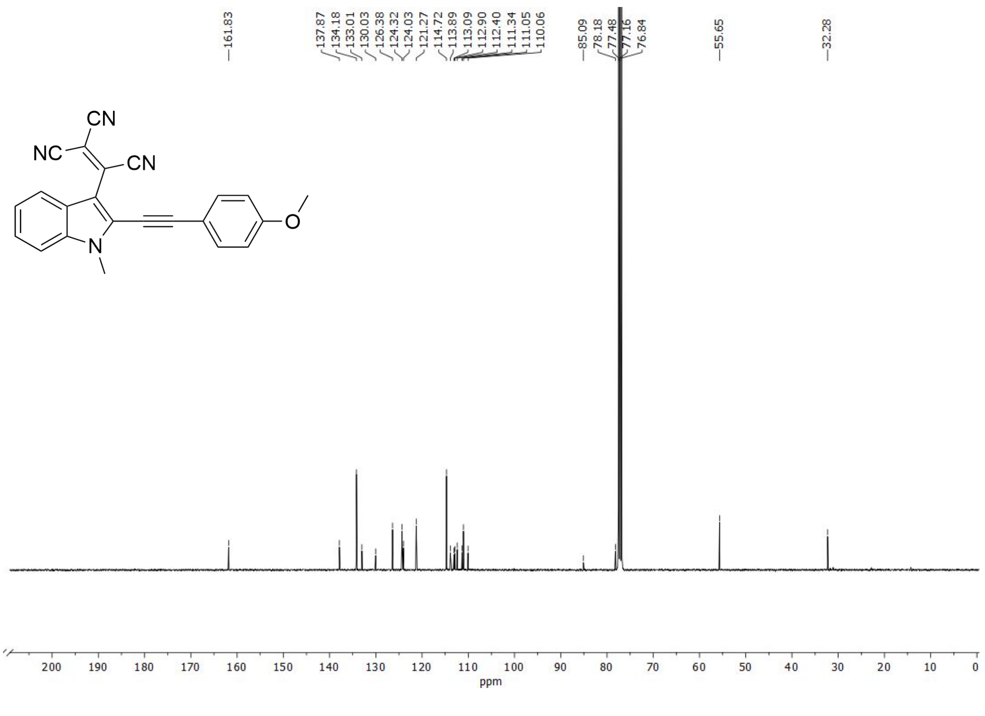

Figure S30. $^{13}C\{^{1}H\}$ NMR spectrum of **12c** in $CDCl_3$ solution (100 MHz).

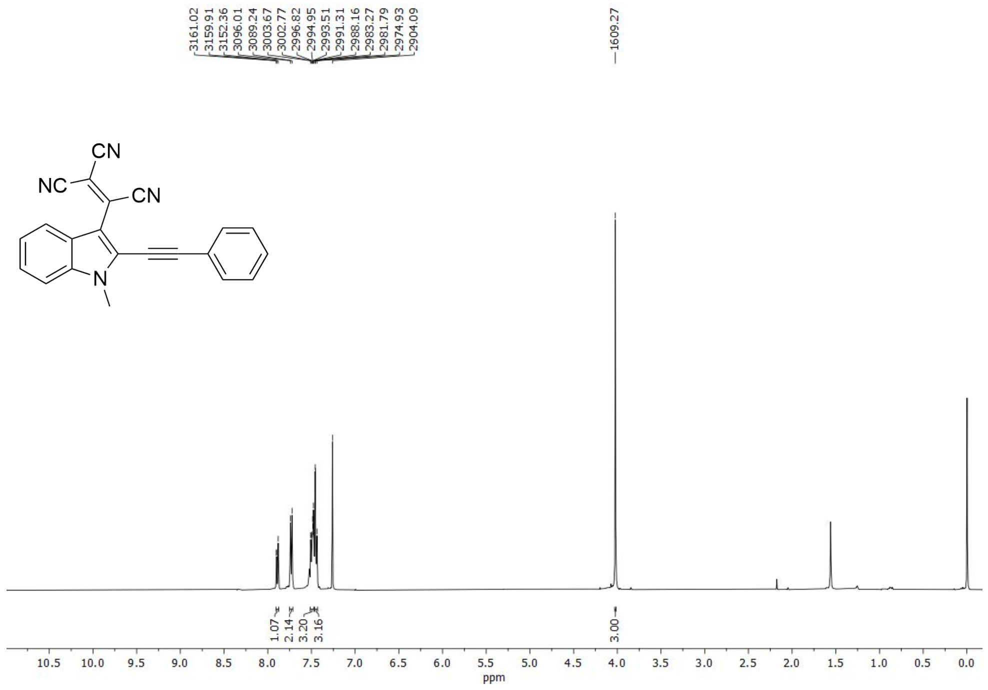

Figure S31. ^{1}H NMR spectrum of **12e** in $CDCl_3$ solution (400 MHz).

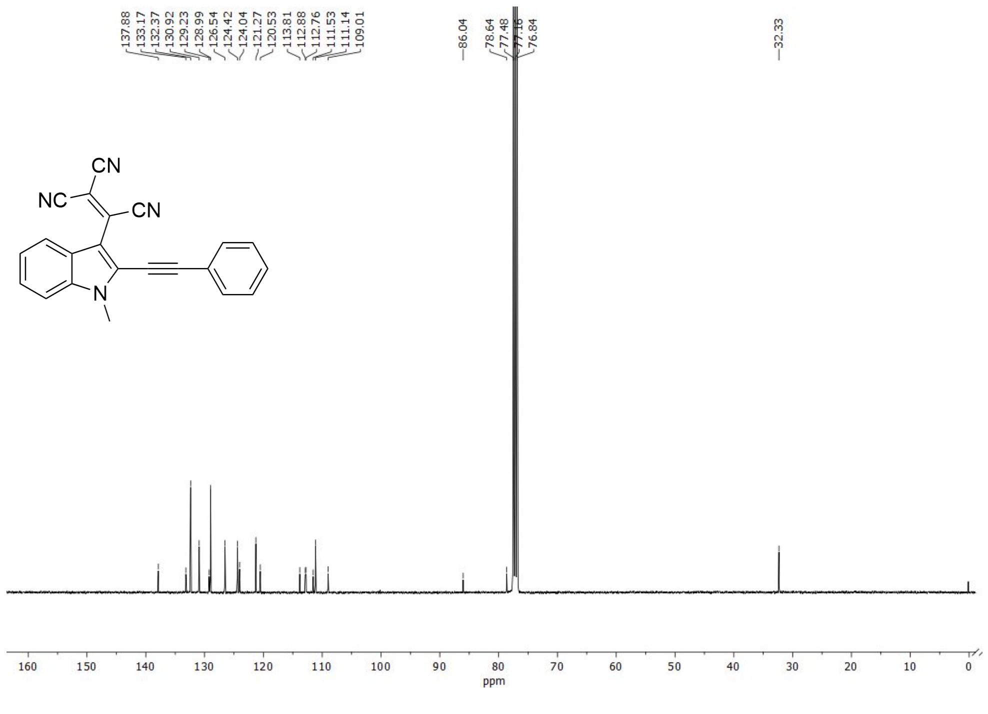

Figure S32. $^{13}C\{^1H\}$ NMR spectrum of **12e** in $CDCl_3$ solution (100 MHz).

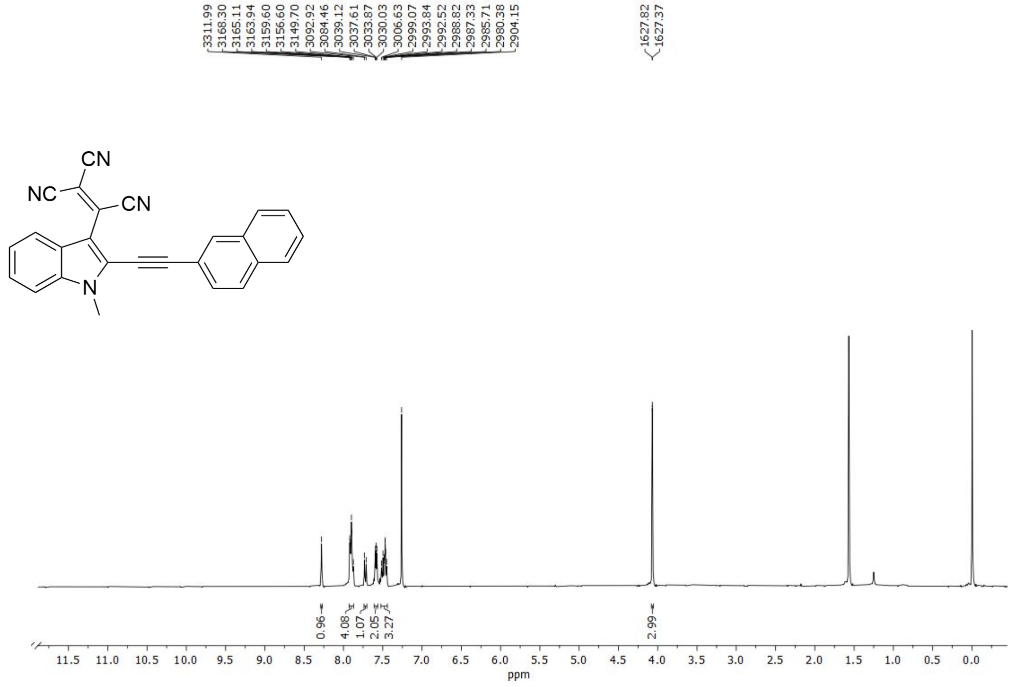

Figure S33. ^{1}H NMR spectrum of **12f** in $CDCl_3$ solution (400 MHz).

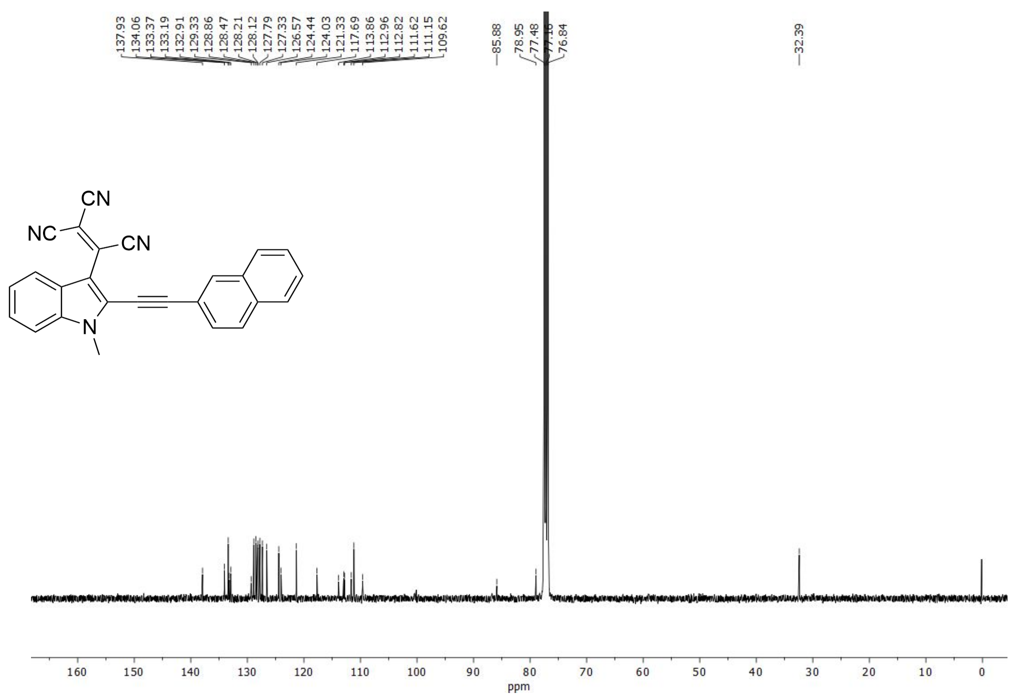

Figure S34. $^{13}C\{^{1}H\}$ NMR spectrum of **12f** in $CDCl_3$ solution (100 MHz).

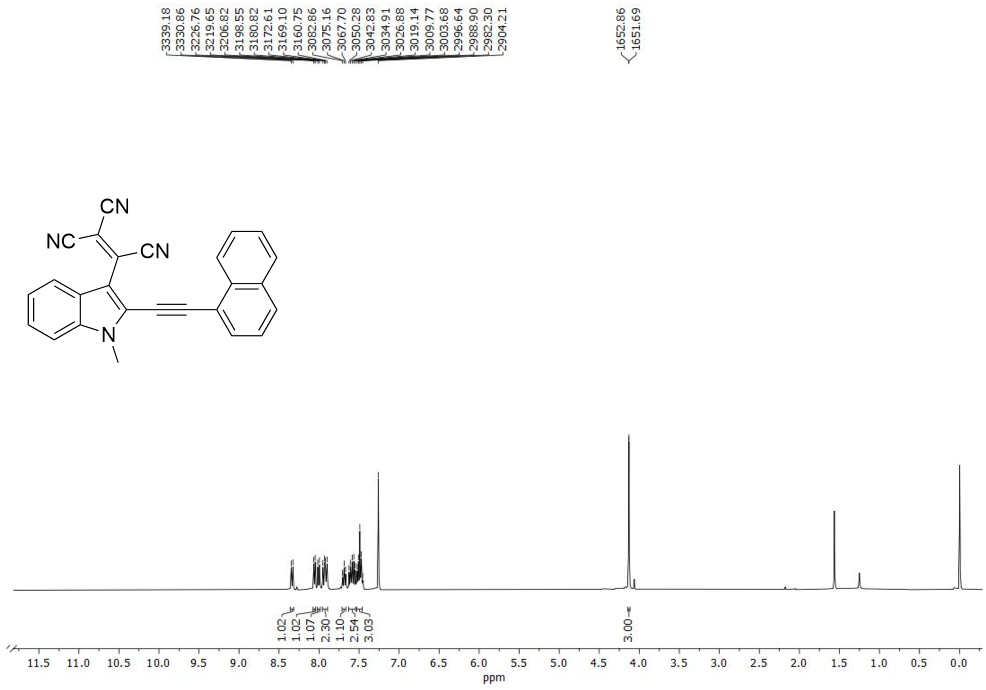

Figure S35. ^{1}H NMR spectrum of **12g** in $CDCl_3$ solution (400 MHz).

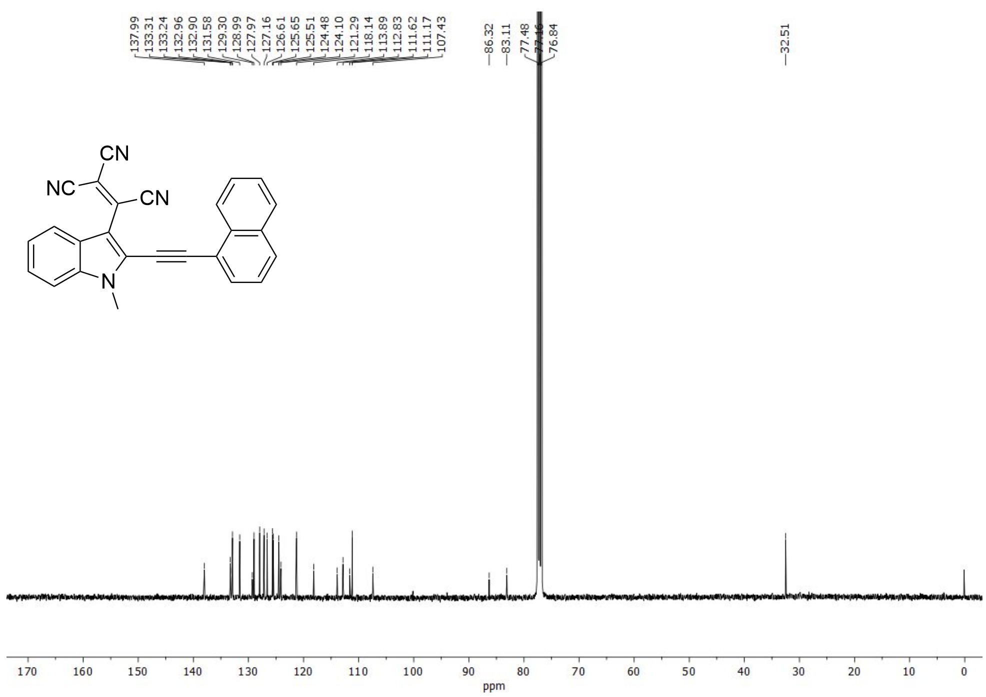

Figure S36. $^{13}C\{^{1}H\}$ NMR spectrum of **12g** in $CDCl_3$ solution (100 MHz).

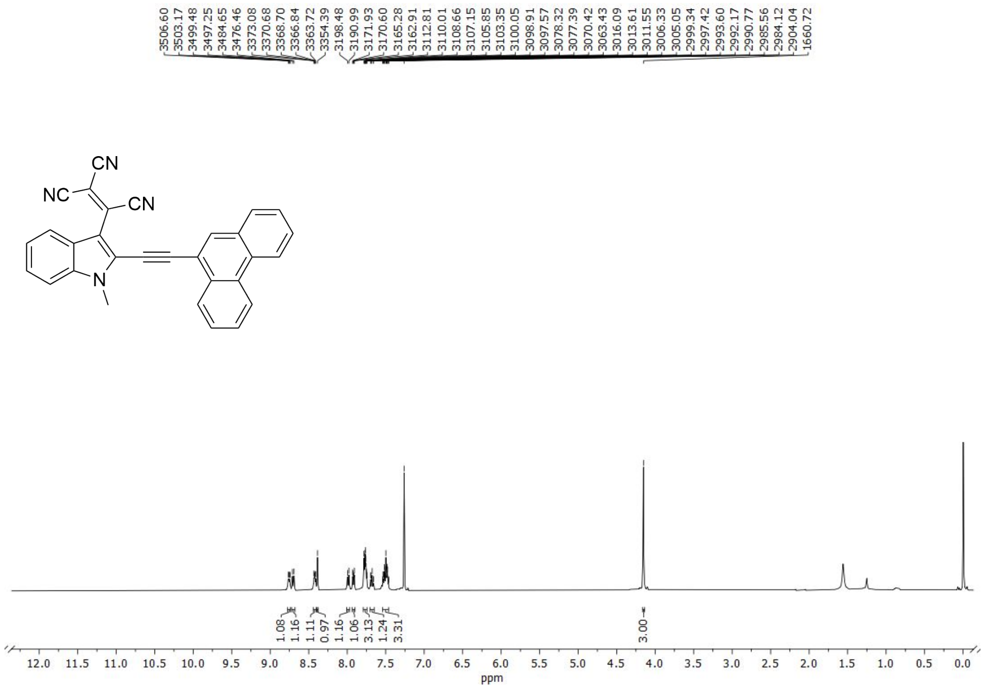

Figure S37. ^{1}H NMR spectrum of **12h** in $CDCl_3$ solution (400 MHz).

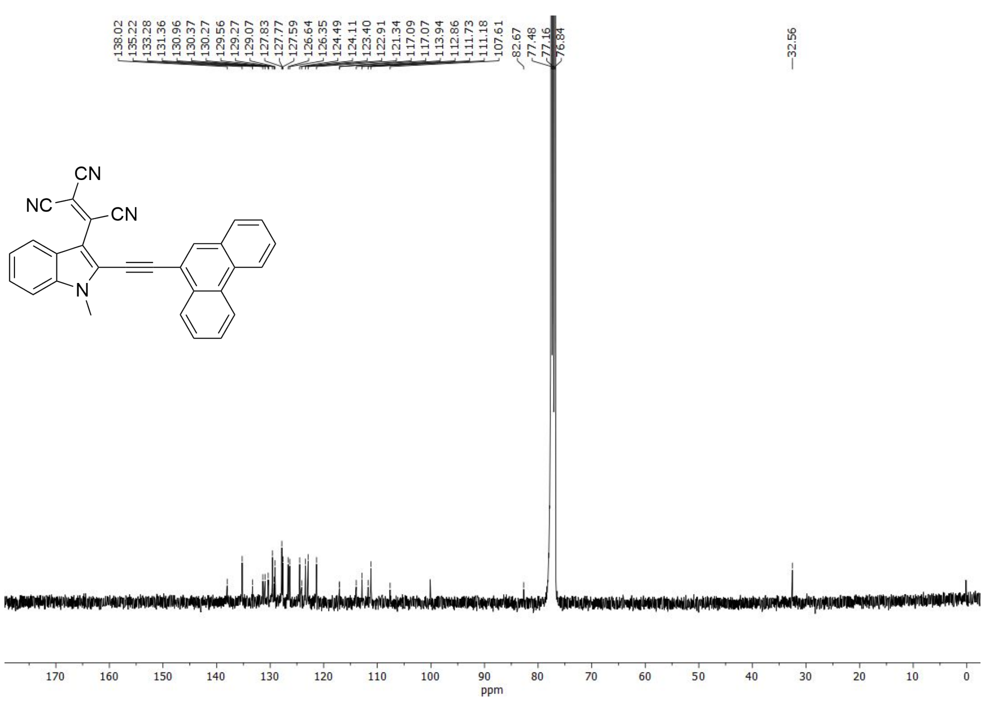

Figure S38. $^{13}C\{^{1}H\}$ NMR spectrum of **12h** in $CDCl_3$ solution (100 MHz).

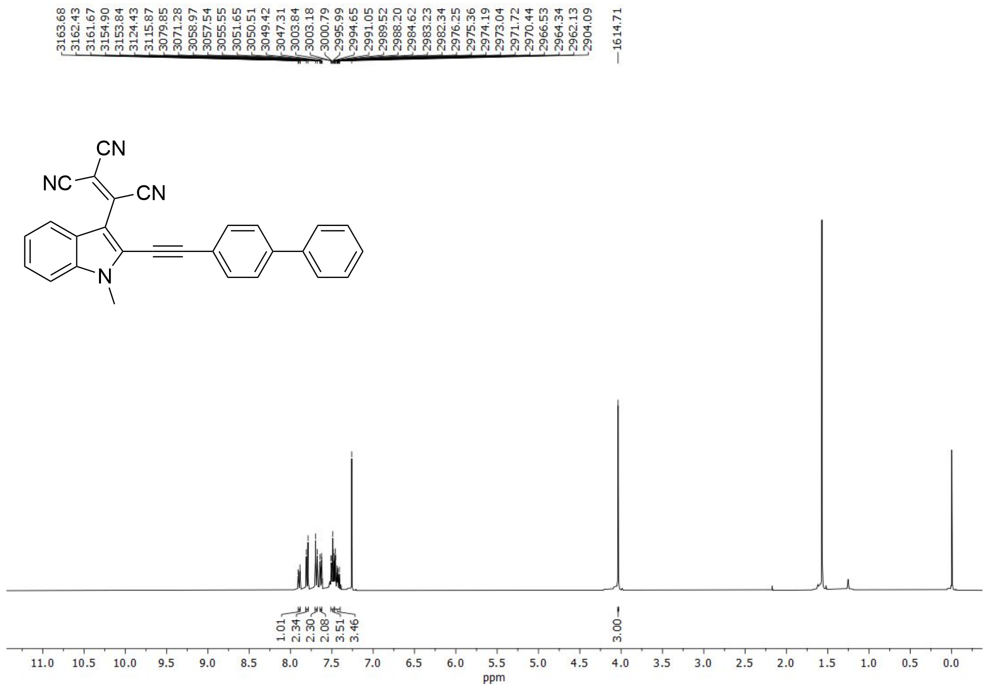

Figure S39. ^{1}H NMR spectrum of **12i** in $CDCl_3$ solution (400 MHz).

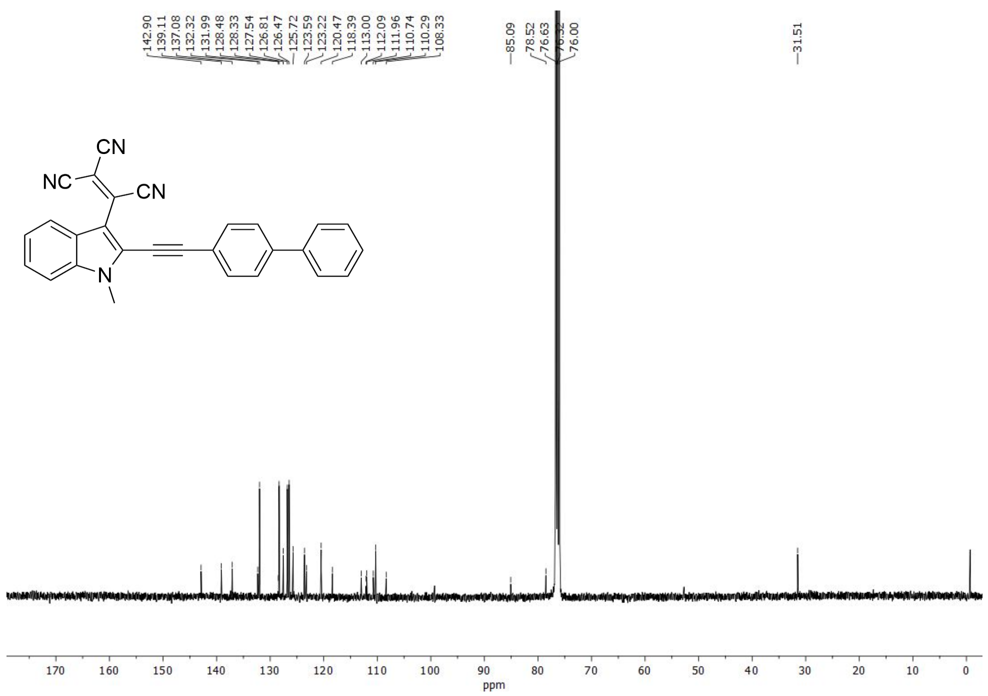

Figure S40. $^{13}C\{^{1}H\}$ NMR spectrum of **12i** in $CDCl_3$ solution (100 MHz).

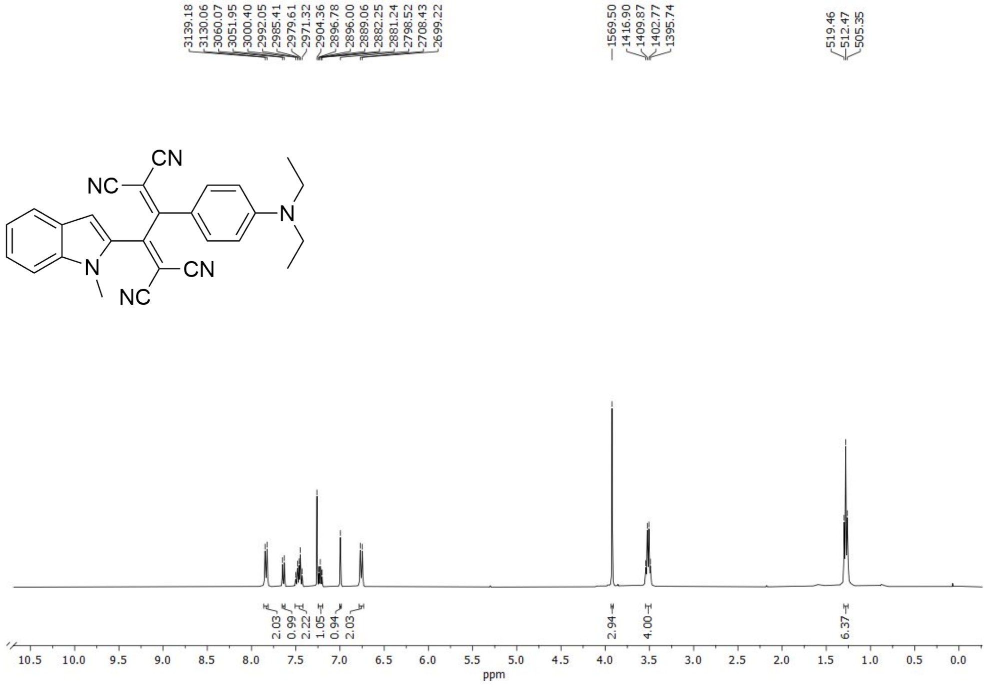

Figure S41. ^{1}H NMR spectrum of **13** in $CDCl_3$ solution (400 MHz).

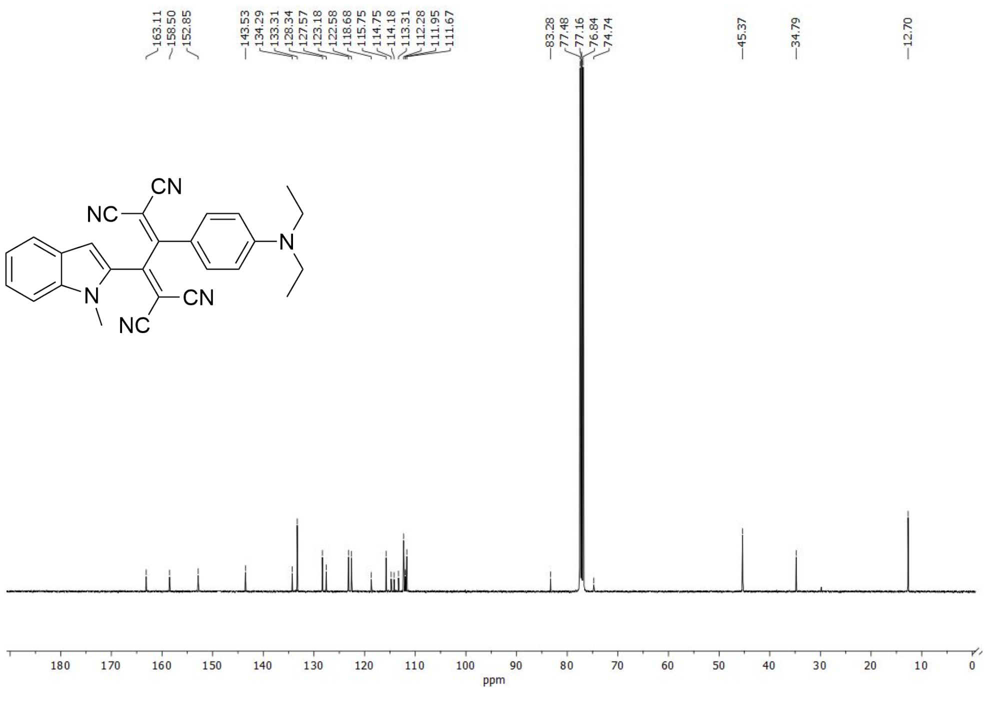

Figure S42. $^{13}C\{^{1}H\}$ NMR spectrum of **13** in $CDCl_3$ solution (100 MHz).

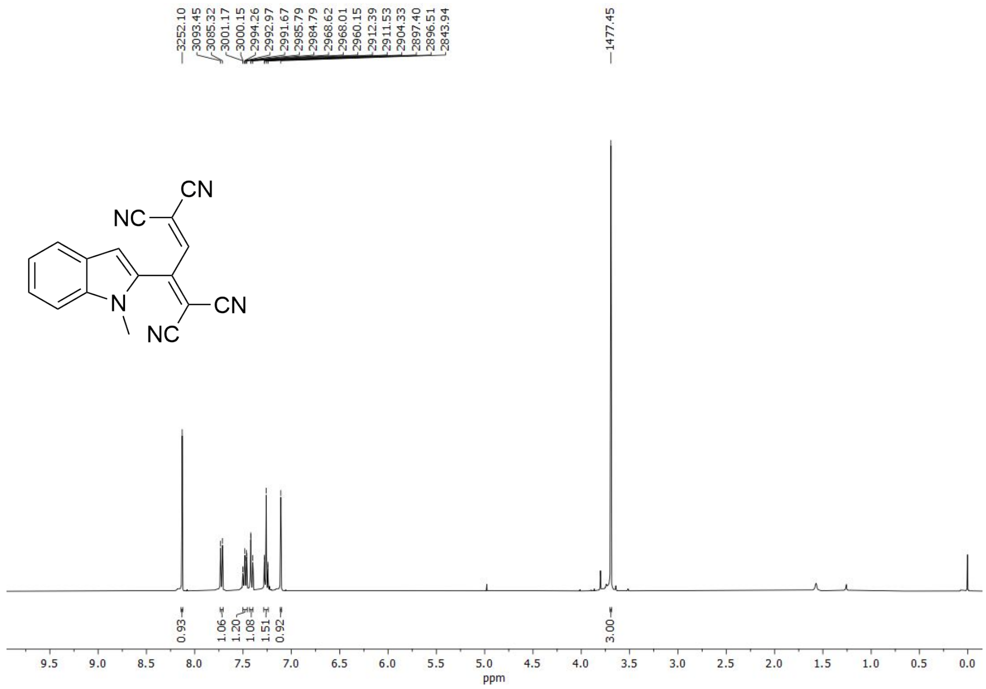

Figure S43. ^{1}H NMR spectrum of **14** in $CDCl_3$ solution (400 MHz).

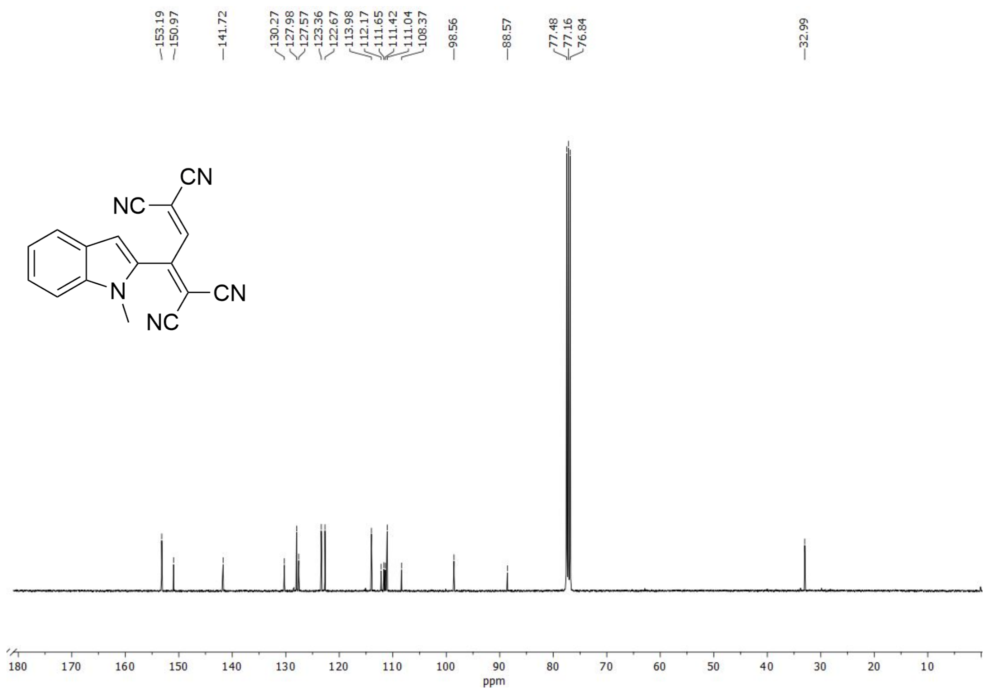

Figure S44. $^{13}C\{^{1}H\}$ NMR spectrum of **14** in $CDCl_3$ solution (100 MHz).

2. High-Resolution Mass Spectrometry (HR-MS) Data

Elemental Composition Report Page 1

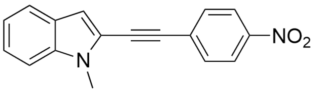

Single Mass Analysis
Tolerance = 1000.0 PPM / DBE: min = -1.5, max = 50.0
Element prediction: Off
Number of isotope peaks used for i-FIT = 3

Monoisotopic Mass, Odd and Even Electron Ions
1 formula(e) evaluated with 1 results within limits (up to 50 best isotopic matches for each mass)
Elements Used:
C: 17-17 H: 12-13 N: 2-2 O: 2-2
Hazal Kayas
44957_20250204_06-02 25 (0.965) Cm (17:25)
1: TOF MS ES+
1.51e+005

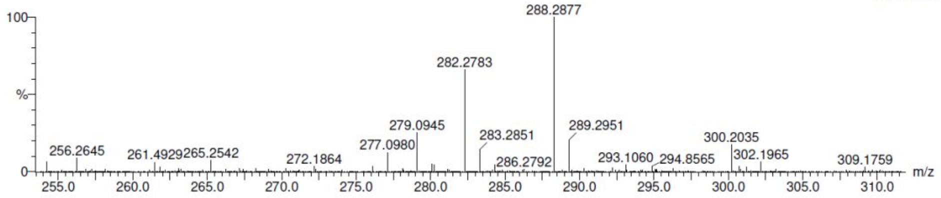

Minimum:				-1.5			
Maximum:		100.0	1000.0	50.0			
Mass	Calc. Mass	mDa	PPM	DBE	i-FIT	i-FIT (Norm)	Formula
277.0980	277.0977	0.3	1.1	12.5	302.3	0.0	C17 H13 N2 O2

Figure S45. HR-MS spectrum of **8a**.

Elemental Composition Report Page 1

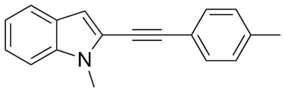

Single Mass Analysis
Tolerance = 1000.0 PPM / DBE: min = -5.5, max = 1000.0
Element prediction: Off
Number of isotope peaks used for i-FIT = 9

Monoisotopic Mass, Odd and Even Electron Ions
2 formula(e) evaluated with 1 results within limits (all results (up to 1000) for each mass)
Elements Used:
C: 18-18 H: 15-16 N: 1-1 Na: 0-1
Hazal Kayas
43096_20240731_02-01 14 (0.552) Cm (11:17)
1: TOF MS ES+
2.97e+003

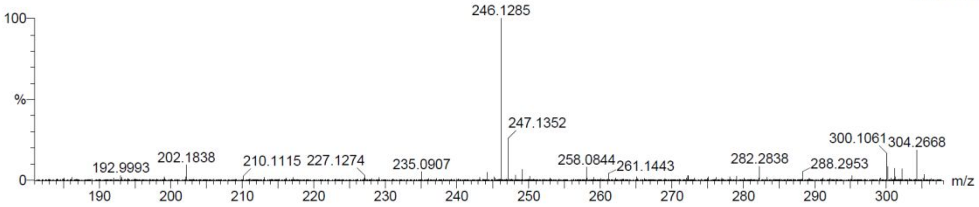

Minimum:				-5.5			
Maximum:		1000.0	1000.0	1000.0			
Mass	Calc. Mass	mDa	PPM	DBE	i-FIT	i-FIT (Norm)	Formula
246.1285	246.1283	0.2	0.8	11.5	520.1	0.0	C18 H16 N

Figure S46. HR-MS spectrum of **8b**.

Elemental Composition Report **Page 1**

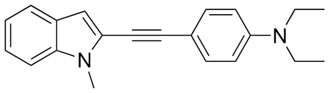

Single Mass Analysis
Tolerance = 1000.0 PPM / DBE: min = -5.5, max = 1000.0
Element prediction: Off
Number of isotope peaks used for i-FIT = 9

Monoisotopic Mass, Odd and Even Electron Ions
1 formula(e) evaluated with 1 results within limits (all results (up to 1000) for each mass)
Elements Used:
C: 21-21 H: 22-23 N: 2-2 Na: 0-1
Hazal Kayas
43096_20240807_01-05 3 (0.138) Cm (3:7) 1: TOF MS ES+ 1.65e+006

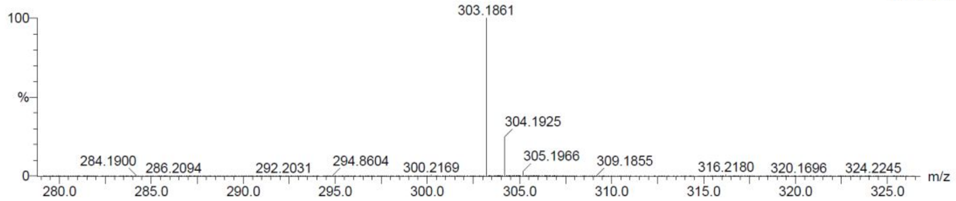

Minimum:				-5.5			
Maximum:		1000.0	1000.0	1000.0			
Mass	Calc. Mass	mDa	PPM	DBE	i-FIT	i-FIT (Norm)	Formula
303.1861	303.1861	0.0	0.0	11.5	1273.1	0.0	C21 H23 N2

Figure S47. HR-MS spectrum of **8d**.

Elemental Composition Report **Page 1**

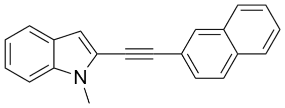

Single Mass Analysis
Tolerance = 1000.0 PPM / DBE: min = -1.5, max = 50.0
Element prediction: Off
Number of isotope peaks used for i-FIT = 3

Monoisotopic Mass, Odd and Even Electron Ions
1 formula(e) evaluated with 1 results within limits (up to 50 best isotopic matches for each mass)
Elements Used:
C: 21-21 H: 15-16 N: 1-1
Hazal Kayas
44957_20250204_04-01 13 (0.518) Cm (10:25) 1: TOF MS ES+ 1.05e+004

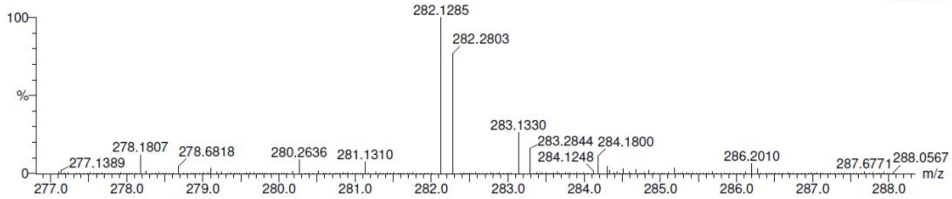

Minimum:				-1.5			
Maximum:		100.0	1000.0	50.0			
Mass	Calc. Mass	mDa	PPM	DBE	i-FIT	i-FIT (Norm)	Formula
282.1285	282.1283	0.2	0.7	14.5	373.5	0.0	C21 H16 N

Figure S48. HR-MS spectrum of **8f**.

Elemental Composition Report **Page 1**

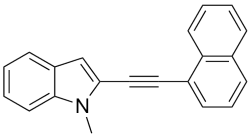

Single Mass Analysis
Tolerance = 1000.0 PPM / DBE: min = -5.5, max = 1000.0
Element prediction: Off
Number of isotope peaks used for i-FIT = 9

Monoisotopic Mass, Odd and Even Electron Ions
2 formula(e) evaluated with 1 results within limits (all results (up to 1000) for each mass)
Elements Used:
C: 21-21 H: 15-16 N: 1-1 Na: 0-1
Hazal Kayas
43096_20240731_03-01 8 (0.328) Cm (7:10)

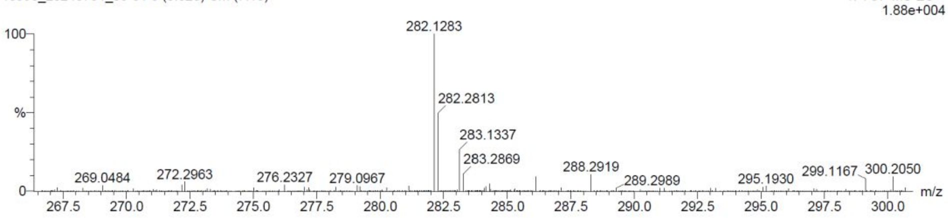

Minimum:				-5.5			
Maximum:		1000.0	1000.0	1000.0			
Mass	Calc. Mass	mDa	PPM	DBE	i-FIT	i-FIT (Norm)	Formula
282.1283	282.1283	0.0	0.0	14.5	857.7	0.0	C21 H16 N

Figure S49. HR-MS spectrum of **8g**.

Elemental Composition Report **Page 1**

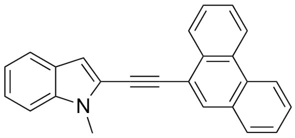

Single Mass Analysis
Tolerance = 1000.0 PPM / DBE: min = -1.5, max = 50.0
Element prediction: Off
Number of isotope peaks used for i-FIT = 3

Monoisotopic Mass, Even Electron Ions
1 formula(e) evaluated with 1 results within limits (up to 50 best isotopic matches for each mass)
Elements Used:
C: 25-25 H: 17-18 N: 1-1
Hazal Kayas
44957_20250204_05-02 20 (0.775) Cm (1:25)

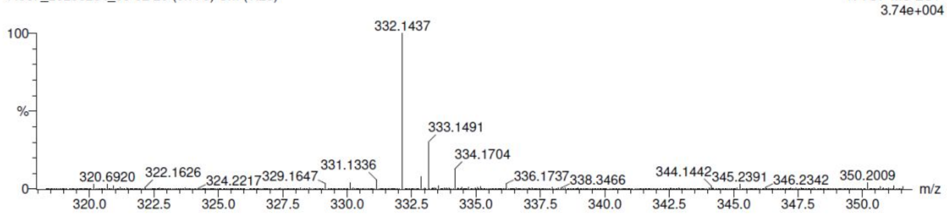

Minimum:				-1.5			
Maximum:		100.0	1000.0	50.0			
Mass	Calc. Mass	mDa	PPM	DBE	i-FIT	i-FIT (Norm)	Formula
332.1437	332.1439	-0.2	-0.6	17.5	413.3	0.0	C25 H18 N

Figure S50. HR-MS spectrum of **8h**.

Elemental Composition Report Page 1

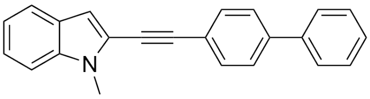

Single Mass Analysis
Tolerance = 20.0 PPM / DBE: min = -1.5, max = 50.0
Element prediction: Off
Number of isotope peaks used for i-FIT = 3

Monoisotopic Mass, Even Electron Ions
1 formula(e) evaluated with 1 results within limits (up to 50 best isotopic matches for each mass)
Elements Used:
C: 23-23 H: 17-20 N: 1-1
Hazal Kayas
46629_20250416_01-16 10 (0.396) Cm (8:13) 1: TOF MS ES+ 8.02e+005

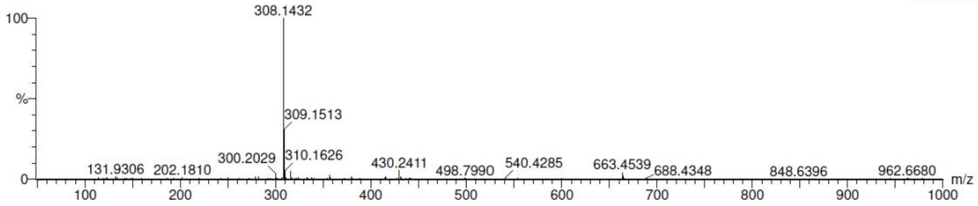

Minimum:				-1.5			
Maximum:		100.0	20.0	50.0			
Mass	Calc. Mass	mDa	PPM	DBE	i-FIT	i-FIT (Norm)	Formula
308.1432	308.1439	-0.7	-2.3	15.5	442.3	0.0	C23 H18 N

Figure S51. HR-MS spectrum of **8i**.

Elemental Composition Report Page 1

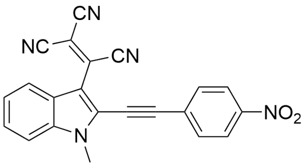

Single Mass Analysis
Tolerance = 1000.0 PPM / DBE: min = -1.5, max = 50.0
Element prediction: Off
Number of isotope peaks used for i-FIT = 3

Monoisotopic Mass, Even Electron Ions
1 formula(e) evaluated with 1 results within limits (up to 50 best isotopic matches for each mass)
Elements Used:
C: 22-22 H: 11-12 N: 5-5 O: 2-2
Hazal Kayas
44957_20250204_07-01 2 (0.104) Cm (1:23) 1: TOF MS ES+ 6.07e+004

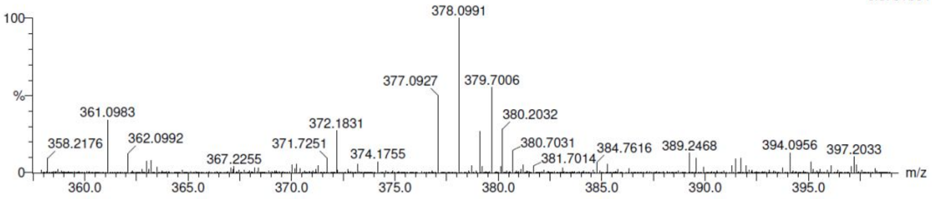

Minimum:				-1.5			
Maximum:		100.0	1000.0	50.0			
Mass	Calc. Mass	mDa	PPM	DBE	i-FIT	i-FIT (Norm)	Formula
378.0991	378.0991	0.0	0.0	19.5	382.4	0.0	C22 H12 N5 O2

Figure S52. HR-MS spectrum of **12a**.

•

Elemental Composition Report **Page 1**

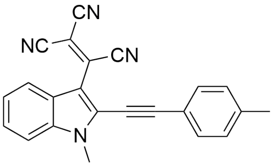

Single Mass Analysis
Tolerance = 1000.0 PPM / DBE: min = -1.5, max = 50.0
Element prediction: Off
Number of isotope peaks used for i-FIT = 3

Monoisotopic Mass, Odd and Even Electron Ions
1 formula(e) evaluated with 1 results within limits (up to 50 best isotopic matches for each mass)
Elements Used:
C: 23-23 H: 14-15 N: 4-4
Hazal Kayas
44957_20250204_11-04 1 (0.070) Cm (1:6) 1: TOF MS ES+ 5.08e+004

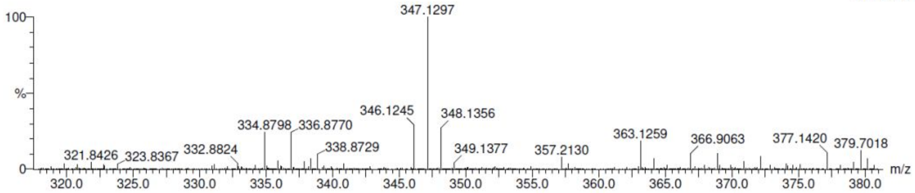

Minimum:				-1.5			
Maximum:		100.0	1000.0	50.0			
Mass	Calc. Mass	mDa	PPM	DBE	i-FIT	i-FIT (Norm)	Formula
347.1297	347.1297	0.0	0.0	18.5	329.9	0.0	C23 H15 N4

Figure S53. HR-MS spectrum of **12b**.

Elemental Composition Report **Page 1**

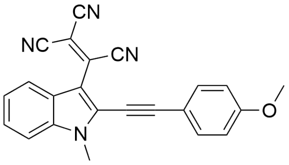

Single Mass Analysis
Tolerance = 1000.0 PPM / DBE: min = -1.5, max = 50.0
Element prediction: Off
Number of isotope peaks used for i-FIT = 3

Monoisotopic Mass, Odd and Even Electron Ions
1 formula(e) evaluated with 1 results within limits (up to 50 best isotopic matches for each mass)
Elements Used:
C: 23-23 H: 14-15 N: 4-4 O: 1-1
Hazal Kayas
44957_20250204_10-04 4 (0.172) Cm (1:25) 1: TOF MS ES+ 1.03e+004

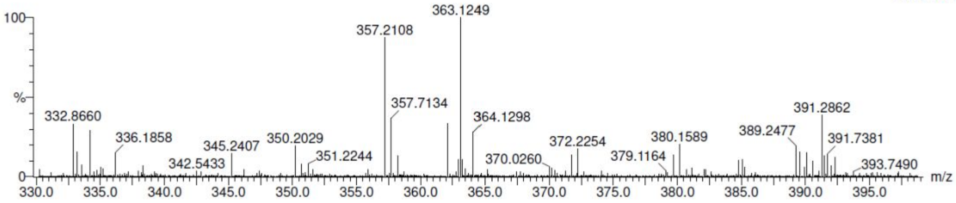

Minimum:				-1.5			
Maximum:		100.0	1000.0	50.0			
Mass	Calc. Mass	mDa	PPM	DBE	i-FIT	i-FIT (Norm)	Formula
363.1249	363.1246	0.3	0.8	18.5	378.8	0.0	C23 H15 N4 O

Figure S54. HR-MS spectrum of **12c**.

Elemental Composition Report **Page 1**

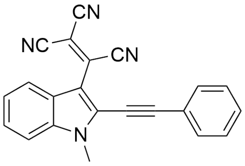

Single Mass Analysis
Tolerance = 1000.0 PPM / DBE: min = -1.5, max = 50.0
Element prediction: Off
Number of isotope peaks used for i-FIT = 3

Monoisotopic Mass, Even Electron Ions
1 formula(e) evaluated with 1 results within limits (up to 50 best isotopic matches for each mass)
Elements Used:
C: 22-22 H: 12-13 N: 4-4
Hazal Kayas
44957_20250204_12-02 21 (0.829) Cm (14:24)
1: TOF MS ES+
5.34e+004

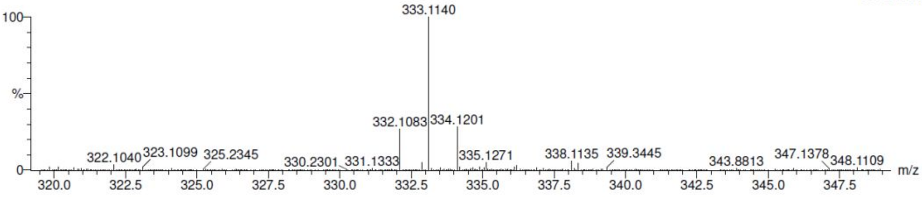

Minimum:				-1.5			
Maximum:		100.0	1000.0	50.0			
Mass	Calc. Mass	mDa	PPM	DBE	i-FIT	i-FIT (Norm)	Formula
333.1140	333.1140	0.0	0.0	18.5	426.2	0.0	C22 H13 N4

Figure S55. HR-MS spectrum of **12e**.

Elemental Composition Report **Page 1**

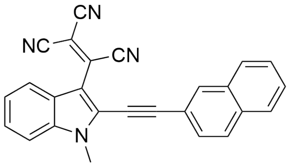

Single Mass Analysis
Tolerance = 1000.0 PPM / DBE: min = -5.5, max = 1000.0
Element prediction: Off
Number of isotope peaks used for i-FIT = 9

Monoisotopic Mass, Even Electron Ions
2 formula(e) evaluated with 1 results within limits (all results (up to 1000) for each mass)
Elements Used:
C: 26-26 H: 14-15 N: 4-4 Na: 0-1
Hazal Kayas
43096_20240814_13-02 14 (0.552) Cm (1:25)
1: TOF MS ES+
5.50e+003

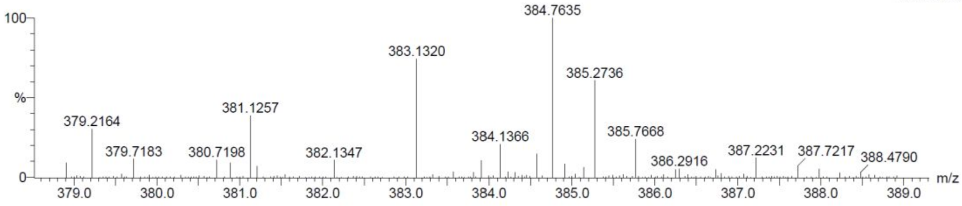

Minimum:				-5.5			
Maximum:		1000.0	1000.0	1000.0			
Mass	Calc. Mass	mDa	PPM	DBE	i-FIT	i-FIT (Norm)	Formula
383.1320	383.1297	2.3	6.0	21.5	796.4	0.0	C26 H15 N4

Figure S56. HR-MS spectrum of **12f**.

Elemental Composition Report **Page 1**

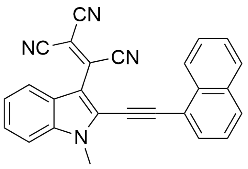

Single Mass Analysis
Tolerance = 1000.0 PPM / DBE: min = -1.5, max = 50.0
Element prediction: Off
Number of isotope peaks used for i-FIT = 3

Monoisotopic Mass, Odd and Even Electron Ions
1 formula(e) evaluated with 1 results within limits (up to 50 best isotopic matches for each mass)
Elements Used:
C: 26-26 H: 14-15 N: 4-4
Hazal Kayas
44957_20250204_09-03 4 (0.172) Cm (1:15)

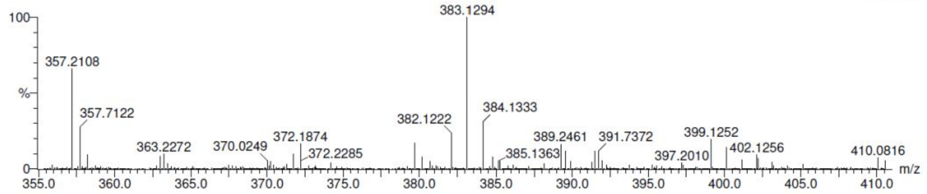

Minimum:				-1.5			
Maximum:		100.0	1000.0	50.0			
Mass	Calc. Mass	mDa	PPM	DBE	i-FIT	i-FIT (Norm)	Formula
383.1294	383.1297	-0.3	-0.8	21.5	333.0	0.0	C26 H15 N4

Figure S57. HR-MS spectrum of **12g**.

Elemental Composition Report **Page 1**

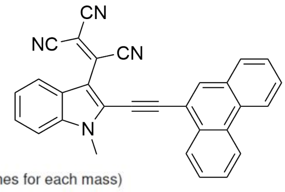

Single Mass Analysis
Tolerance = 1000.0 PPM / DBE: min = -1.5, max = 50.0
Element prediction: Off
Number of isotope peaks used for i-FIT = 3

Monoisotopic Mass, Odd and Even Electron Ions
1 formula(e) evaluated with 1 results within limits (up to 50 best isotopic matches for each mass)
Elements Used:
C: 30-30 H: 16-17 N: 4-4
Hazal Kayas
44957_20250204_14-01 13 (0.518) Cm (1:16)

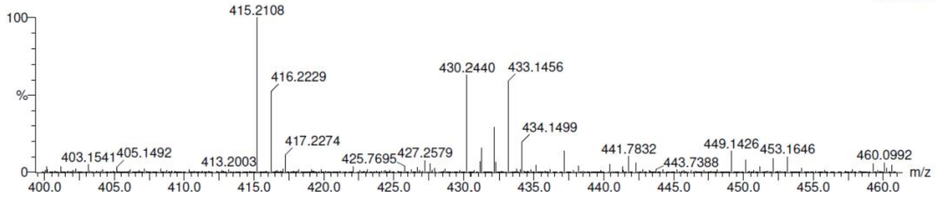

Minimum:				-1.5			
Maximum:		100.0	1000.0	50.0			
Mass	Calc. Mass	mDa	PPM	DBE	i-FIT	i-FIT (Norm)	Formula
433.1456	433.1453	0.3	0.7	24.5	375.4	0.0	C30 H17 N4

Figure S58. HR-MS spectrum of **12h**.

Elemental Composition Report Page 1

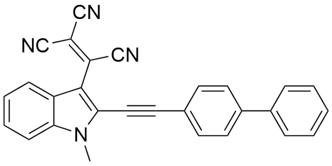

Single Mass Analysis
Tolerance = 1000.0 PPM / DBE: min = -5.5, max = 1000.0
Element prediction: Off
Number of isotope peaks used for i-FIT = 9

Monoisotopic Mass, Even Electron Ions
1 formula(e) evaluated with 1 results within limits (all results (up to 1000) for each mass)
Elements Used:
C: 28-28 H: 16-17 N: 4-4
Hazal Kayas
44757_20241011_01-09 11 (0.450) Cm (8:22)
1: TOF MS ES+
1.31e+003

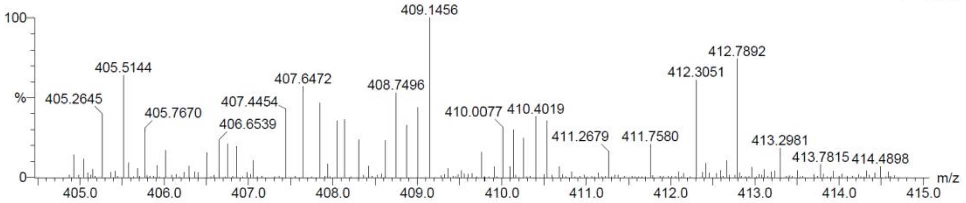

Minimum:				-5.5			
Maximum:		1000.0	1000.0	1000.0			
Mass	Calc. Mass	mDa	PPM	DBE	i-FIT	i-FIT (Norm)	Formula
409.1456	409.1453	0.3	0.7	22.5	674.3	0.0	C28 H17 N4

Figure S59. HR-MS spectrum of **12i**.

Elemental Composition Report Page 1

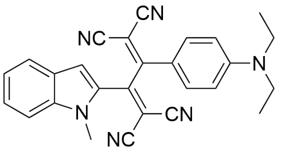

Single Mass Analysis
Tolerance = 1000.0 PPM / DBE: min = -1.5, max = 50.0
Element prediction: Off
Number of isotope peaks used for i-FIT = 3

Monoisotopic Mass, Odd and Even Electron Ions
1 formula(e) evaluated with 1 results within limits (up to 50 best isotopic matches for each mass)
Elements Used:
C: 26-27 H: 22-23 N: 6-6
Hazal Kayas
44957_20250204_08-01 3 (0.138) Cm (1:9)
1: TOF MS ES+
4.56e+004

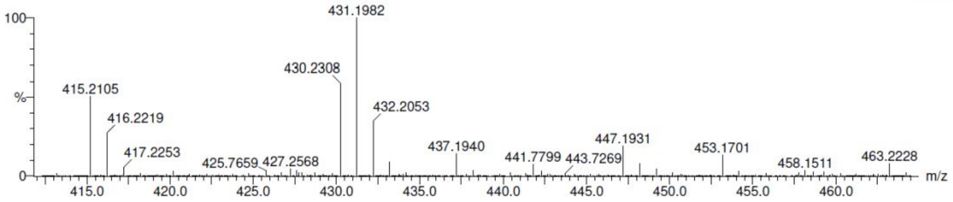

Minimum:				-1.5			
Maximum:		100.0	1000.0	50.0			
Mass	Calc. Mass	mDa	PPM	DBE	i-FIT	i-FIT (Norm)	Formula
431.1982	431.1984	-0.2	-0.5	19.5	357.5	0.0	C27 H23 N6

Figure S60. HR-MS spectrum of **13**.

Elemental Composition Report **Page 1**

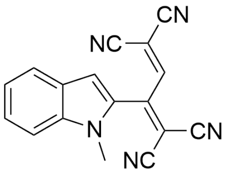

Single Mass Analysis
Tolerance = 30.0 PPM / DBE: min = -1.5, max = 50.0
Element prediction: Off
Number of isotope peaks used for i-FIT = 3

Monoisotopic Mass, Odd and Even Electron Ions
1 formula(e) evaluated with 1 results within limits (up to 50 best isotopic matches for each mass)
Elements Used:
C: 17-17 H: 8-10 N: 3-5
Hazal Kayas
46700_20250421_01-N04 18 (0.707) Cm (2:18) 1: TOF MS ES- 6.99e+004

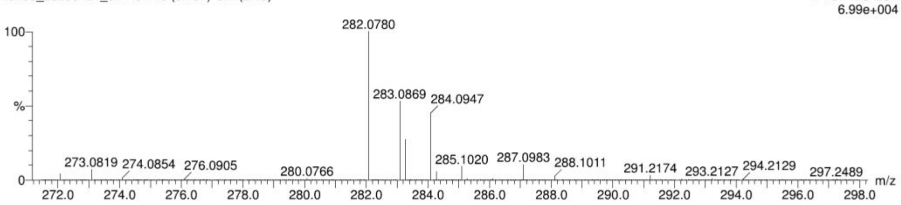

Mass	Calc. Mass	mDa	PPM	DBE	i-FIT	i-FIT (Norm)	Formula
Minimum:				-1.5			
Maximum:		100.0	30.0	50.0			
282.0780	282.0780	0.0	0.0	16.5	455.1	0.0	C17 H8 N5

Figure S61. HR-MS spectrum of **14**.

3. IR Spectra

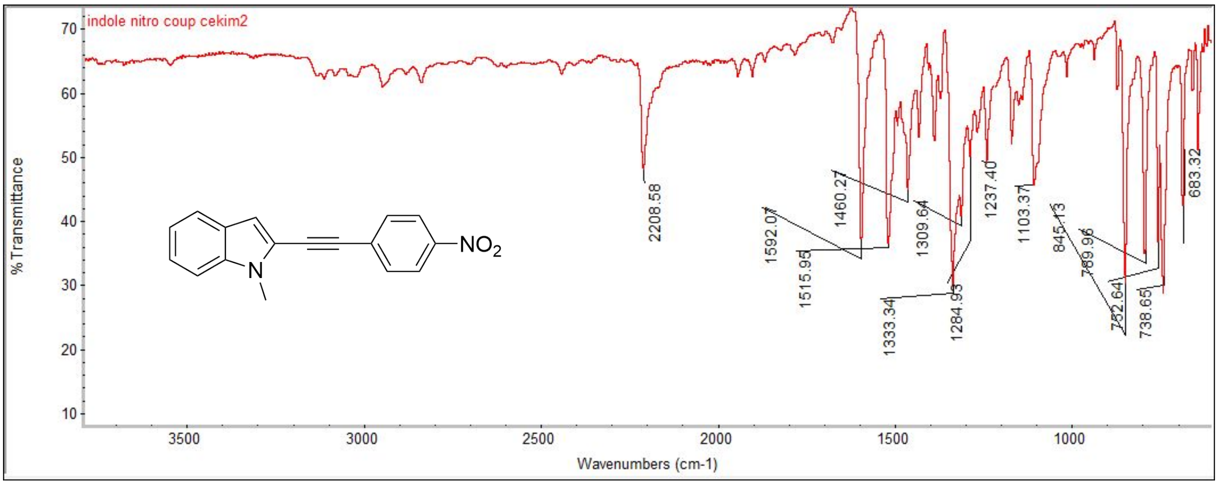

Figure S62. IR spectrum of **8a**.

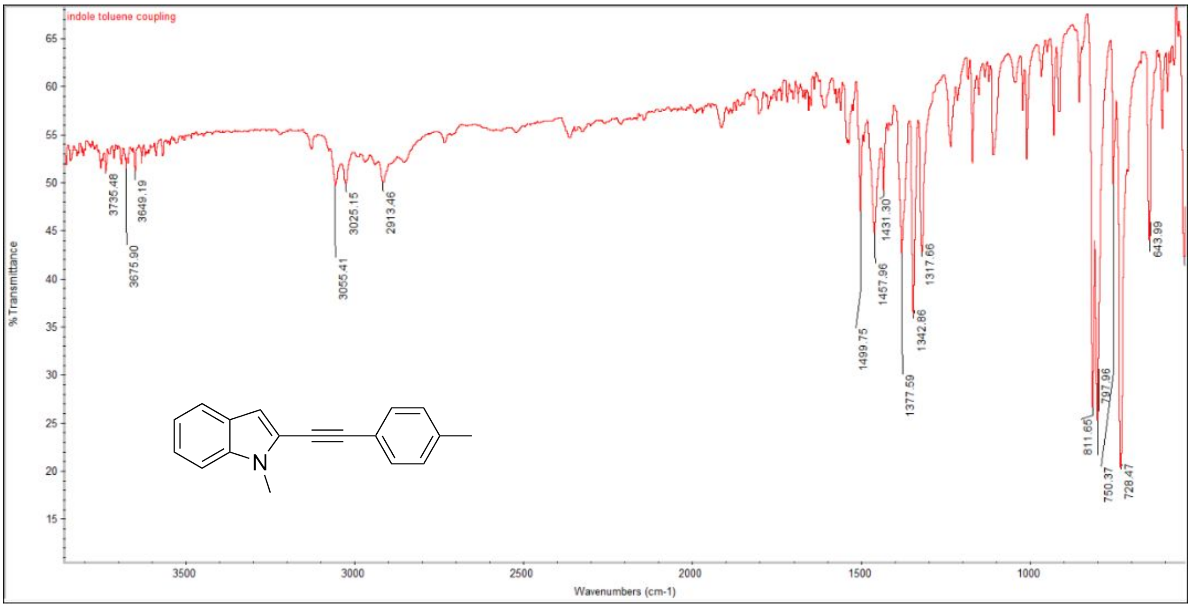

Figure S63. IR spectrum of **8b**.

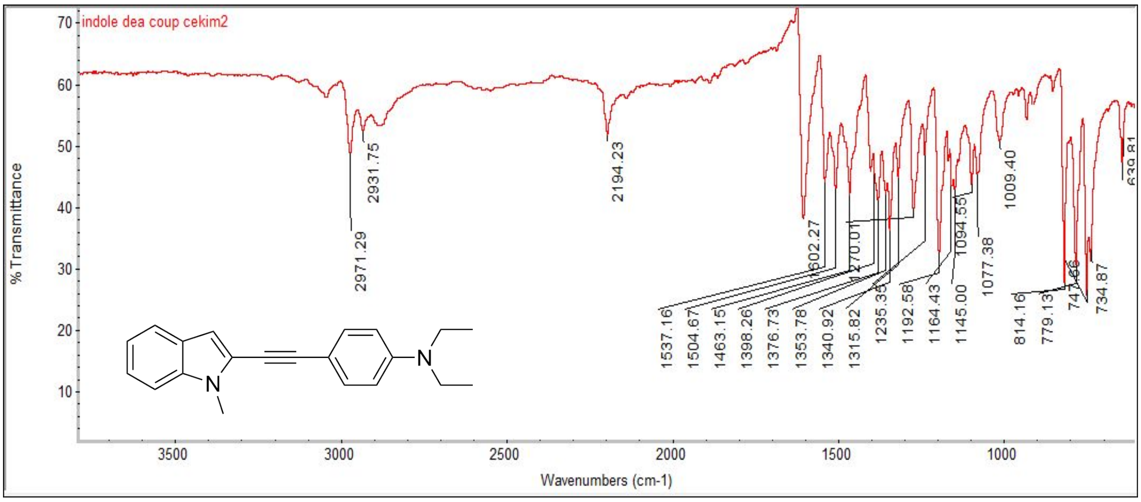

Figure S64. IR spectrum of **8d**.

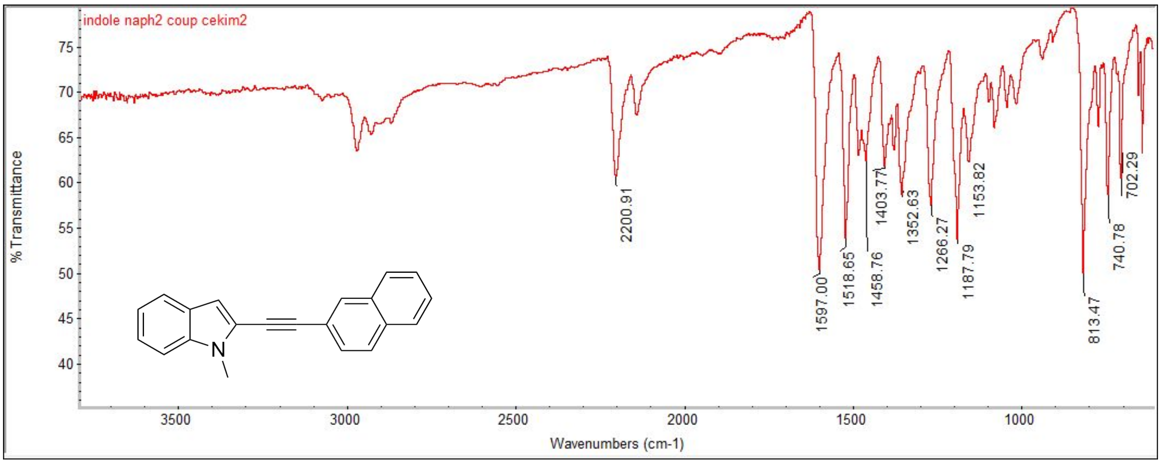

Figure S65. IR spectrum of **8f**.

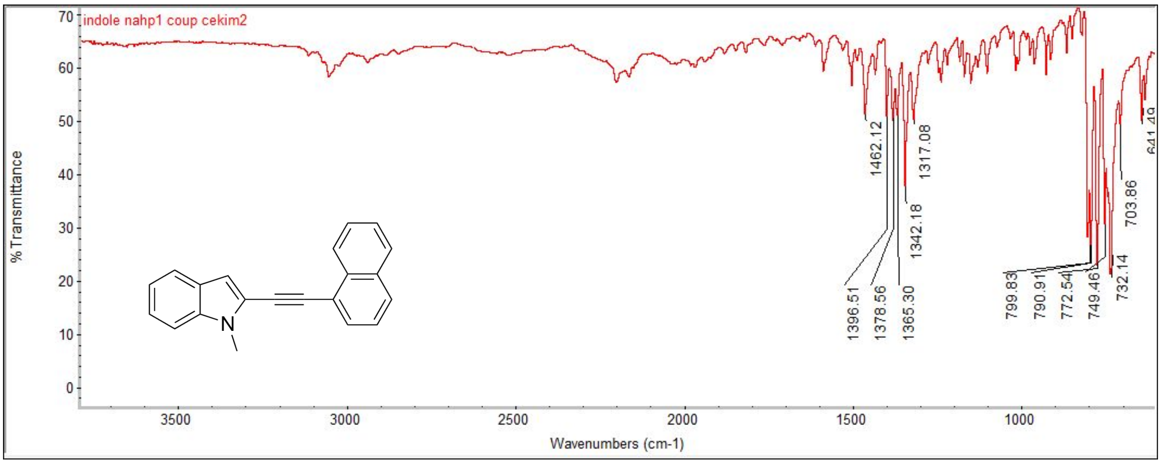

Figure S66. IR spectrum of **8g**.

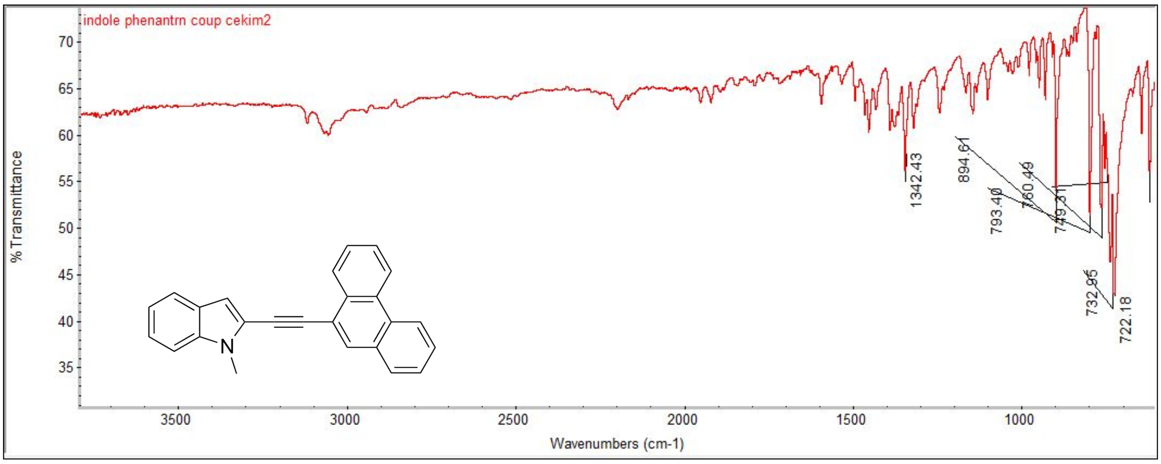

Figure S67. IR spectrum of **8h**.

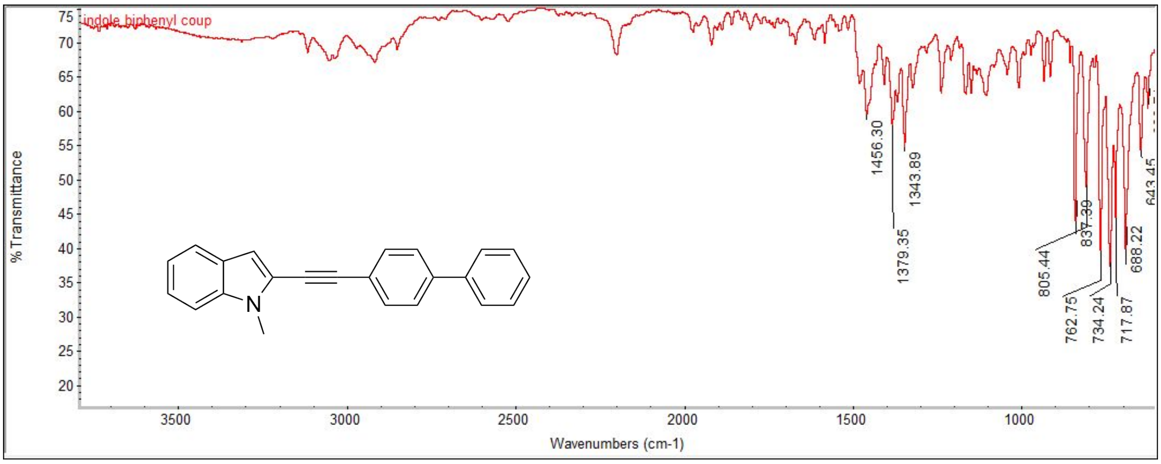

Figure S68. IR spectrum of **8i**.

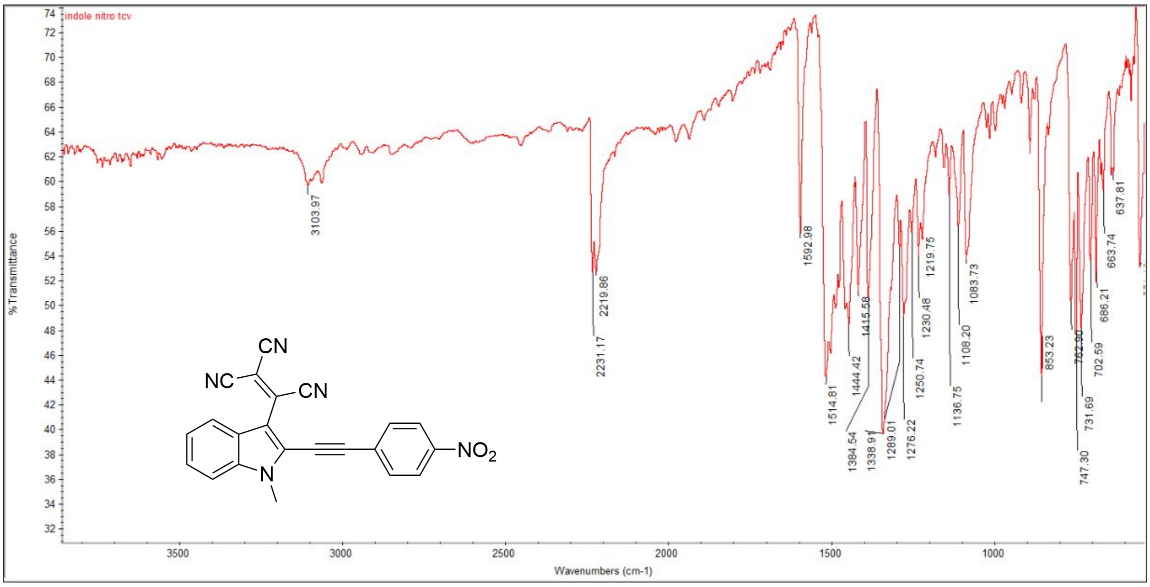

Figure S69. IR spectrum of **12a**.

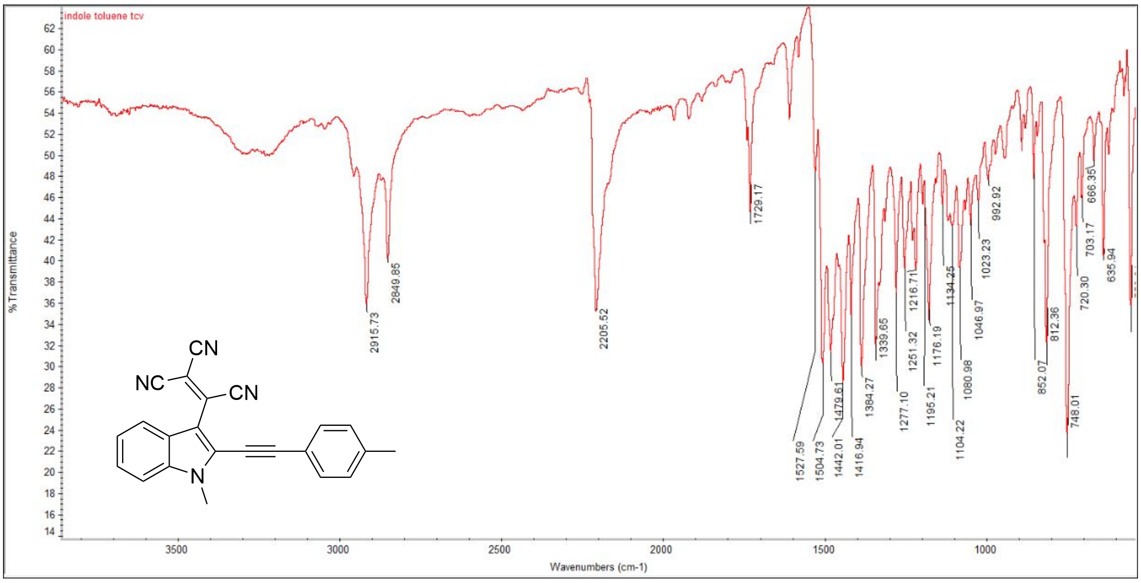

Figure S70. IR spectrum of **12b**.

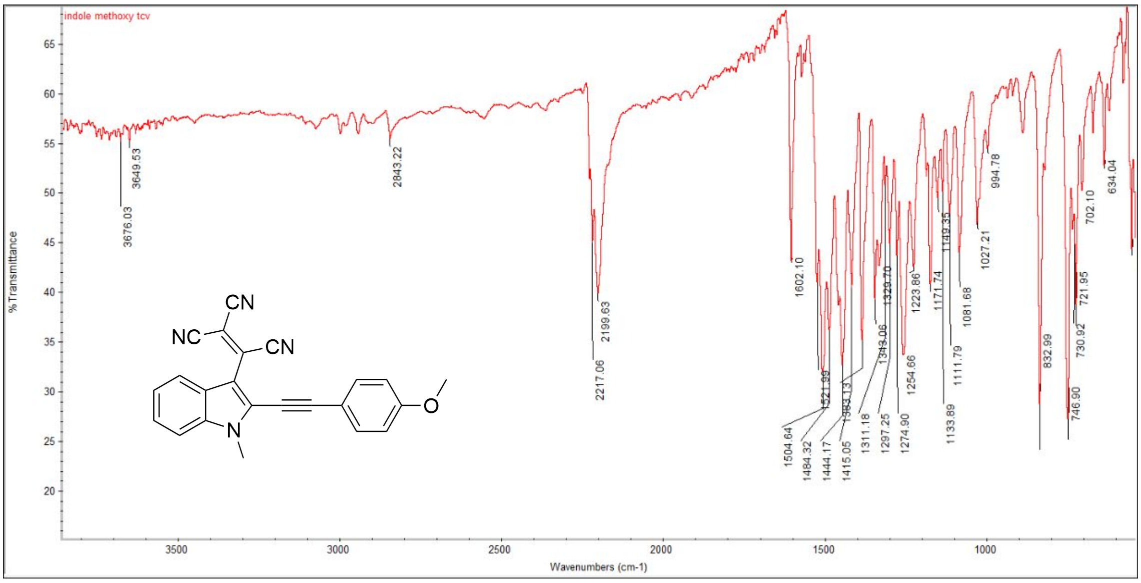

Figure S71. IR spectrum of **12c**.

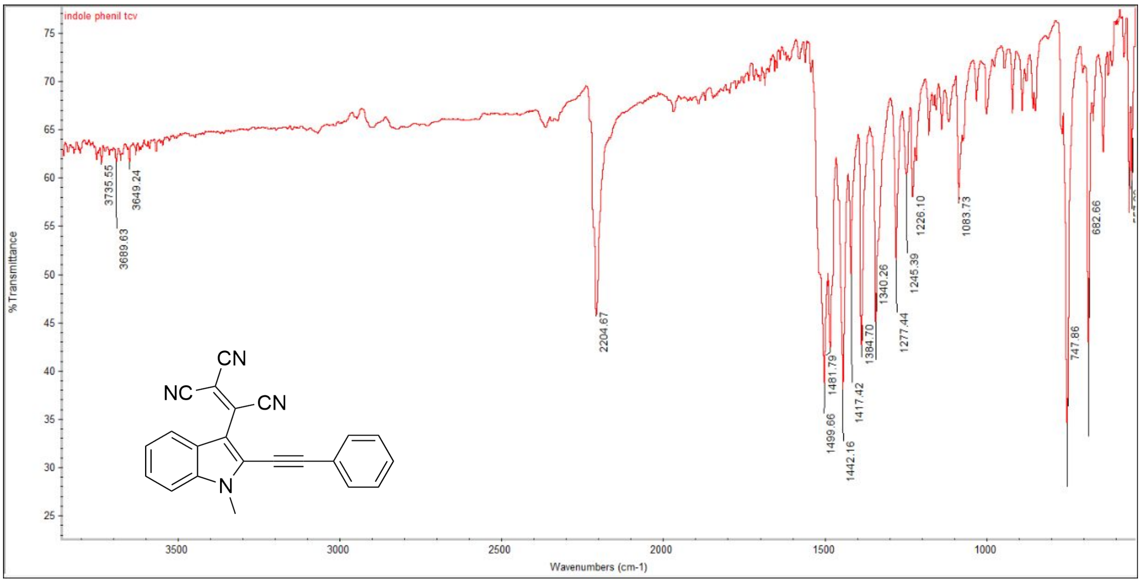

Figure S72. IR spectrum of **12e**.

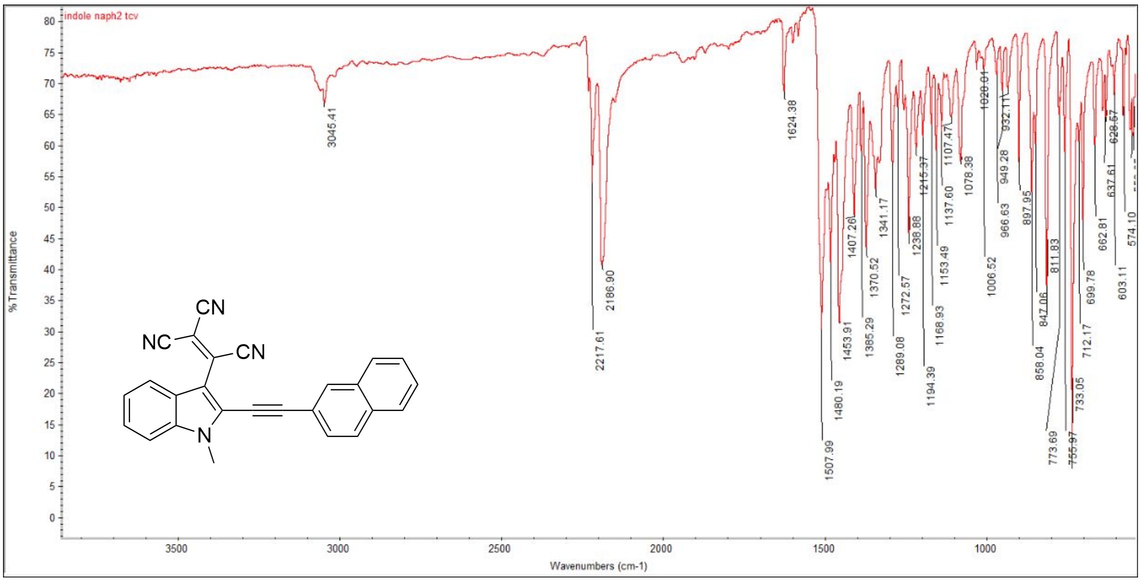

Figure S73. IR spectrum of **12f**.

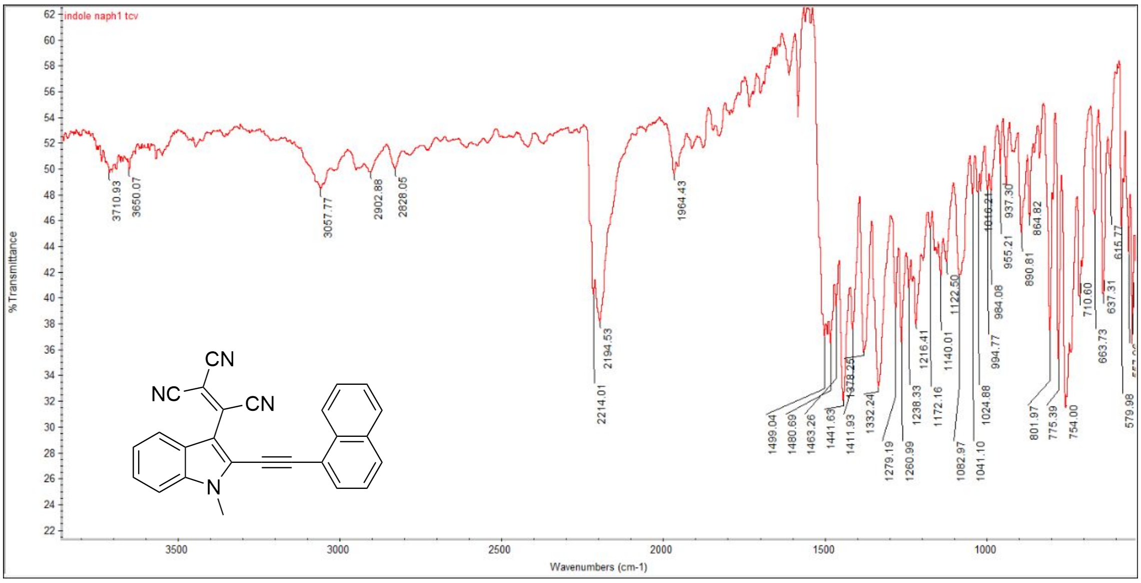

Figure S74. IR spectrum of **12g**.

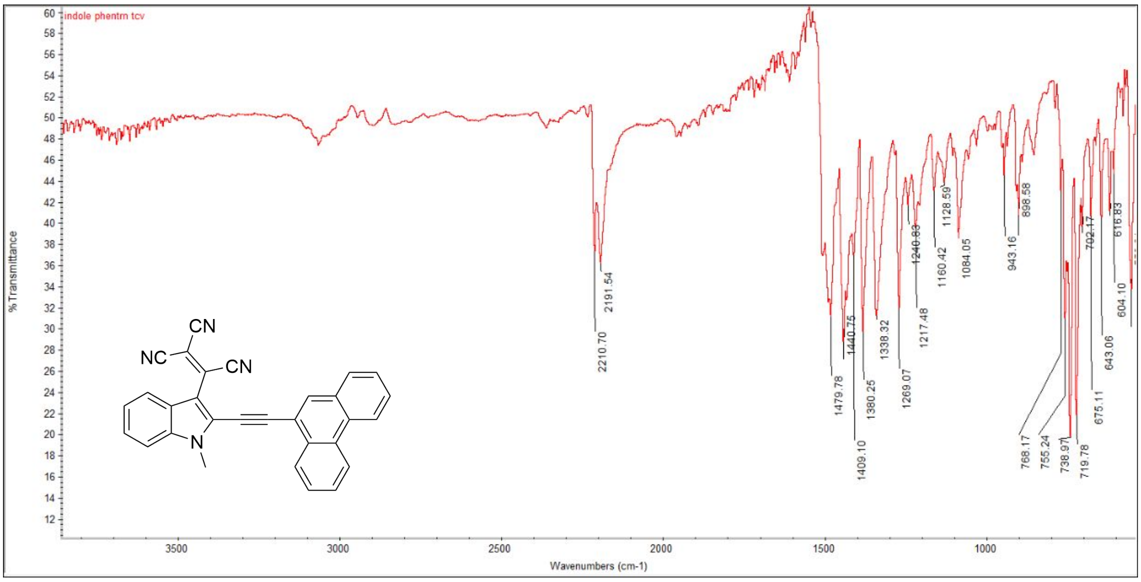

Figure S75. IR spectrum of **12h**.

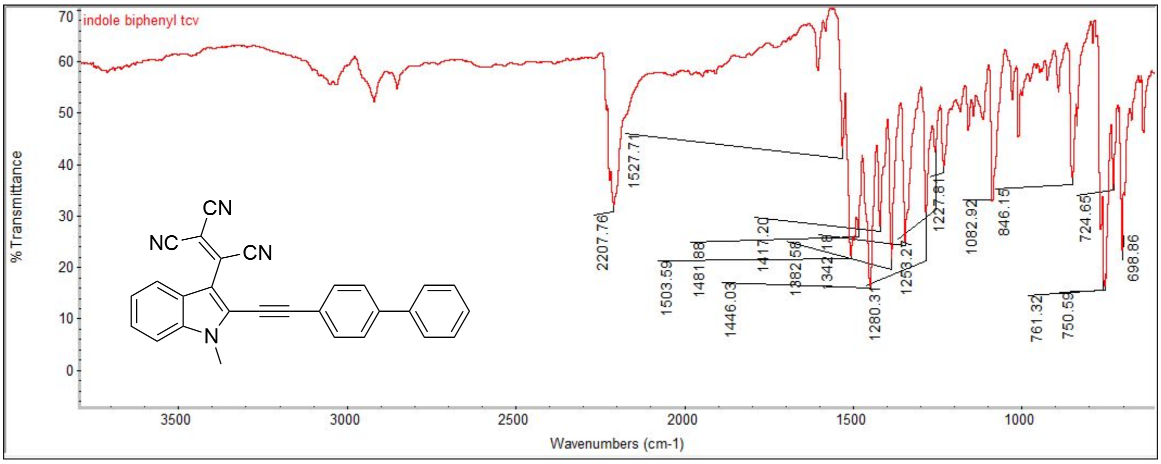

Figure S76. IR spectrum of **12i**.

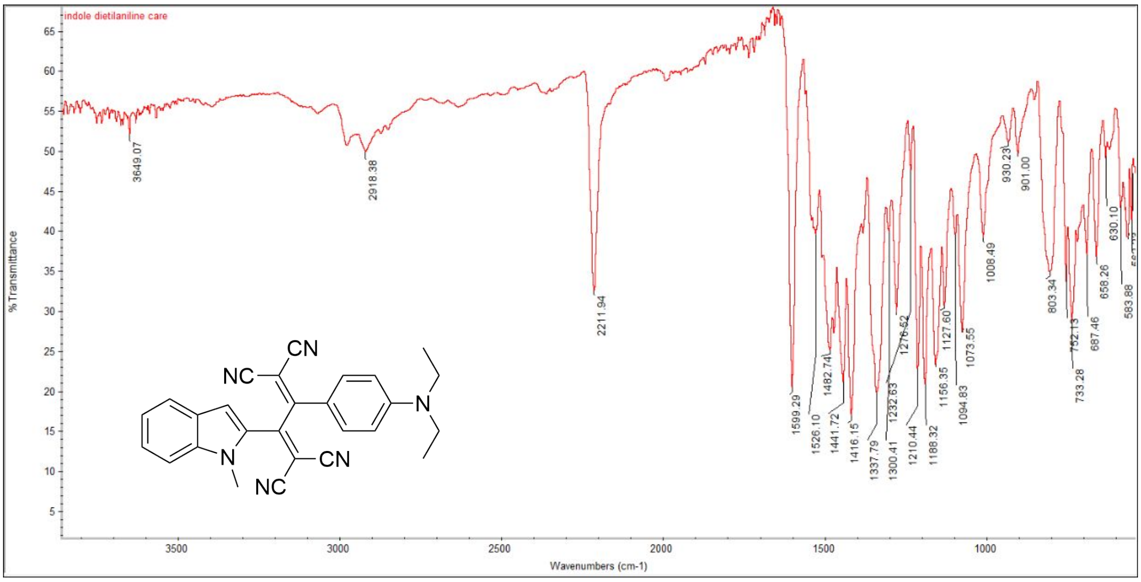

Figure S77. IR spectrum of **13**.

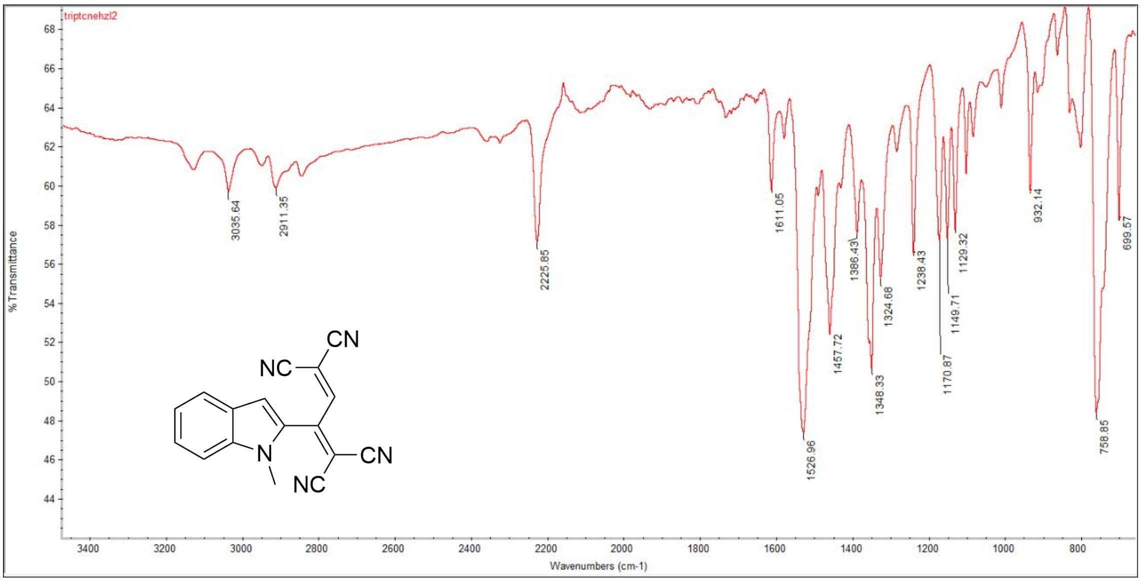

Figure S78. IR spectrum of **14**.

4. Theoretical Calculations

General Computational Methods

All molecular structures were geometry-optimized using the Gaussian 16 Rev. C.01. software package at the CAM-B3LYP/6-31++G(d,p) level of theory, employing the conductor-like solvation model of polarizable continuum model (CPCM) with CH_2Cl_2 as the solvent. The absence of imaginary frequencies confirmed that all optimized structures correspond to ground-state minima. For density functional theory (DFT) calculations, only conformers within an energy threshold of 3 kcal mol-1 above the global minimum were considered. Subsequently, vertical excitation energies were calculated using time-dependent density functional theory (TD-DFT) at the CAM-B3LYP/6-31++G(d,p) basis set with the CPCM CH_2Cl_2 solvation model. Frontier molecular orbital isosurfaces were also computed at the same level of theory and visualized with an isovalue of 0.02 a.u.

Table S1. Depiction of calculated HOMOs and LUMOs over optimized ground-state geometries, transition energies (E), and oscillator strengths (f) for **12a**.

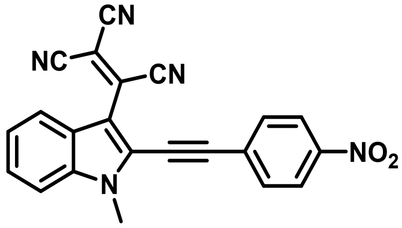

Exptl.: λ = 494 nm (in CH_2Cl_2)

**Excited state**	**Δ*E* (eV)**	**λ (nm)**	***f***	**assignments**
1	2.69	460	0.2621	H➔L
2	3.34	371	0.4454	H-1➔L
3	3.77	329	0.9682	H➔L+1, H-2➔L

**Orbital**		***E* (eV)**
**HOMO–1**	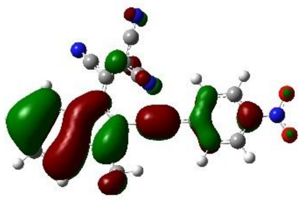	**-8.08**
**HOMO**	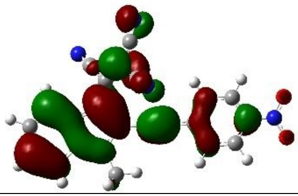	**-7.63**
**LUMO**	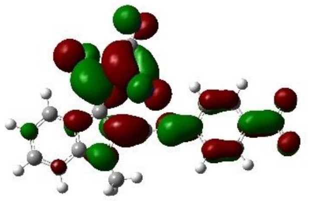	**-2.63**
**LUMO+1**	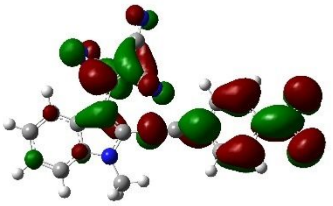	**-1.99**

12a

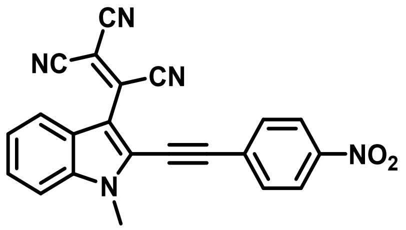

CAM-B3LYP/6-31G++(d,p) (CPCM solvation in DCM)

**Sum of electronic and zero-point Energies=**	**-1268.105985**
**Sum of electronic and thermal Energies=**	**-1268.081270**
**Sum of electronic and thermal Enthalpies=**	**-1268.080326**
**Sum of electronic and thermal Free Energies=**	**-1268.164168**
**Imaginary Freq = 0**	

Cartesian Coordinates

C	-6.11665400	-0.78069800	-0.20416200
C	-5.88393200	-2.16685100	-0.12836400
C	-4.60005600	-2.67404200	-0.02718200
C	-3.55052500	-1.75346100	-0.01317500
C	-3.75980200	-0.36368700	-0.09801600
C	-5.07156600	0.12614100	-0.18256800
N	-2.19252300	-1.99094000	0.08442500
C	-1.52799800	-0.80052400	0.07822300
C	-2.45688300	0.25009700	-0.01778500
C	-1.59430500	-3.31477300	0.12354700
C	-0.11932200	-0.73875600	0.03564100
C	1.08752900	-0.68066500	-0.02488700
C	2.50973300	-0.59343800	-0.09183700
C	3.29355900	-1.74887200	0.04704800
C	4.67448600	-1.66432800	-0.01502700
C	5.25639200	-0.41900600	-0.21780200
C	4.50596100	0.74157700	-0.35999700
C	3.12543400	0.65077900	-0.29629200
C	-2.15028300	1.64922200	-0.10590800
C	-1.17795500	2.32340000	0.59255900
C	-0.86905900	3.69167400	0.31263400
N	-0.61155300	4.79880200	0.09442300
N	0.06286100	1.33626500	2.63293200
C	-0.48374600	1.74818100	1.69938900
C	-2.94763100	2.42565600	-1.02347300
N	-3.58500100	3.03624200	-1.76998600
H	-7.13607300	-0.41742600	-0.27686400
H	-6.72657300	-2.84963900	-0.14481800
H	-4.42016900	-3.74083100	0.03989800
H	-5.27216800	1.19007600	-0.23105500
H	-1.80020300	-3.84863100	-0.80681200
H	-2.00235400	-3.87805900	0.96483500
H	-0.51848600	-3.21601800	0.25013000
H	2.81634700	-2.70864200	0.20488300
H	5.29481300	-2.54432600	0.09116500
H	4.99771300	1.69244600	-0.51663700
H	2.51828700	1.54145300	-0.40520700
N	6.71568200	-0.32654500	-0.28422800
O	7.21672500	0.77607400	-0.46710200
O	7.36606700	-1.35644000	-0.15388300

Table S2. Depiction of calculated HOMOs and LUMOs over optimized ground-state geometries, transition energies (E), and oscillator strengths (f) for **12b**.

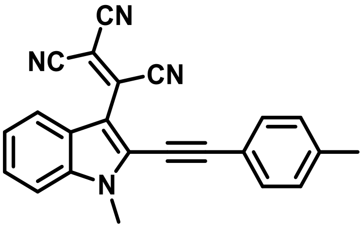

Exptl.: λ = 499 nm (in CH_2Cl_2)

**Excited state**	**ΔE (eV)**	**λ (nm)**	***f***	**assignments**
1	2.62	473	0.2485	**H➔L**
2	3.32	374	0.3837	**H-1➔L**
3	4.13	301	1.0568	**H➔L+1, H-2➔L**
4	4.37	284	0.0922	**H-2➔L**

**Orbital**		***E* (eV)**
**HOMO–1**	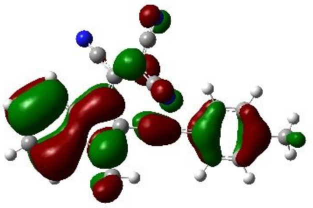	**-7.90**
**HOMO**	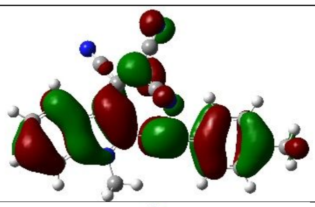	**-7.37**
**LUMO**	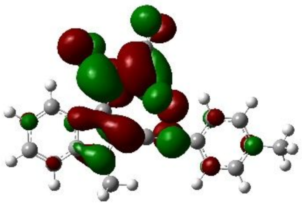	**-2.48**
**LUMO+1**	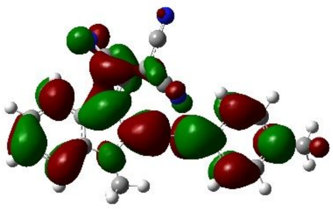	**-0.84**

12b

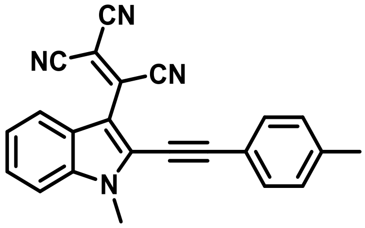

CAM-B3LYP/6-31G++(d,p) (CPCM solvation in DCM)

**Sum of electronic and zero-point Energies=**	**-1102.930185**
**Sum of electronic and thermal Energies=**	**-1102.90607**
**Sum of electronic and thermal Enthalpies=**	**-1102.905126**
**Sum of electronic and thermal Free Energies=**	**-1102.988414**
**Imaginary Freq = 0**	

Cartesian Coordinates

C	-5.57271400	-0.73394600	-0.15569600
C	-5.35221300	-2.11987100	-0.06816700
C	-4.07033300	-2.63659800	0.02810400
C	-3.01398900	-1.72567300	0.02463800
C	-3.21071500	-0.33561300	-0.07284500
C	-4.51794800	0.16401800	-0.15129800
N	-1.65535500	-1.97458000	0.11469700
C	-0.97743200	-0.79288600	0.09049400
C	-1.89931200	0.26784800	-0.00747600
C	-1.07014800	-3.30342900	0.15925000
C	0.42969100	-0.75076700	0.03013700
C	1.63774000	-0.71855300	-0.05385600
C	3.05951800	-0.66786800	-0.14972700
C	3.81674700	-1.84792700	-0.12906900
C	5.19957000	-1.78931500	-0.22383900
C	5.86683700	-0.56572000	-0.33987700
C	5.10265600	0.60542000	-0.36510900
C	3.71938000	0.56372700	-0.27048800
C	-1.57956200	1.65836100	-0.11438800
C	-0.57619000	2.32894600	0.54845700
C	-0.25319400	3.68654600	0.23866100
N	0.01606100	4.78620100	-0.00382600
N	0.68243700	1.36159200	2.58630200
C	0.12917700	1.76358800	1.65194100
C	-2.39475300	2.43992200	-1.01242300
N	-3.04445400	3.05626900	-1.74354300
H	-6.58910700	-0.36128000	-0.22386900
H	-6.20038200	-2.79602200	-0.07131400
H	-3.89890000	-3.70423400	0.10414400
H	-4.71030800	1.22899700	-0.20842700
H	-1.27633700	-3.83844200	-0.77060600
H	-1.48744400	-3.86103400	0.99986500
H	0.00586300	-3.21368000	0.29092000
H	3.31567200	-2.80588400	-0.04102200
H	5.77285100	-2.71142800	-0.20941900
H	5.59972100	1.56594700	-0.46215400
H	3.14261700	1.48191000	-0.29391700
C	7.36876300	-0.50885000	-0.41159400
H	7.70650400	0.35381300	-0.99067900
H	7.79920100	-0.41974400	0.59185200
H	7.77976800	-1.41344900	-0.86576800

Table S3. Depiction of calculated HOMOs and LUMOs over optimized ground-state geometries, transition energies (E), and oscillator strengths (f) for **12c**.

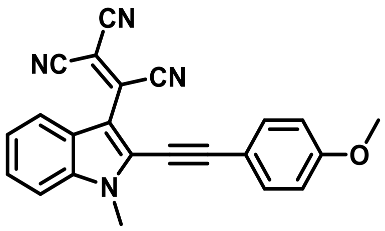

Exptl.: λ = 506 nm (in CH_2Cl_2)

**Excited state**	**Δ*E* (eV)**	***λ* (nm)**	***f***	**assignments**
1	2.57	483	0.2460	**H➔L**
2	3.28	378	0.4369	**H-1➔L**
3	4.04	307	0.8607	**H➔L+1**
4	4.24	293	0.3270	**H-2➔L, H➔L+1**

Orbital		*E* (eV)
HOMO–1	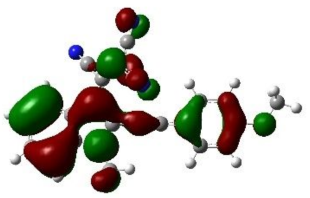	-7.84
HOMO	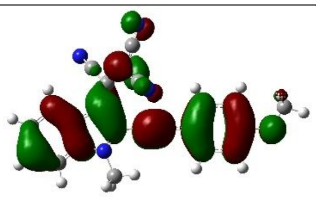	-7.23
LUMO	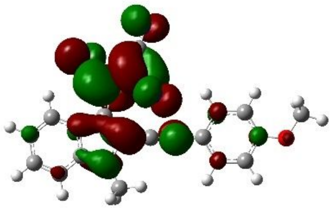	-2.44
LUMO+1	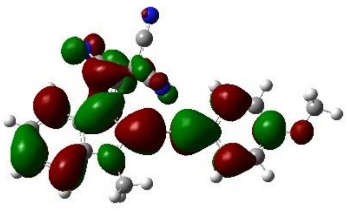	-0.77

12c

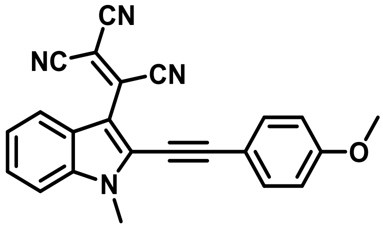

CAM-B3LYP/6-31G++(d,p) (CPCM solvation in DCM)

**Sum of electronic and zero-point Energies=**	**-1178.115685**
**Sum of electronic and thermal Energies=**	**-1178.090934**
**Sum of electronic and thermal Enthalpies=**	**-1178.089990**
**Sum of electronic and thermal Free Energies=**	**-1178.173273**
**Imaginary Freq = 0**	

Cartesian Coordinates

C	-5.91476300	-0.64629500	-0.20552000
C	-5.72154000	-2.03578600	-0.11643700
C	-4.45045700	-2.57639600	-0.00445700
C	-3.37755100	-1.68557600	0.00591800
C	-3.54675200	-0.29216200	-0.09292000
C	-4.84308300	0.23174400	-0.18703800
N	-2.02458300	-1.96013600	0.11315200
C	-1.32365000	-0.79196500	0.09860500
C	-2.22474900	0.28697700	-0.01001700
C	-1.46546500	-3.29984000	0.16333500
C	0.08367100	-0.77762100	0.05510200
C	1.29362800	-0.76952200	-0.01552100
C	2.71462800	-0.75356700	-0.09663600
C	3.44445500	-1.95662100	-0.09983700
C	4.82235700	-1.93930500	-0.17577200
C	5.50887200	-0.71993300	-0.25215100
C	4.79742400	0.48271800	-0.25249500
C	3.41073700	0.45835800	-0.17467000
C	-1.87768700	1.67002600	-0.11077700
C	-0.86488500	2.32003600	0.56024200
C	-0.51139200	3.67035500	0.25336400
N	-0.21705200	4.76421600	0.01324400
N	0.35277400	1.32767900	2.61060100
C	-0.18227100	1.74093300	1.67041800
C	-2.67190400	2.47061900	-1.01098300
N	-3.30396200	3.10297700	-1.74392800
H	-6.92294400	-0.25427700	-0.28603900
H	-6.58200900	-2.69606800	-0.13068700
H	-4.30015300	-3.64711700	0.07302500
H	-5.01486400	1.30014600	-0.24530400
H	-1.67284700	-3.83104000	-0.76846400
H	-1.90130900	-3.84938800	0.99985800
H	-0.38923800	-3.23065200	0.30528000
H	2.91865100	-2.90327700	-0.04079200
H	5.39311800	-2.86118300	-0.17739900
H	5.30672800	1.43552400	-0.31207000
H	2.86112300	1.39321700	-0.17519300
O	6.85704300	-0.80641700	-0.32284500
C	7.61852300	0.39465900	-0.39602400
H	8.66063200	0.08313500	-0.43976600
H	7.36881000	0.96345800	-1.29669300
H	7.45884500	1.01468500	0.49113900

Table S4. Depiction of calculated HOMOs and LUMOs over optimized ground-state geometries, transition energies (E), and oscillator strengths (f) for **12e**.

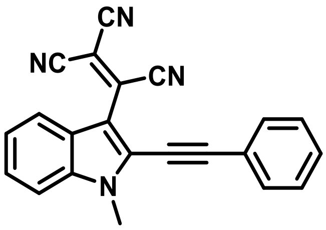

Exptl.: λ = 496 nm (in CH_2Cl_2)

**Excited state**	**Δ*E* (eV)**	***λ* (nm)**	***f***	**assignments**
1	2.66	467	0.2455	**H➔L**
2	3.34	372	0.3452	**H-1➔L**
3	4.16	298	1.0119	**H➔L+1**
4	4.43	280	0.0632	**H-3➔L**

**Orbital**		***E* (eV)**
**HOMO–1**	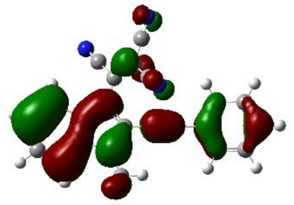	**-7.95**
**HOMO**	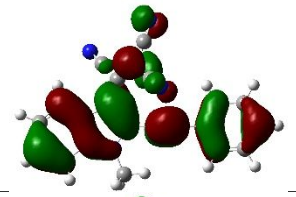	**-7.45**
**LUMO**	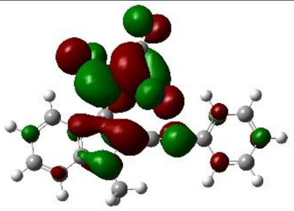	**-2.47**
**LUMO+1**	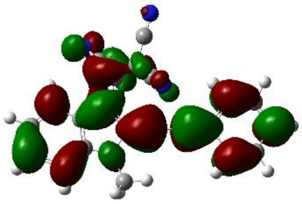	**-0.89**

12e

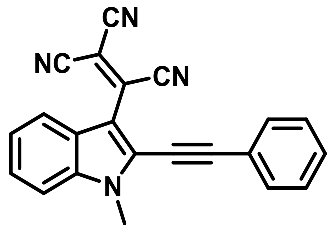

CAM-B3LYP/6-31G++(d,p) (CPCM solvation in DCM)

Sum of electronic and zero-point Energies= -1063.660976
Sum of electronic and thermal Energies= -1063.638818
Sum of electronic and thermal Enthalpies= -1063.637874
Sum of electronic and thermal Free Energies= -1063.715067
Imaginary Freq = 0

Cartesian Coordinates

C	-5.24218800	-0.58995400	-0.10375000
C	-5.06662500	-1.98324000	-0.02829000
C	-3.80169700	-2.54307100	0.04522200
C	-2.71540600	-1.66774400	0.03144200
C	-2.86739800	-0.27111100	-0.05347200
C	-4.15842900	0.27232000	-0.10942600
N	-1.36487400	-1.96209400	0.09813000
C	-0.64928200	-0.80285400	0.07202800
C	-1.53625400	0.28790500	-0.00417300
C	-0.82311500	-3.30985500	0.12615400
C	0.75806300	-0.80459700	-0.00968800
C	1.96469500	-0.80523100	-0.11082500
C	3.38718000	-0.79097700	-0.22878800
C	4.11359200	-1.98914500	-0.16303400
C	5.49744400	-1.96763700	-0.27741100
C	6.16712900	-0.75900000	-0.45908700
C	5.44917500	0.43355900	-0.52662000
C	4.06523600	0.42331700	-0.41230300
C	-1.17219200	1.66909400	-0.10363900
C	-0.14148000	2.30045600	0.55440900
C	0.22055200	3.65078000	0.25513900
N	0.52144700	4.74395000	0.02106900
N	1.11174500	1.27109800	2.56522200
C	0.55939300	1.70069400	1.64278600
C	-1.97052100	2.48381400	-0.98707200
N	-2.60785600	3.12638500	-1.70637800
H	-6.24653500	-0.18341800	-0.15437600
H	-5.93690500	-2.63065800	-0.02275900
H	-3.66515700	-3.61633800	0.11196600
H	-4.31602500	1.34346800	-0.15702000
H	-1.07675400	-3.83741500	-0.79605900
H	-1.23148400	-3.85335200	0.98036300
H	0.25900200	-3.25605000	0.22279000
H	3.58839500	-2.92740700	-0.02163700
H	6.05512900	-2.89665900	-0.22473500
H	7.24835500	-0.74652100	-0.54838400
H	5.96901300	1.37503800	-0.66870500
H	3.50250900	1.34881000	-0.46535000

Table S5. Depiction of calculated HOMOs and LUMOs over optimized ground-state geometries, transition energies (E), and oscillator strengths (f) for **12f**.

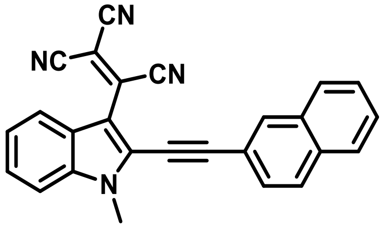

Exptl.: λ = 501 nm (in CH_2Cl_2)

**Excited state**	**Δ*E* (eV)**	**λ (nm)**	***f***	**assignments**
1	2.61	476	0.2134	**H→L**
2	3.29	377	0.4577	**H-1→L, H-2→L**
3	3.83	324	0.1161	**H-2→L**
4	4.04	307	1.0256	**H→L+1**

**Orbital**		***E* (eV)**
**HOMO–1**	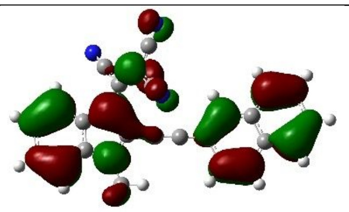	**-7.80**
**HOMO**	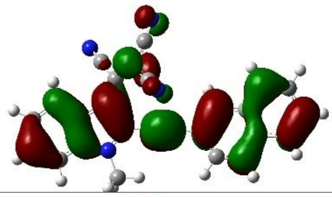	**-7.32**
**LUMO**	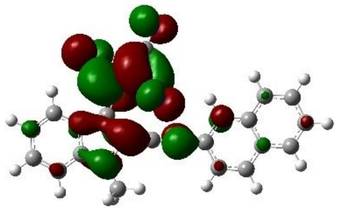	**-2.47**
**LUMO+1**	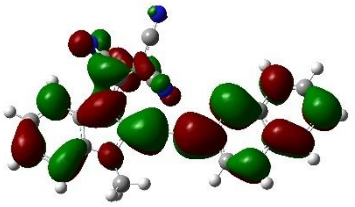	**-1.03**

12f

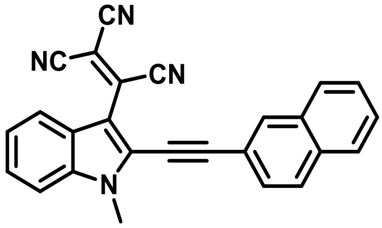

CAM-B3LYP/6-31G++(d,p) (CPCM solvation in DCM)

Sum of electronic and zero-point Energies= -1217.176806
Sum of electronic and thermal Energies= -1217.152092
Sum of electronic and thermal Enthalpies= -1217.151148
Sum of electronic and thermal Free Energies= -1217.234284
Imaginary Freq = 0

Cartesian Coordinates

C	-6.24989700	-0.38479600	-0.28242400
C	-6.14380000	-1.78587100	-0.22457600
C	-4.91118000	-2.40689400	-0.10645000
C	-3.78514600	-1.58464500	-0.05759600
C	-3.86721500	-0.18106200	-0.12403100
C	-5.12739200	0.42450300	-0.22561300
N	-2.45417700	-1.94431500	0.06223200
C	-1.68420900	-0.82034500	0.08779200
C	-2.51469300	0.31277200	-0.00694300
C	-1.97754300	-3.31643300	0.08862200
C	-0.27678200	-0.88784500	0.06669700
C	0.93215800	-0.93969100	0.01635700
C	-2.08200900	1.67626400	-0.06348900
C	-1.05246600	2.24604900	0.65053300
C	-0.61582400	3.58309700	0.39457200
N	-0.25515000	4.66515300	0.19587100
N	0.06088700	1.12319100	2.69336200
C	-0.42901600	1.59469500	1.75621600
C	-2.80208900	2.54419800	-0.96334000
N	-3.37646600	3.22966700	-1.69595500
H	-7.23076200	0.07012300	-0.36917500
H	-7.04302000	-2.39089600	-0.26853100
H	-4.82903100	-3.48645000	-0.05323000
H	-5.23186200	1.50263300	-0.26038000
H	-2.21662000	-3.81681300	-0.85242900
H	-2.44605400	-3.85354600	0.91555400
H	-0.89906800	-3.31543800	0.23056800
C	2.35572700	-0.99977100	-0.04156600
C	3.09678200	0.15431200	-0.20174500
C	3.01560800	-2.25960900	0.06454800
C	4.50967700	0.10506400	-0.26203400
H	2.59615900	1.11363800	-0.28443800
C	4.37933200	-2.32639400	0.01057400
H	2.42237800	-3.15848800	0.18974700
C	5.29104600	1.28006400	-0.42761100
C	5.16535300	-1.15347400	-0.15382100
H	4.88071000	-3.28575500	0.09321200
C	6.65862100	1.20556400	-0.48232200
H	4.78621500	2.23780300	-0.50997700
C	6.58156600	-1.19784700	-0.21435700
C	7.31060600	-0.04681900	-0.37460600
H	7.24826500	2.10753500	-0.60872500
H	7.07988100	-2.15907900	-0.13160800
H	8.39386700	-0.09165500	-0.41947000

Table S6. Depiction of calculated HOMOs and LUMOs over optimized ground-state geometries, transition energies (E), and oscillator strengths (f) for **12g**.

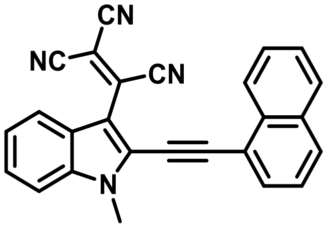

Exptl.: λ = 504 nm (in CH_2Cl_2)

Excited state	ΔE (eV)	λ (nm)	f	assignments
1	2.58	481	0.1993	**H➔L**
2	3.24	382	0.4245	**H-1➔L**
3	3.77	329	0.1711	**H-2➔L**
4	3.96	313	0.7076	**H➔L+1, H-2➔L**

Orbital		E (eV)
**HOMO–1**	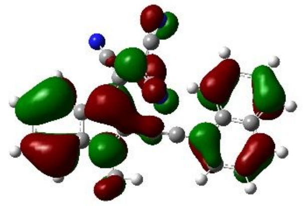	**-7.82**
**HOMO**	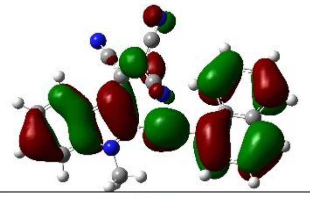	**-7.28**
**LUMO**	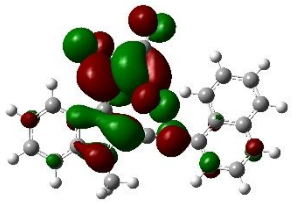	**-2.49**
**LUMO+1**	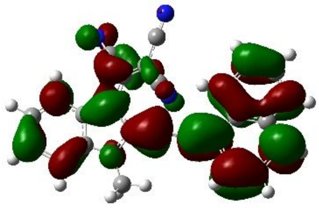	**-1.10**

12g

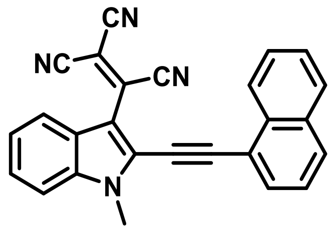

CAM-B3LYP/6-31G++(d,p) (CPCM solvation in DCM)

Sum of electronic and zero-point Energies= -1217.175726
Sum of electronic and thermal Energies= -1217.151041
Sum of electronic and thermal Enthalpies= -1217.150097
Sum of electronic and thermal Free Energies= -1217.232829
Imaginary Freq = 0

Cartesian Coordinates

C	-5.93118200	-0.10581800	-0.38048600
C	-5.91879600	-1.51073400	-0.44288400
C	-4.73364200	-2.22139400	-0.34842200
C	-3.55851200	-1.48416800	-0.19965400
C	-3.54615200	-0.07785200	-0.14566100
C	-4.76067000	0.61790800	-0.22509500
N	-2.25863200	-1.94264400	-0.07798300
C	-1.41721300	-0.88008500	0.06461000
C	-2.16812700	0.31044200	0.04926900
C	-1.87563600	-3.34154300	-0.16254700
C	-0.01694400	-1.04758400	0.07509100
C	1.18313800	-1.21069100	0.05567300
C	-1.65035100	1.64255900	0.13844700
C	-0.61695900	2.07700300	0.93705500
C	-0.10105900	3.40642300	0.83333400
N	0.32440500	4.48047800	0.75790000
N	0.37342100	0.69077100	2.87907900
C	-0.06485400	1.28228500	1.98549100
C	-2.28517700	2.63704900	-0.69206500
N	-2.79065500	3.42472700	-1.37065200
H	-6.87706100	0.42034500	-0.45160400
H	-6.85385600	-2.04738100	-0.56226000
H	-4.72319900	-3.30463700	-0.38712000
H	-4.79405300	1.69958900	-0.16807800
H	-2.11646000	-3.73888400	-1.15107800
H	-2.40669100	-3.91523400	0.59943800
H	-0.80495400	-3.42821600	0.00830000
C	2.59394600	-1.42013900	0.04755200
C	3.48737200	-0.36593500	-0.33875500
C	3.09284600	-2.65515900	0.41899100
C	3.03363700	0.91476100	-0.74283300
C	4.88590200	-0.62368800	-0.31729800
C	4.48037200	-2.89660600	0.43311900
H	2.40521400	-3.44247900	0.70744100
C	3.92559100	1.89331600	-1.10213400
H	1.96810100	1.11355500	-0.77064300
C	5.78353400	0.41043100	-0.69324700
C	5.35620500	-1.90369900	0.07531700
H	4.84803900	-3.87240300	0.73105700
C	5.31692100	1.64097700	-1.07582800
H	3.56218400	2.86836500	-1.40960100
H	6.84983000	0.20645900	-0.67258200
H	6.42664100	-2.08498600	0.08710800
H	6.01135900	2.42422600	-1.36104000

Table S7. Depiction of calculated HOMOs and LUMOs over optimized ground-state geometries, transition energies (E), and oscillator strengths (f) for **12h**.

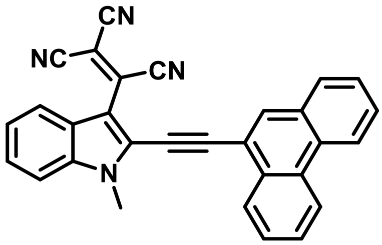

Exptl.: λ = 504 nm (in CH_2Cl_2)

Excited state	ΔE (eV)	λ (nm)	f	assignments
1	2.58	480	0.2041	**H→L**
2	3.26	380	0.4653	**H-1→L**
3	3.79	327	0.4404	**H-3→L**
4	3.90	318	0.2859	**H-2→L, H→L+1**

Orbital		E (eV)
**HOMO–1**	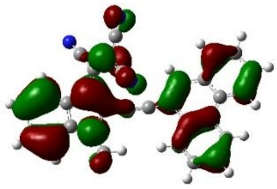	**-7.81**
**HOMO**	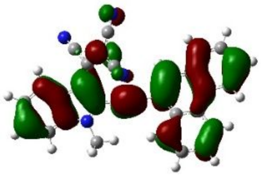	**-7.24**
**LUMO**	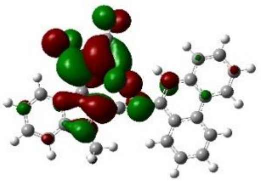	**-2.47**
**LUMO+1**	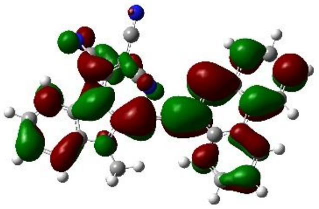	**-1.13**

12h

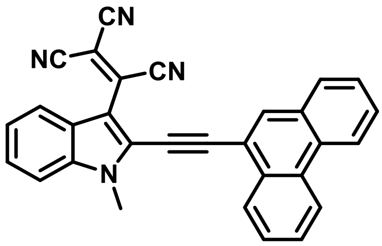

CAM-B3LYP/6-31G++(d,p) (CPCM solvation in DCM)

**Sum of electronic and zero-point Energies=**	**-1370.693793**
**Sum of electronic and thermal Energies=**	**-1370.666352**
**Sum of electronic and thermal Enthalpies=**	**-1370.665408**
**Sum of electronic and thermal Free Energies=**	**-1370.754495**
**Imaginary Freq = 0**	

Cartesian Coordinates

C	-6.65848600	-0.65620300	-0.35003700	H	-2.38578200	-3.82600400	0.44651700
C	-6.41811400	-2.03554500	-0.48347300	H	-1.04109900	-3.07428500	-0.44066300
C	-5.13018600	-2.54452700	-0.45554700	C	1.95877400	-0.31824300	-0.16218900
C	-4.08713900	-1.63095300	-0.30090300	C	2.45094100	0.92271300	-0.44515700
C	-4.30390700	-0.24616800	-0.17354900	C	2.86140400	-1.42905500	0.08088000
C	-5.61769800	0.24285600	-0.18742700	C	3.85546300	1.17228300	-0.52091900
N	-2.72619100	-1.87298500	-0.23826800	H	1.76708200	1.74592100	-0.62441200
C	-2.06714200	-0.69475800	-0.05328600	C	4.25907300	-1.21338000	0.00843600
C	-3.00337500	0.35499800	0.00908600	C	2.36952600	-2.71300500	0.39121700
C	-2.12215800	-3.18437600	-0.39724400	C	4.32927700	2.46939400	-0.81883600
C	-0.65984600	-0.61393100	-0.07355500	C	4.77210000	0.11597300	-0.30040100
C	0.54588700	-0.50331400	-0.11135300	C	5.11302200	-2.31214100	0.24602900
C	-2.70372500	1.74974300	0.13717000	H	1.29807200	-2.86452000	0.45560800
C	-1.72738700	2.31596700	0.92445500	C	3.22684200	-3.76608900	0.61891100
C	-1.42322600	3.71103000	0.85034200	C	5.67868800	2.72404300	-0.89881800
N	-1.16980400	4.83948600	0.79849200	H	3.60787100	3.26394000	-0.98380400
N	-0.49370200	1.04469200	2.80476700	C	6.14975100	0.40674900	-0.39003100
C	-1.03430300	1.58677700	1.93624100	C	4.61308000	-3.56153300	0.54309100
C	-3.51157600	2.65591600	-0.64244200	H	6.18674400	-2.18220900	0.19798700
N	-4.15593800	3.37357200	-1.27983500	H	2.83178100	-4.74796500	0.85730900
H	-7.67969100	-0.29108400	-0.36977000	C	6.59369500	1.67952300	-0.68185900
H	-7.25626800	-2.71335600	-0.60447000	H	6.03594500	3.72248200	-1.12748600
H	-4.94417200	-3.60857200	-0.54601900	H	6.88389600	-0.37255900	-0.22859600
H	-5.82489300	1.30052400	-0.07321500	H	5.29490900	-4.38647100	0.72117300
H	-2.46991300	-3.64155200	-1.32548900	H	7.65943700	1.87390000	-0.74374800

Table S8. Depiction of calculated HOMOs and LUMOs over optimized ground-state geometries, transition energies (E), and oscillator strengths (f) for **12i**.

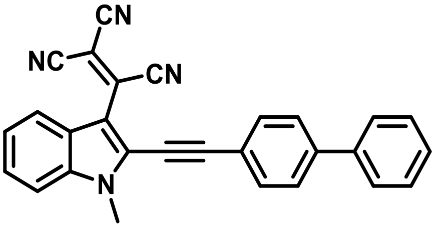

Exptl.: λ = 503 nm (in CH_2Cl_2)

**Excited state**	**Δ*E* (eV)**	**λ (nm)**	***f***	**assignments**
1	2.62	474	0.2666	**H➔L**
2	3.30	375	0.4709	**H-1➔L**
3	3.99	311	1.3172	**H➔L+2**
4	4.25	292	0.1484	**H-2➔L+1**

**Orbital**		***E* (eV)**
**HOMO–1**	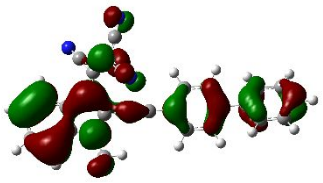	**-7.86**
**HOMO**	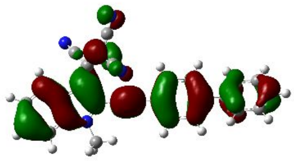	**-7.32**
**LUMO**	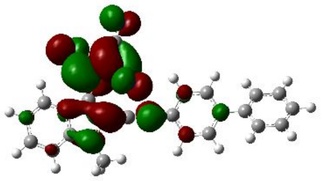	**-2.74**
**LUMO+1**	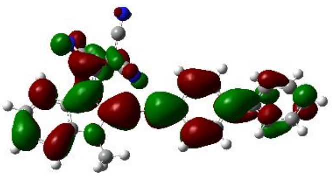	**-0.95**

12i

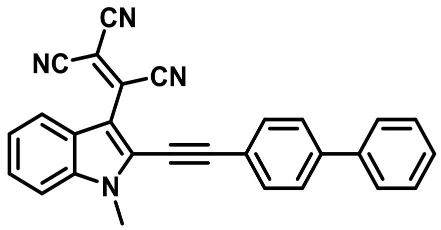

CAM-B3LYP/6-31G++(d,p) (CPCM solvation in DCM)

Sum of electronic and zero-point Energies = -1294.516273
Sum of electronic and thermal Energies = -1294.489482
Sum of electronic and thermal Enthalpies = -1294.488538
Sum of electronic and thermal Free Energies = -1294.576977
Imaginary Freq = 0

Cartesian Coordinates

C	-6.93348300	-0.85174200	-0.29391700	H	-2.53882600	-3.81433800	-0.91906200
C	-6.67655300	-2.23387700	-0.25541900	H	-2.79235200	-3.91089800	0.84336100
C	-5.38375800	-2.71958700	-0.14745100	H	-1.30069800	-3.19701700	0.19770200
C	-4.35317500	-1.78118100	-0.08896300	C	1.69060300	-0.55061000	-0.04705200
C	-4.58616100	-0.39400900	-0.13747100	C	2.31785700	0.70139600	-0.11990500
C	-5.90472700	0.07329900	-0.22830500	C	2.48093900	-1.70850600	-0.02076800
N	-2.99110400	-1.99691900	0.02609500	C	3.70064900	0.78674700	-0.16193600
C	-2.34633600	-0.79701400	0.06507300	H	1.71657500	1.60370100	-0.13497400
C	-3.29433500	0.24111300	-0.01604700	C	3.86286700	-1.61068300	-0.06371000
C	-2.37028600	-3.31032600	0.03535900	H	2.00584400	-2.68221900	0.02676500
C	-0.93963000	-0.71391300	0.04561700	C	4.49870400	-0.36429100	-0.13394100
C	0.26870900	-0.64422200	0.00074800	H	4.16789400	1.76494300	-0.19646500
C	-3.01032600	1.64349400	-0.06167400	H	4.45675500	-2.51830000	-0.06234200
C	-2.04706200	2.31403400	0.65715400	C	5.97909300	-0.26637700	-0.17663500
C	-1.75281100	3.69093800	0.40956800	C	6.61129700	0.69162800	-0.97830900
N	-1.50731400	4.80597200	0.21766600	C	6.77563900	-1.12962700	0.58536000
N	-0.82101400	1.30023100	2.69250700	C	7.99880000	0.78334400	-1.01757800
C	-1.35850900	1.72429500	1.75877500	H	6.01449800	1.35782600	-1.59291300
C	-3.81863700	2.43588900	-0.95628900	C	8.16313100	-1.03776900	0.54707600
N	-4.46285500	3.06084100	-1.68493800	H	6.30678200	-1.86567900	1.23055000
H	-7.95804300	-0.50429200	-0.37232700	C	8.78043300	-0.08084400	-0.25473900
H	-7.50535700	-2.93180500	-0.30606900	H	8.46982200	1.52777300	-1.65154000
H	-5.18475000	-3.78442800	-0.10857100	H	8.76261500	-1.71099000	1.15152700
H	-6.12512800	1.13425400	-0.24785400	H	9.86279900	-0.00899800	-0.28482800

Table S9. Depiction of calculated HOMOs and LUMOs over optimized ground-state geometries, transition energies (E), and oscillator strengths (f) for **13**.

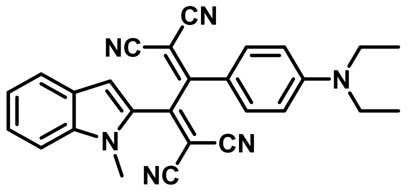

Exptl.: λ = 420 nm (in CH_2Cl_2)

Excited state	ΔE (eV)	λ (nm)	f	assignments
1	2.97	418	0.3160	**H➔L**
2	3.07	404	0.7667	**H➔L+1**
3	3.22	385	0.3102	**H-1➔L**
4	3.54	350	0.2374	**H-2➔L**

Orbital		*E* (eV)
**HOMO–1**	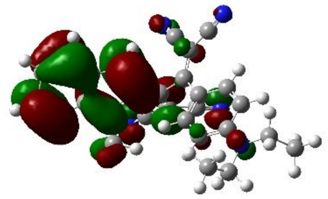	**-7.64**
**HOMO**	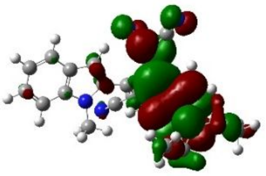	**-7.11**
**LUMO**	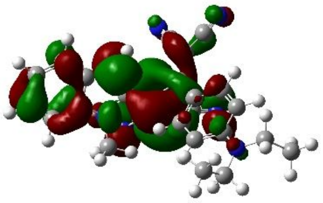	**-2.11**
**LUMO+1**	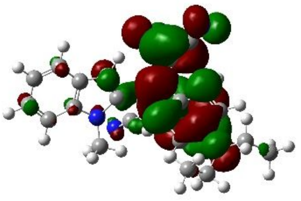	**-1.83**

13

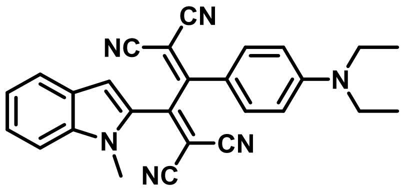

CAM-B3LYP/6-31G++(d,p) (CPCM solvation in DCM)

Sum of electronic and zero-point Energies = -1369.441320

Sum of electronic and thermal Energies = -1369.410688

Sum of electronic and thermal Enthalpies = -1369.409743

Sum of electronic and thermal Free Energies = -1369.504687

Imaginary Freq = 0

Cartesian Coordinates

C	-6.59250700	1.61881600	-0.13120400	N	1.22889800	-2.72638800	2.46588400
C	-5.91951000	2.29902200	-1.17457700	N	-1.96185600	-0.50322500	4.21611000
C	-4.56757300	2.13390900	-1.39938500	C	5.28915200	3.45625800	0.16391600
C	-3.87664600	1.25668900	-0.54833400	C	6.92616700	-0.75079500	-0.07772800
C	-4.53104900	0.57698100	0.50684500	H	-7.65679000	1.77934500	0.00352100
C	-5.91427200	0.76170900	0.70666600	H	-6.48462300	2.96464600	-1.81889800
N	-2.55286800	0.90532200	-0.54791700	H	-4.07084400	2.65308000	-2.21079400
C	-2.34352300	0.01102500	0.50408000	H	-6.42644400	0.23974300	1.50836100
C	-3.54569700	-0.19609900	1.16233700	H	-3.71109200	-0.88771700	1.97399400
C	-1.58156900	1.51247900	-1.44479900	H	-1.59136700	1.02959600	-2.42518100
C	-1.10241800	-0.68432700	0.76115300	H	-1.82109400	2.56993300	-1.56479400
C	-0.17022400	-1.01355300	-0.37056600	H	-0.58141300	1.44819300	-1.02627200
C	1.18601500	-0.52833400	-0.28627100	H	2.11041300	-2.06682200	-1.50575000
C	-0.69154100	-1.76393100	-1.40371000	H	4.33740700	-1.23366500	-1.34631900
C	-0.01799700	-2.00299200	-2.64097800	H	2.93506900	2.00164800	1.17097400
C	2.26692100	-1.15181500	-0.94968300	H	0.71899600	1.12506900	1.04180500
C	-0.75620500	-1.12557500	2.01272900	H	6.93192600	1.20681400	-0.98690500
C	3.55102200	-0.67706500	-0.85602100	H	5.85307600	0.08223400	-1.77187900
C	3.85866900	0.48894600	-0.09944300	H	6.51285700	1.96771400	1.13626100
C	-1.99003700	-2.36120100	-1.33967900	H	4.88798400	2.04600200	1.76567100
C	0.35570700	-2.00250300	2.23397100	H	5.59290200	4.25821300	0.84247700
C	-1.45649600	-0.77278600	3.20998000	H	5.90326000	3.52360100	-0.73840700
C	2.77520100	1.10936300	0.58228400	H	4.24753300	3.62510700	-0.12105400
C	1.50129300	0.60539700	0.49927700	H	7.75611400	-1.11925600	-0.68703000
N	5.12028800	0.97256100	-0.02115400	H	7.33055300	-0.42134300	0.88342700
C	6.22245500	0.39823000	-0.79432700	H	6.24268700	-1.58296300	0.10902200
C	5.46877700	2.10128100	0.84288300				
N	0.46641500	-2.19932100	-3.67513200				
N	-3.02684700	-2.87808700	-1.33046800				

5. X-ray Diffraction Analysis

Suitable crystal of **12i** (CCDC 2516047) was selected for data collection which was performed on a Bruker D8-QUEST diffractometer equipped with a graphite-monochromatic Mo-K_{α} radiation at 293 K. We used these procedures for our analysis: solved by direct methods; SHELXS-2013[1]; refined by full-matrix least-squares methods; SHELXL-2013[2]; data collection: Bruker APEX2[3]; molecular graphics: MERCURY[4;] solution: WinGX[5]. Details of data collection and crystal structure determinations are given in Table 1.

Table S10. Crystal data and structure refinement parameters for **12i** (CCDC 2516047).

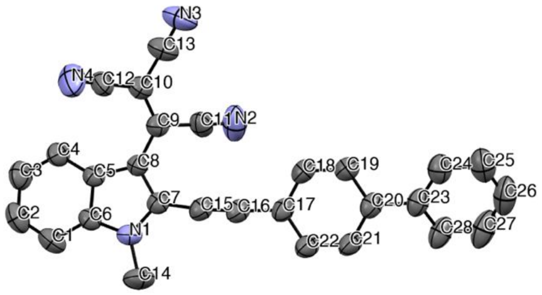

Empirical formula	$C_{28}H_{16}N_4$
Formula weight	408.45
Crystal system	Monoclinic
Space group	$P2_1/n$
a (Å)	7.4793 (5)
b (Å)	19.6628 (13)
c (Å)	14.8527 (10)
β (º)	95.883 (2)
V (Å^3)	2172.8 (3)
Z	4
D_c (g cm^{-3})	1.249
μ (mm^{-1})	0.08
θ range (º)	2.5-26.5
Measured refls.	79771
Independent refls.	4487
R_{int}	0.081
S	1.09
R1/wR2	0.053/0.156
$\Delta\rho_{max}/\Delta\rho_{min}$ (eÅ^{-3})	0.34/-0.20
CCDC	2516047

6. UV/Vis Studies

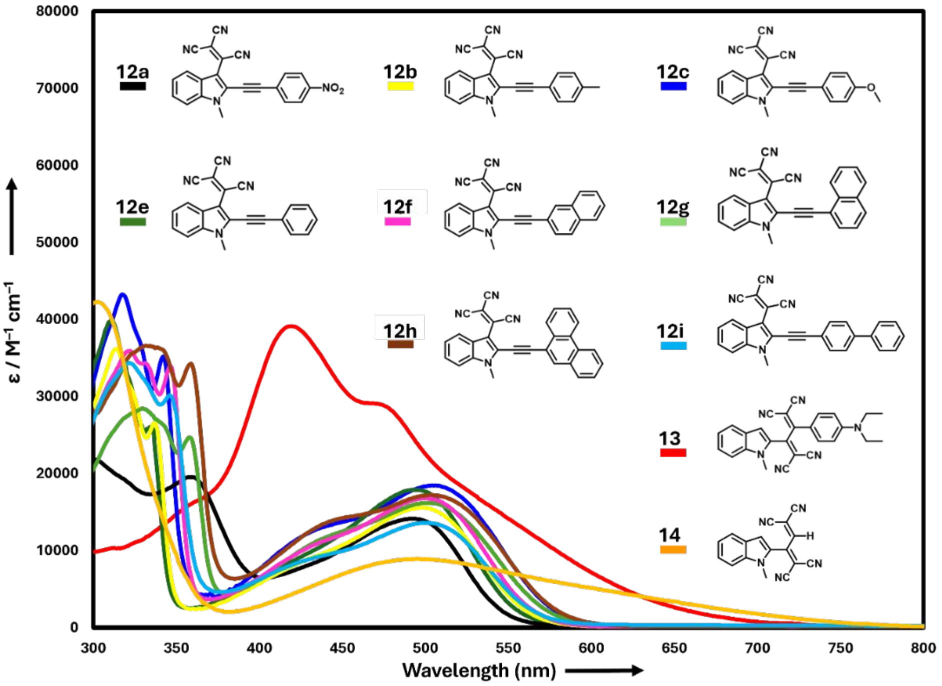

Figure S79. UV/vis absorption spectra of **12a-i**, **13** and **14** in CH_2Cl_2 at 298 K.

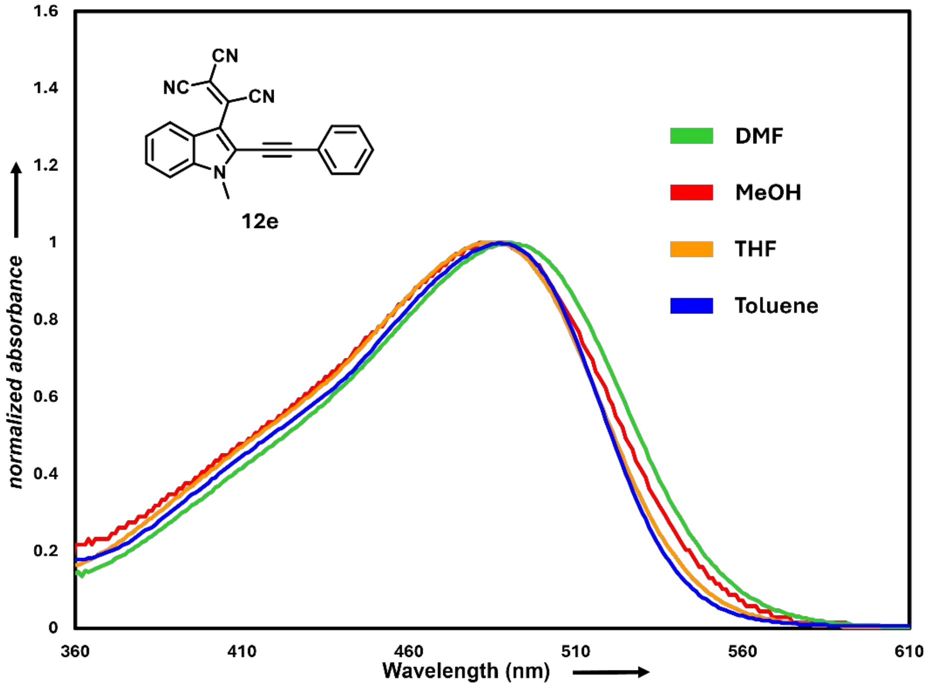

Figure S80. UV/vis absorption spectra of **12e** in different solvents at 298 K.

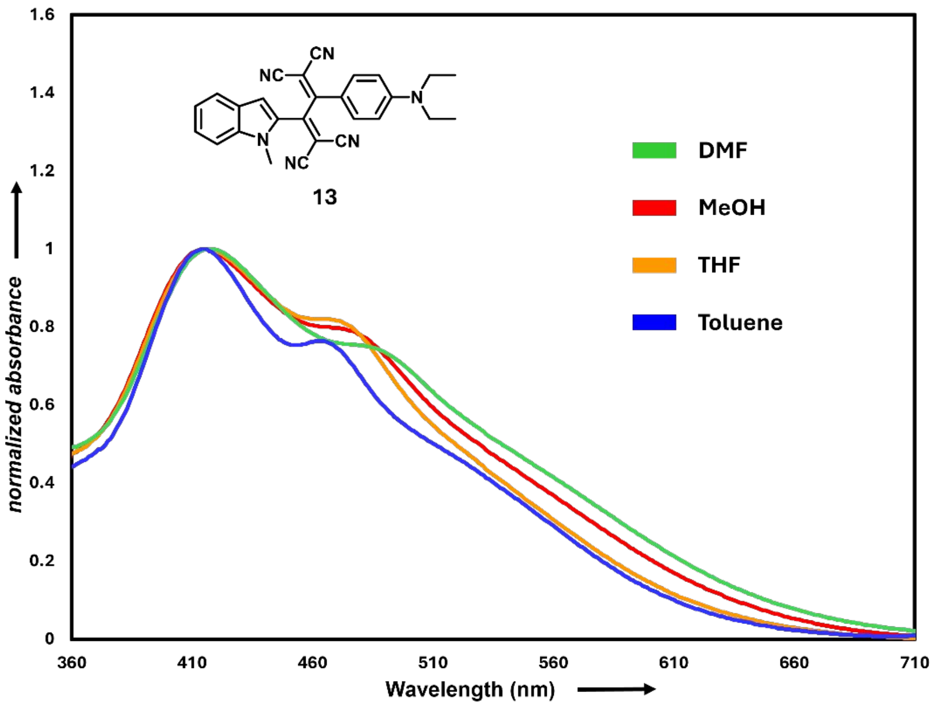

Figure S81. UV/vis absorption spectra of **13** in different solvents at 298 K.

7. Thermal Gravimetric Analysis (TGA) Studies

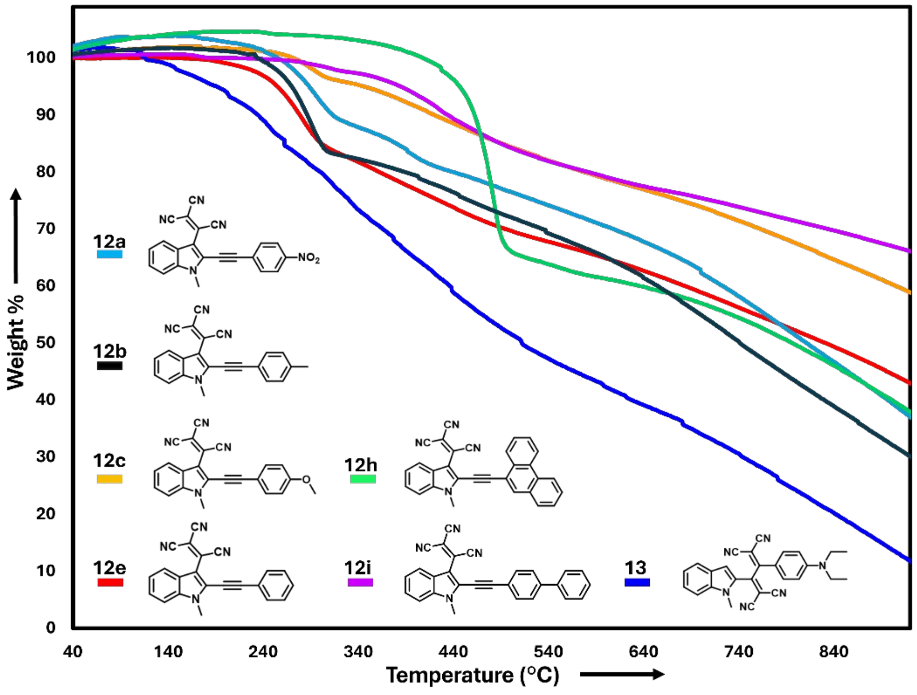

Figure S82. Thermogravimetric analysis curves of **12a**–**12c**, **12e**, **12h**, **12i** and **13**.

8. Images

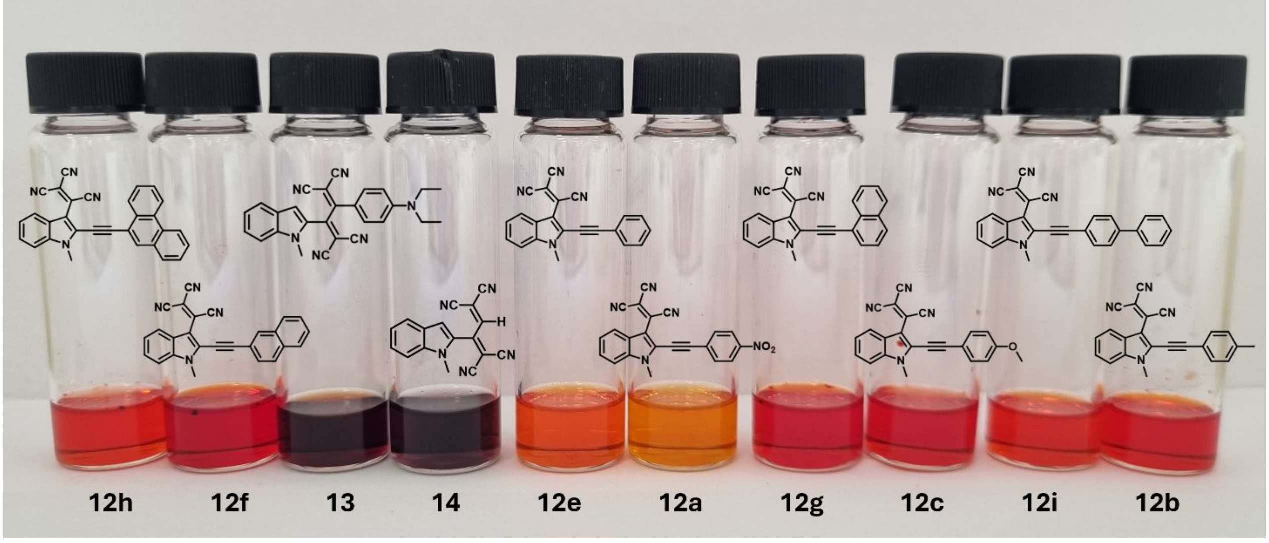

Figure S83. Photographs of chromophores **12a**–**12c**, **12e**–**i**, **13** and **14** solutions in $CHCl_3$.

9. Proposed Mechanism

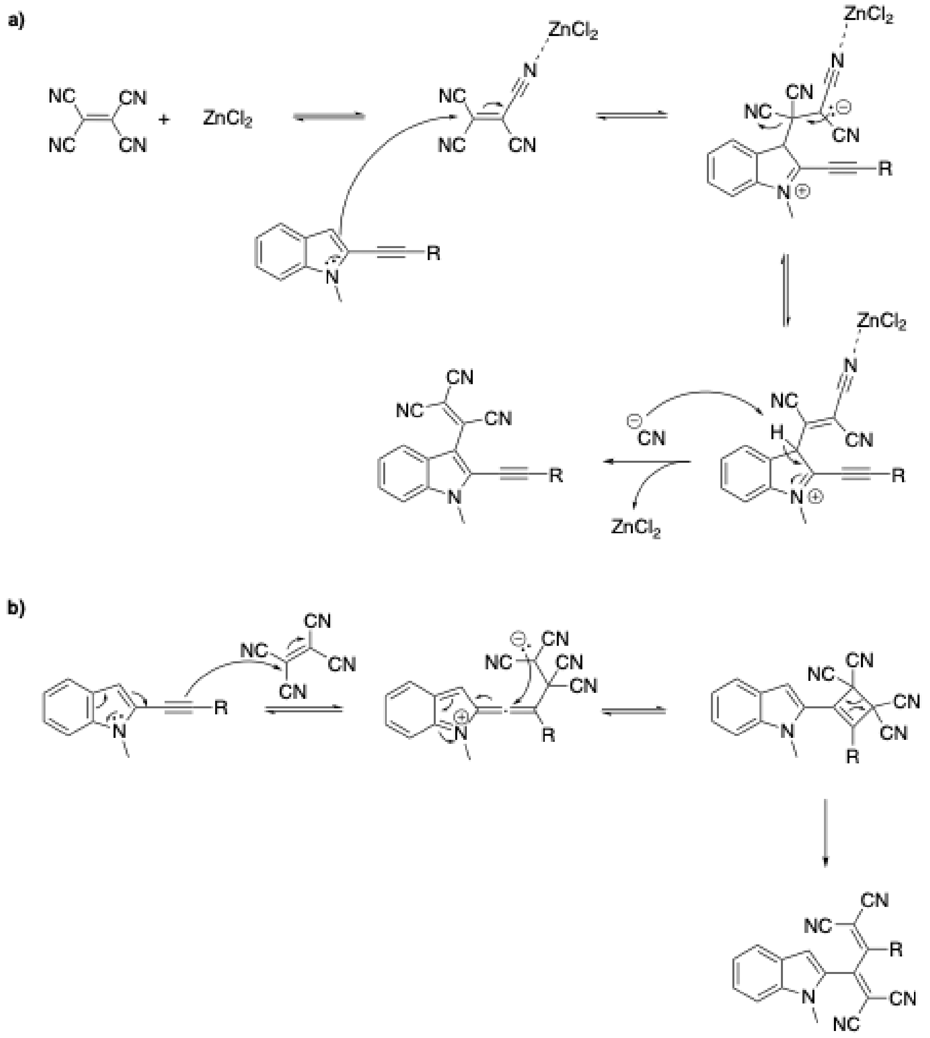

Scheme S1. Proposed mechanisms for (a) $ZnCl_2$-promoted tricyanovinylation and (b) formal [2+2] cycloaddition–retroelectrocyclization (CA–RE).

10. References

[1] Sheldrick, G.M. *Acta Cryst.* **2008***, A64*, 112.

[2] Sheldrick, G. M. Acta Cryst. **2015***, C71*, 3.

[3] APEX2, Bruker AXS Inc. Madison Wisconsin USA, **2013**.

[4] Macrae, C. F., Sovago, I., Cottrell, S. J., Galek, P. T. A., McCabe, P., Pidcock, E., Platings, M., Shields, G. P., Stevens, J. S., Towler, M., Wood, P. A. *J. Appl. Cryst.* **2020**, *53*, 226–235.

[5] Farrugia, L. J. *J. Appl. Cryst.* **2012***, 45*, 849–854.
